# Phylogenetic and syntenic data support a single horizontal transference to a *Trypanosoma* ancestor of a prokaryotic proline racemase implicated in parasite evasion from host defences

**DOI:** 10.1186/s13071-015-0829-y

**Published:** 2015-04-12

**Authors:** Zuleima C Caballero, Andre G Costa-Martins, Robson C Ferreira, João M P Alves, Myrna G Serrano, Erney P Camargo, Gregory A Buck, Paola Minoprio, Marta M G Teixeira

**Affiliations:** Departamento de Parasitologia, Instituto de Ciências Biomédicas, Universidade de São Paulo, São Paulo, SP 05508-900 Brazil; Instituto de Investigaciones Científicas y Servicios de Alta Tecnología-AIP (INDICASAT-AIP), Ciudad del Saber, Clayon, Panamá; Department of Microbiology and Immunology, Virginia Commonwealth University, Virginia, USA; Département Infection et Epidemiologie, Institut Pasteur, Laboratoire des Processus Infectieux à Trypanosomatidés, Paris, France

**Keywords:** Proline racemase, *Trypanosoma cruzi*, *Trypanosoma vivax*, *Trypanosoma rangeli*, Horizontal gene transfer, Gene loss, Kinetoplastid evolution, Phylogeny, Synteny, Genotyping

## Abstract

**Background:**

Proline racemase (PRAC) enzymes of *Trypanosoma cruzi* (*Tc*PRAC), the agent of Chagas disease, and *Trypanosoma vivax* (*Tv*PRAC), the agent of livestock trypanosomosis, have been implicated in the B-cells polyclonal activation contributing to immunosuppression and the evasion of host defences. The similarity to prokaryotic PRAC and the absence in *Trypanosoma brucei* and *Trypanosoma congolense* have raised many questions about the origin, evolution, and functions of trypanosome PRAC (*Try*PRAC) enzymes.

**Findings:**

We identified *Try*PRAC homologs as single copy genes per haploid genome in 12 of 15 *Trypanosoma* species, including *T. cruzi* and *T. cruzi marinkellei*, *T. dionisii, T. erneyi, T. rangeli*, *T. conorhini* and *T. lewisi,* all parasites of mammals. Polymorphisms in *Tc*PRAC genes matched *T. cruzi* genotypes: TcI-TcIV and Tcbat have unique genes, while the hybrids TcV and TcVI contain *Tc*PRACA and *Tc*PRACB from parental TcII and TcIII, respectively*.* PRAC homologs were identified in trypanosomes from anurans, snakes, crocodiles, lizards, and birds. Most trypanosomes have intact PRAC genes. *T. rangeli* possesses only pseudogenes, maybe in the process of being lost. *T. brucei, T. congolense* and their allied species*,* except the more distantly related *T. vivax*, have completely lost PRAC genes*.*

**Conclusions:**

The genealogy of *Try*PRAC homologs supports an evolutionary history congruent with the *Trypanosoma* phylogeny. This finding, together with the synteny of PRAC loci, the relationships with prokaryotic PRAC inferred by taxon-rich phylogenetic analysis, and the absence in trypanosomatids of any other genera or in bodonids or euglenids suggest that a common ancestor of *Trypanosoma* gained PRAC gene by a single and ancient horizontal gene transfer (HGT) from a Firmicutes bacterium more closely related to *Gemella* and other species of Bacilli than to *Clostridium* as previously suggested. Our broad phylogenetic study allowed investigation of *Try*PRAC evolution over long and short timescales. *Try*PRAC genes diverged to become species-specific and genotype-specific for *T. cruzi* and *T. rangeli,* with resulting genealogies congruent with those obtained using vertically inherited genes. The inventory of *Try*PRAC genes described here is the first step toward the understanding of the roles of PRAC enzymes in trypanosomes differing in life cycles, virulence, and infection and immune evasion strategies.

**Electronic supplementary material:**

The online version of this article (doi:10.1186/s13071-015-0829-y) contains supplementary material, which is available to authorized users.

## Background

The kinetoplastids (Euglenozoa: Kinetoplastea) are composed of bodonids, which include free-living and parasitic species in aquatic environments, and their descendants, the obligate parasitic trypanosomatids. These include parasites of insects and plants as well as *Trypanosoma* and *Leishmania,* which alternate between invertebrate and vertebrate hosts, including humans [1-3]. Although the trypanosomes are parasites of all vertebrate classes, they are highly divergent in their host ranges. Some species have a single vertebrate host, while others can infect diverse genera and orders. Most trypanosomes are thought to develop exclusively in the bloodstream, but a few species can also live in extra-vascular (*T. brucei* ssp. and *T. vivax*) and intracellular (*T. cruzi* and allied species) compartments. In vectors such as flies, hemipterans, mosquitoes, fleas or leeches, most trypanosomes develop in the gut, although some can invade the haemolymph and multiply and differentiate to infective forms in the salivary glands of their vectors [1,3-5].

Morphological and functional diversification has given rise to trypanosomatids differing in life cycles, vertebrate hosts, and vectors. Parasite adaptations to the variable host and vector environments have resulted in the development of diverse physiological processes and unique mechanisms to evade the host defences. Characterisation of molecules essential to metabolism and host interactions is fundamental to the elucidation of the emergence of pathogenicity and the diverse evolutionary strategies used by trypanosomes to infect and survive within a wide range of vertebrates and invertebrates.

Polyclonal lymphocyte-B activation is one of the major immunological disorders observed during microbial infections and is among the main strategies used by *T. cruzi* to evade the host specific immune response, ensuring its survival in vertebrate hosts [6-11]. This process can be triggered by proline racemase (PRAC) enzymes released by *T. cruzi*, which are implicated in the virulence of the parasite and induce indiscriminate activation of B-cells producing high levels of non-specific antibodies that contribute to immunosuppression and, consequently, to parasite immune evasion and persistence in the host. Immunological and biochemical studies confirmed that both *Tc*PRAC (*T. cruzi*) and *Tv*PRAC (*T. vivax*) exhibited proline racemase activity and B-cell mitogenicity, inducing polyclonal activation, delayed specific immune responses to parasite antigens favouring parasite immune evasion, and concomitant increase of parasitemias in the early phase of infection [7-12].

Amino-acid racemases are enzymes that catalyse the interconversion of free L- and D-amino acids. D-amino acids released by bacteria are key factors for the cell wall remodelling essential for adaptation to environmental challenges. Alanine- and glutamate-racemases are necessary for the synthesis of bacterial cell wall (peptidoglycan), which provide protection against proteolysis and host immune defences [13-15]. The enzymatic activity of Proline-racemase (PRAC) enzymes has been identified in a restricted group of bacteria, acting as a virulence factor in the highly pathogenic *Clostridium difficile* and *Pseudomonas aeruginosa* [14-16]. The first PRAC enzyme was isolated from *Clostridium sticklandii* in 1968 [15]. The first eukaryotic PRAC was reported in 2000 in *T. cruzi* [11]. In 2009, a PRAC was reported in *T. vivax* [8]. It is now recognised that the PRAC-like gene family is widely distributed throughout prokaryotes but scarce in eukaryotes, which according to phylogenetic analyses have acquired distinct bacterial PRAC-like genes by independent horizontal gene transfer (HGT) events. The repertoires and roles of PRAC-like genes in eukaryotes other than trypanosomes, including fungi, humans and other animals, are just beginning to be appreciated [16,17].

The search for PRAC genes in the genome of the *T. cruzi* CL Brener strain revealed two genes encoding two enzyme isoforms essential for viability and differentially expressed during parasite development: *Tc*PRACA (secreted by metacyclic and bloodstream trypomastigotes) and *Tc*PRACB (intracellular protein of epimastigotes). The two enzymes share 96% amino-acid identity but differ in kinetic properties relevant to catalytic activities [9,10]. Evidence provided by inhibitors of *Tc*PRAC support its suitability as a target for chemotherapy against Chagas disease [18,19]. However, to date neither the genetic nor the enzymatic diversity of *Tc*PRAC was investigated for any other strain of *T. cruzi* besides CL Brener. All protein candidates for drug design should consider the diversity within *T. cruzi* [20].

*T. cruzi* is a complex of genetically diverse isolates distributed in seven intraspecific subdivisions: the DTUs (Discrete Typing Units) TcI-TcVI and Tcbat. The heterogeneity of *T. cruzi* isolates has been implicated in different clinical forms of the disease. Chagas disease pathology ranges from subclinical infection to severe cardiac and digestive syndromes. However, attempts to associate *T. cruzi* genotypes with clinical forms, degrees and types of host-cell invaded, virulence and metacyclogenesis suggested some degrees of association, but involved several factors from hosts and parasites that are not well understood [21].

As mentioned above, *Tc*PRAC enzymes contribute to delays in the effective host immune response by non-specifically activating B-lymphocytes, thus enhancing the ability of the parasite to avoid immune clearance [8,9,11]. Treatment of macrophages with recombinant *Tc*PRAC induces the secretion of a soluble factor that promotes B-cell proliferation. *Tc*PRAC also activates the production of a cytokine known to enhance host susceptibility to *T. cruzi,* thus enhancing parasite virulence [22,23]. Over-expression of *Tc*PRAC genes increased the differentiation of non-infective epimastigotes into infective metacyclic trypomastigotes, suggesting that the enzyme may regulate intracellular metabolic pathways of L-proline internalised from the vector gut. The inhibition of *Tc*PRAC significantly reduced the invasion of cells, and the intracellular differentiation of *T. cruzi* [9,10]. L-proline is one major source of energy for *T. cruzi* not only in the vectors but also during host-cell invasion and, in addition, improves parasite protection against oxidative stress [24,25]. *Tc*PRAC may also participate in the addition of D-amino acids to peptides, generating less immunogenic parasites, and maybe providing resistance against host proteolytic mechanisms as described for bacterial cell walls [10,12-14,16,22].

To date, the only trypanosome other than *T. cruzi* in which a PRAC homolog was reported is *T. vivax.* Similar to *Tc*PRAC, *Tv*PRAC displays racemase enzymatic activity, and induces polyclonal activation (mitogenic activity) in B-cells [8]. *T. vivax* evades the host immune system due to VSG expression, multiplying extracellularly in the bloodstream, and invading and multiplying in tissue spaces and the CNS similarly to the *T. brucei* ssp. agents of Sleeping Sickness [26,27].

Throughout their evolutionary history, trypanosomes have relied on various strategies to infect their hosts, obtain energy from sources available in vectors (gut and haemolymph) and vertebrate hosts (blood, intravascular and intracellular compartments), evade host defences, and develop virulence factors that play different roles according to the trypanosome species. PRAC enzymes have been implicated in these processes in *T. cruzi* and *T. vivax* [7-12]. The discovery of PRAC enzymes in *T. cruzi* and *T. vivax*, which are species separated by large genetic distances, and the absence of homologs in the genomes of *T. brucei* and *T. congolense,* which together with *T. vivax* form the clade *T. brucei* exclusive of African pathogenic trypanosomes [3,28] and the lack in *Leishmania* spp. [8], suggest that PRAC have a complex evolutionary history in the Trypanosomatidae family.

The acquisition by HGT of a large number of foreign genes from viruses, bacteria, eukaryotes and even vertebrate hosts and vectors can change genetic and metabolic repertoires, and has played important roles in the evolution of trypanosomatids and other protistan parasites. HGT has been an important evolutionary force in the adaptation of trypanosomatids to parasitism and to specialised niches within hosts, largely contributing to amino acid and carbohydrate metabolic pathways. In addition, an increasing number of putative proteins of unknown function gained from bacterium have been identified in the *T. brucei*, *T. cruzi* and *Leishmania* spp. genomes [29-35]. Recent studies have characterised putative HGT contributing to host infection, cell invasion, virulence, and pathogenesis of trypanosomatids. It was suggested that *T. cruzi* acquired genes for the calcium mobilisation necessary for host-cell invasion via ancient HGT from *Salmonella* [36]. The analysis of the phosphatidylinositol kinase gene family revealed a novel gene of *T. cruzi*, *T. brucei*, *T. congolense*, *T. vivax,* and *Leishmania* spp. that may have been acquired from a virus through HGT [37].

To achieve a better understanding of the origin, the possible bacterial donors and the evolution of *Try*PRAC genes, we searched for *Tc*PRAC homologs in the genomes of *T. cruzi* representing the whole range of intra-specific diversity (DTUs TcI-TcVI and Tcbat), other trypanosomes of mammals (*T. c. marinkellei, T. dionisii, T. erneyi*, *T. rangeli, T. conorhini* and *T. lewisi)*, trypanosomes of snakes, crocodiles, toads, lizards and birds, trypanosomatids of several other genera, and bodonids and euglenids. Here, we describe PRAC repertoires of species and genotypes of trypanosomes, taxon-rich phylogeny of eukaryotic and prokaryotic PRAC homologs, GC contents, selection pressures on the evolution of *Try*PRAC, and genome synteny analyses. Together, the results allowed us to hypothesise about the origin, and number and timing of PRAC transference that gave rise to *Try*PRAC genes.

## Methods

### Trypanosome genomes used for searches of *Tc*PRAC homologous genes

Searches for *Tc*PRAC homologs were performed by BLAST against draft and annotated genomes of trypanosomatids freely available in TriTrypDB, geneDB and NCBI data banks. Sequences of *Tc*PRAC and *Tv*PRAC [10] were used as queries; full-length sequences and specific motifs from PRAC-like gene family were used as baits for the genome analyses of *T. cruzi* CL Brener, Silvio X10 plus other strains of *T. cruzi* (Table 1) sequenced by the Kinetoplastid Genome Sequencing and Analysis Consortium NIH/NHGRI/NIAID, *T. brucei* ssp., *T. evansi*, *T. congolense*, *T. vivax*, *T. c. marinkellei, T. grayi,* and species of *Leishmania* (Table 1). In addition, we examined the freely available genomes from *Crithidia acanthocephali, Angomonas desouzai*, *Angomonas deanei*, *Strigomonas culicis*, *Strigomonas oncopelti* and *Herpetomonas muscarum,* all generated in our laboratories, plus genomes from *Phytomonas* sp., *Crithidia fasciculata* and *Endotrypanum schaudinni* (Table 1).

**Table 1:** **Trypanosomes, other trypanosomatids and free living kinetoplastids and euglenids examined in this study, and respective sequences of** ***Tc*** **PRAC homologous genes**

**Species isolate (genotype)**	**Host species**	**Data bank assessed**	***Try*** **PRAC** **Access number**
			**Genome/GenBank**
**Trypanosomes**			
*Trypanosoma cruzi*			
Sylvio X10.6 (TcI)	*Homo sapiens*	TritrypDB &	TCSYLVIO_010607
JR cl4 (TcI)	*Homo sapiens*	Genome draft (WU) &	KP001304
G (TcI)	*Didelphis marsupialis*	Genome draft (ATOL) #	KP001302
Esmeraldo (TcII)	*Homo sapiens*	Genome draft (WU) &	KP001301
M6241 cl6 (TcIII)	*Homo sapiens*	Genome draft (WU) &	KP001305
Can III (TcIV)	*Homo sapiens*	Genome draft (WU) &	KP001298
CLBrener –Esm (TcVI)	*Triatoma infestans*	TritrypDB &	TcCLB.506795.80
CLBrener-Non-Esm (TcVI)	*Triatoma infestans*	TritrypDB &	TcCLB.509935.29
Tula cl2 (TcVI)	*Homo sapiens*	Genome draft (WU) &	KP001312
1994 (Tcbat)	*Myotis levis* (bat)	Genome draft (USP)	KP001313
*Trypanosoma cruzi marinkellei B7*	*Phyllostomus discolor* (bat)	TritrypDB &	Tc_MARK_8728
TCC344	*Carollia perspicillata* (bat)	Genome draft (ATOL) #	KP001314
*Trypanosoma dionisii* TCC211	*Eptesicus brasiliensis* (bat)	Genome draft (ATOL) #	KP001263
*Trypanosoma erneyi* TCC1946	*Mops condylurus* (bat)	Genome draft (ATOL) #	KP001315
*Trypanosoma rangeli* AM80	*Homo sapiens*	Genome draft (ATOL) #	KP001264
*Trypanosoma conorhini* TCC025	*Rattus rattus*	Genome draft (ATOL) #	KP001316
*Trypanosoma lewisi* TCC034	*Rattus rattus*	Genome draft (ATOL) #	KP001317
*Trypanosoma vivax* Y486	*Bos taurus*	TritrypDB &	TvY486_0703770
*Trypanosoma b. brucei* TREU927	*Glossina pallidipes*	TritrypDB &	
*Trypanosoma b. gambiense* DAL972	*Homo sapiens*	TritrypDB &	
*Trypanosoma congolense* IL3000	*Bos* sp.	TritrypDB &	
*Trypanosoma serpentis* TCC1052	*Pseudoboa nigra* (snake)	Genome draft (USP) #	KP001318
*Trypanosoma grayi* ANR4	*Glossina palpalis*	TritrypDB &	Tgr.146.1080
*Trypanosoma* sp. TCC339	*Rhinella marina* (toad)	Genome draft (USP) #	KP001319
*Trypanosoma* sp. TCC1825	*Ramphocelus nigrogularis* (bird)	Genome draft (USP) #	KP001320
*Trypanosoma* sp. TCC878	*Mabuya frenata* (lyzard)	Genome draft (USP) #	KP001321
**Other Trypanosomatids**			
*Crithidia fasciculata*	*Anopheles quadrimaculatus*	TritrypDB &	
*Crithidia acanthocephali*	*Acanthocephala femorata* (fly)	(ATOL) & GenBank AUXI01000000	
*Leptomonas costaricensis*	*Ricolla simillima* (Hemiptera)	(ATOL) #	
*Leishmania major* Friedlin	*Homo sapiens*	TritrypDB &	
*Leishmania tarentolae*	*Tarentola mauritanica* (lyzard)	TritrypDB &	
*Endotrypanum schaudinni*	*Choloepus hoffmani* (sloth)	Genome draft (ATOL) #	
*Angomonas desouzai*	*Ornidia obesa* (fly)	(ATOL) & GenBank AUXL01000000	
*Angomonas deanei*	*Zelus leucogrammus* (Hemiptera)	(ATOL) & GenBank AUXM01000000	
*Strigomonas culicis*	*Aedes vexans*	(ATOL) & GenBank AUXH01000000	
*Strigomonas oncopelti*	*Oncopeltus sp.* (Hemiptera)	(ATOL) & GenBank AUXK01000000	
*Herpetomonas muscarum*	*Musca domestica*	(ATOL) & GenBank AUXJ01000000	
*Phytomonas* sp.	*Jatropha macrantha* (plant)	Genome draft (ATOL) #	
**Free living euglenozoans**			
*Bodo* sp. ATCC 50149		Genome draft (ATOL) #	
*Parabodo caudatus* ATCC 30905		Genome draft (ATOL) #	
*Discoplastis spatirhyncha* SAG1224.42		Genome draft (ATOL) #	
*Eutreptia viridis* SAG 1226-1c		Genome draft (ATOL) #	

TCC: Trypanosomatid Culture Collection of the University of São Paulo, SP, Brazil.

TritrypDB (http://tritrypdb.org).

WU: Washington University (USA) - Kinetoplastid Genome Sequencing and Analysis Consortium (NIH/NHGRI/NIAID).

ATOL: Assembling the Tree of Life (NSF-USA);

USP: Department of Parasitology, University of São Paulo, USP.

& publicly available genomes; # access to these ongoing genomes can be obtained by contacting the corresponding author.

We also searched for PRAC genes in genomes that have been generated in our laboratories for a large number of euglenozoans within the ATOL (Assembling the Tree of Life, NSF-USA) and TCC-USP (Brazil) projects aiming highly comprehensive phylogenomic inferences. The following ongoing genomes were analyzed: *T. cruzi* (G and Tcbat), *T. cruzi marinkellei* (TCC344), *T. dionisii* (TCC211), *T. erneyi* (TCC1946), *T. rangeli* (AM80), *T. lewisi* (TCC34), *T. conorhini* (TCC025E), *T. serpentis* (TCC1052), *Trypanosoma* sp. of toad (TCC339), *Trypanosoma* sp. of lizard (TCC878) and *Trypanosoma* sp. of bird (TCC1825). PRAC genes were also searched in draft genomes of bodonids (*Bodo* sp. and *Parabodo caudatus*), and euglenids (*Euglena gracilis*, *Eutrepia viridis*, *Discoplastis spathirhyncha*) (Table 1). The trypanosomatids employed for genome sequencing are cryopreserved at Trypanosomatid Culture Collection of the University of São Paulo (TCC-USP). PRAC sequences retrieved from the genomes were all deposited in GenBank (Table 1).

The draft genomes generated in our laboratories were sequenced using standard pyrosequencing shotgun methodology according to Roche 454 protocols and assembled by Roche’s Newbler software (version 2.3) as previously described [32,33]. The ongoing genomes from trypanosomes of toad, snake, lizard and bird were obtained using the MiSeq Illumina plataform (mate-pair reads), and assembled using Newbler (version 2.9) as described [32]. Access to the unpublished draft and ongoing genomes analyzed in this paper can be obtained by contacting the corresponding author.

### Essential motifs and residues, alignments, and phylogenetic analyses of PRAC sequences

Predicted amino-acid sequences from PRAC genes identified in the trypanosome genomes were evaluated regarding motifs essential for racemase activity to identify putative PRAC homologous enzymes, thus ensuring the selection of genes encoding racemases, and excluding closely related PRAC-like genes such as those coding for epimerases. Previous studies on *T. cruzi* PRAC enzymes and bacterial PRACs demonstrated that catalytic cysteines (Cys130 and Cys300), active site (SPCGT) and essential motifs (MCGH and MIII) are not sufficiently stringent to discriminate between PRAC-like enzymes such as hydroxyproline-2 epimerase (HyPRE) and racemase. Therefore, the residues R1, R2, and R3, which are involved in substrate specificity, were used to distinguish between PRAC from HyPRE enzymes *in silico* [7,8,10,16]. These features were examined to select the genes encoding putative homologous PRAC enzymes in trypanosome genomes.

Amino-acid and nucleotide sequences of whole *Tc*PRAC-homologous genes (~1062 bp) from the various trypanosome species were obtained from genome data banks, aligned using Clustal X v2.0 and manually adjusted. In addition, partial (~1015 bp) PRAC nucleotide sequences obtained by PCR-sequencing were used for polymorphism analysis within *T. cruzi* by comparing sequences from TcI-TcVI and Tcbat isolates*.* An alignment was created with partial PRAC amino acid sequences from *T. rangeli* isolates of lineages A-E and *T. conorhini* as outgroup.

Maximum-likelihood (ML) and maximum-parsimony (MP) analyses were performed respectively with RAxML v7.2.8 and PAUP*v4b10 based on nucleotide and amino acid alignments. The MP tree search and bootstrap analysis were done using 500 replicates of random addition sequence swapped using TBR. The ML analysis employed GTRGAMMAI with 500 maximum parsimony starting trees. Model parameters of ML analysis were estimated over the tree search and bootstrap support was estimated with 1000 replicates in RAxML using maximum parsimony as starting trees and optimized in the best tree as previously described [38-43].

To compare the highly conserved *Tc*PRAC genes from all DTUs, a network genealogy was inferred using nucleotide sequences and the neighbor-net method with Kimura’s 2-parameter model implemented in SplitsTree4 V4.10 as described previously [28,44]. Internode support was estimated by performing 100 bootstrap replicates using the same parameters optimized for network inferences.

### PCR amplification and sequencing of PRAC gene sequences from *T. cruzi* and *T. rangeli* isolates

PCR amplification of partial *Tc*PRAC sequences (~1015 bp comprising all essential motifs and residues of *Tc*PRAC enzyme) from large number of isolates from *T. cruzi* and *T. rangeli* (Additional file 1) was conducted as previously described [8] using the primers PRAC1 (5’-CTTCCCATGGGGCAGGAAAAGCTTCTG-3’) and PRAC2 (5’-CTGAGCTCGACCAGATCTATCTGC-3’). The PCR-amplified products were cloned, and 3–5 clones from each isolate were sequenced, whereas ~10 clones were sequenced from each of the hybrid isolates*.* The PRAC sequences representing the genetic diversity within *T. cruzi* and *T. rangeli* were deposited in GenBank and the access numbers are listed in Additional file 2.

### Phylogenetic analysis based on gGAPDH gene sequences

Phylogenetic tree of Euglenozoa species based on gGAPDH gene sequences was inferred by ML and MP as described above for PRAC genes. The alignment created for this analysis was done using for guidance a comprehensive alignment of kinetoplastid gGAPDH genes [3] and included sequences from 32 trypanosome species, non-trypanosome trypanosomatids of seven genera, and five bodonids and euglenids as outgroups. Bootstrap support was estimated with 100 pseudoreplicates in RAxML using GTRGAMMA. The Genbank access numbers of all gGAPDH genes included in the phylogenetic trees are listed in Additional file 1.

### Trypanosomes lacking PRAC genes as determined by genome search and/or PCR amplification

The absence of PRAC genes in the genomes of *T. brucei* ssp*.* and *T. evansi* was confirmed by negative results in PCR tests of additional isolates of each species. Besides the lack of PRAC homologs in the genome of *T. congolense* IL3000 (subgroup Savannah), results were also negative for all other members of *Nannomonas* tested: *T. congolense* Cam22 (Forest), WG5 (Kilifi) and TREU1475 (Savannah), *T. simiae* and *T. godfreyi* [28]. DNA samples from these trypanosomes were kindly provided by Wendy Gibson, Bristol University, UK.

### Horizontal-gene-transfer analysis

The horizontal-gene-transfer (HGT) analysis includes a comprehensive dataset of 2,530 PRAC-like protein sequences from prokaryotes and eukaryotes in the non redundant (NR) database. A BLASTp search was performed with a maximum-expected-value threshold of 1e-20, using the *Tc*PRACA and *Tc*PRACB sequences as queries. The retrieved sequences were checked for PRAC-like domains using the Batch search tool in the Conserved Domain Database (http://www.ncbi.nlm.nih.gov/Structure/bwrpsb/bwrpsb.cgi). Multiple-sequence alignment was performed using MUSCLE v3.8, and edited using Gblocks v0.91b [45] to eliminate poorly aligned positions. The final phylogenetic tree was obtained by ML analysis with 2,530 sequences under the WAG substitution model with gamma-distributed heterogeneity rate categories, and estimated empirical residue frequencies (model PROTGAMMAWAG) as implemented in RAxMLv7.2.8. One hundred different best tree searches were performed, and the tree with best likelihood found was kept. RAxML rapid bootstrap was performed with 100 pseudoreplicates. The tree was also visualized using Dendroscope v3.2.4 [46] with further cosmetic adjustments done using the Inkscape vector image editor (http://inkscape.org). To better resolve and visualize the putative HGT donor lineages, a ML analysis was performed using a subset of 303 PRAC-like sequences from NR database and 39 *Try*PRAC aligned with the nearest neighbor taxons identified in the analysis using 2,530 sequences. The tree search and bootstrap were conducted using the same parameters for both datasets.

### Genomic organization, GC content and codon pressure analyses of trypanosome PRAC genes

The comparison of PRAC genomic organization in the analyzed genomes was performed with the bl2seq BLASTX algorithm using the flanking downstream and upstream regions (~10,000 bp) previously reported for *T. cruzi* and *T. vivax* [8,9] in all trypanosome genomes investigated in this study. Codon-selection analysis was performed using the HyPhy v2.2 package [47] with a threshold *p*-value < 0.05. The GC content comparison between *Try*PRAC homologs and both flanking genes and whole genomes were conducted using the mfsizes v. 1.8.3 software (http://sourceforge.net/projects/mfsizes/).

## Results and discussion

### Analysis of kinetoplastid and euglenid genomes shows *Tc*PRAC homologs exclusively in *Trypanosoma*

**Figure 1:**
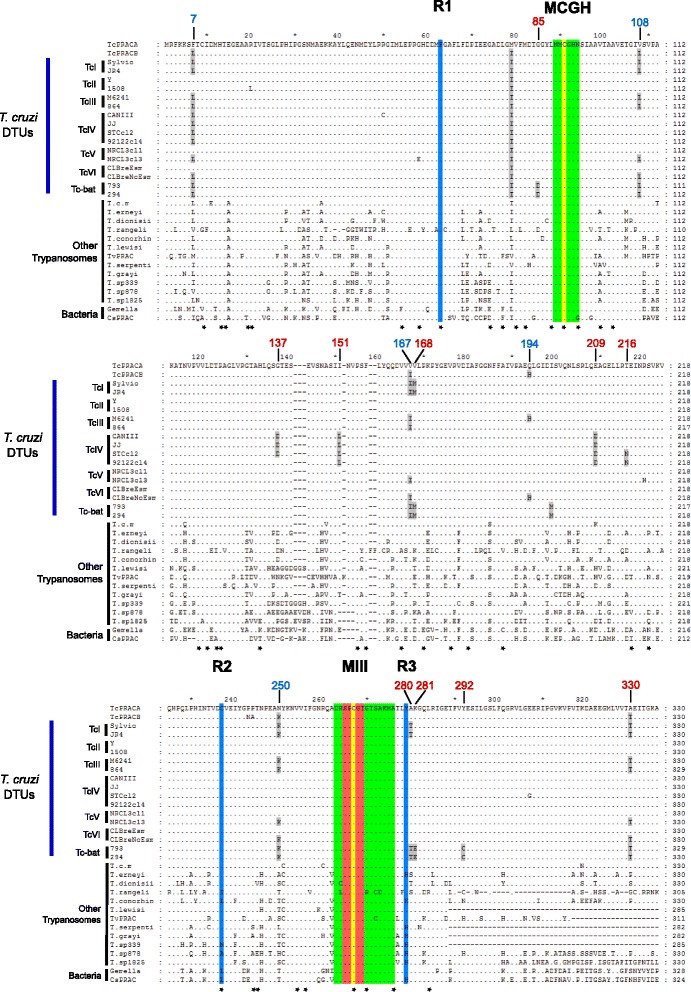
Alignment of predicted amino acid sequences of proline racemase (PRAC) homologous genes from *T. cruzi* (DTUs TcI-TcVI and Tcbat), *T. cruzi marinkellei* (*T. c. m*), *T. erneyi*, *T. dionisii*, *T. rangeli*, *T. conorhini*, *T. lewisi*, *Tv*PRAC - *T. vivax*, *T. serpentis*, *T. grayi*, *T. sp*. from toad (TCC339), *T.* sp. from lizard (TCC878), and *T.* sp. from bird (TCC1825) and PRAC from *Gemella haemolysans and Clostridium difficile* (CsPRAC). Essential motifs (MCGH and MIII) are in green, and the active site (SPCGT) in red. R1, R2 and R3 are residues involved in substrate specificity. Cys91 and Cys267 are the catalytic cysteines. Blue numbers indicate differences between *Tc*PRACA and *Tc*PRACB, and red numbers indicate substitutions found in newly identified *Try*PRAC homologs. Black stars indicate negatively selected amino acid residues.

We searched for *Tc*PRAC homologs in the genomes of trypanosomatids, bodonids and euglenids using *Tc*PRACA and *Tc*PRACB sequences as queries. Homologs were identified in *T. cruzi, T. c. marinkellei*, *T. dionisii*, *T. erneyi*, *T. rangeli*, *T. conorhini* and *T. lewisi*. In addition to these mammalian parasites, *Tc*PRAC homologs were found in trypanosomes from snake (*T. serpentis*) [42], crocodile [43,48], lizard (TCC878), bird (TCC1825) and toad (TCC339) [41] (Table 1, Figure 1). All these species exhibit a single copy of a *Try*PRAC homolog per haploid genome, and no other PRAC-like gene was identified in the kinetoplastid genomes.

Previous studies reported the absence of PRAC homologs in the genomes of *T. b. brucei* and *T. b. gambiense,* while only a remnant of a PRAC gene was suggested for *T. congolense* [8-10]. Thus, it was not surprising that *T. evansi*, which is highly closely related to *T. brucei* ssp. and *T. equiperdum*, all forming the subgenus *Trypanozoon*, also lacked PRAC. In addition, attempts to detect even small fragments of PRAC genes by PCR amplification failed for all investigated species of the subgenus *Nannomonas*: *T. congolense* of subgroups Forest, Kilifi and Savannah, *T. simiae* and *T. godfreyi.* We were also unable to detect any PRAC fragment in the recently assembled genome of *T. congolense* (Savannah). In the phylogenetic trees, the species of *Trypanozoon* and *Nannomonas* formed a monophyletic assemblage within the main *T. brucei* clade in which *T. vivax* has a basal position [3].

**Figure 2:**
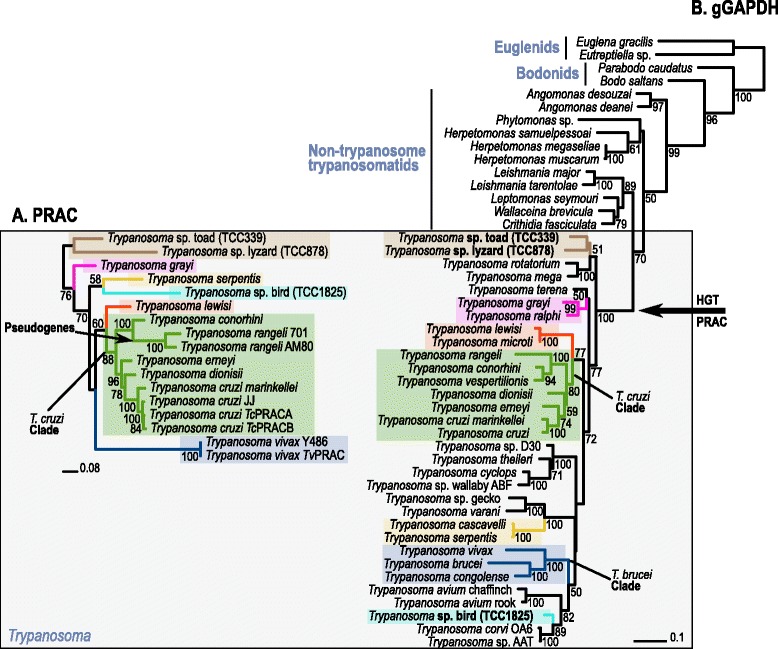
Congruent phylogenies of trypanosome species inferred using sequences from PRAC (A) and gGAPDH (B) genes. Maximum likelihood phylogenetic trees were inferred using entire *Try*PRAC nucleotide sequences from 13 trypanosome species (−Ln = 8403.576084), and gGAPDH sequences from 32 trypanosome species, non-trypanosome trypanosomatids of seven genera, and bodonids and euglenids as outgroups (−Ln = 13563.555163). Numbers on branches represent bootstrap support (>50) estimated with 100 pseudoreplicates in RAxML using GTRGAMMA. The arrow on the PRAC genealogy (**A**) indicates pseudogenes found in *T. rangeli*. The arrows on the gGAPDH tree (**B**) indicate the places hypothesised for the horizontal transference of bacterial PRAC gene to a common ancestor of *Trypanosoma,* and the gene loss in a common ancestor of *T. brucei* ssp. and *T. congolense*. Genbank accession numbers of PRAC and gGAPDH gene sequences are respectively showed in Table 1 and Additional file 1.

Examination of non-trypanosome Trypanosomatidae species revealed that PRAC genes, and even other genes of the PRAC-like family, are absent not only from the *L. major* genome as previously reported [8-10,31], but also from other *Leishmania* species. Moreover, the genomes of monoxenous parasites of insects of the genera *Crithidia, Leptomonas*, *Angomonas*, *Strigomonas* and *Herpetomonas* and the plant parasites of the genus *Phytomonas* all lacked PRAC. Regarding other kinetoplastids, our searches did not reveal any putative PRAC-like genes in *Bodo* sp. and *Parabodo caudatus.* In addition, we did not identify PRAC-like genes in the genomes of the basal species within Euglenozoa: *Euglena gracilis*, *Eutrepia viridis* and *Discoplastis spathirhyncha* (Figure 2B).

In conclusion, PRAC homologs are widespread in trypanosomes but absent from the genomes of non-trypanosome trypanosomatids of all genera investigated, and in the bodonids and euglenids to date examined. PRAC homologs were identified in species of the main clades of the *Trypanosoma* phylogenetic tree, including the basal Aquatic clade [3,41], but in the *T. brucei* clade only *T. vivax* carries a homologous PRAC gene. Results strongly suggest that a prokaryotic PRAC was introduced at the root of *Trypanosoma* and fixed in the genome of a common trypanosome ancestor.

### Molecular characterisation of new putative PRAC-like enzymes of trypanosomes

The catalytic mechanism of *Tc*PRAC is essentially identical to that of the prokaryotic PRAC enzymes. The activity of PRAC enzymes depends mainly on two cysteine residues that transfer protons to the chiral carbon (C^α^) of L-proline/D-proline enantiomers, resulting in the stereoinversion of its configuration [7,10,16]. PRAC-like genes include diverse racemase-like genes that exhibit strong sequence similarity to proline racemases. A few eukaryotic PRAC-like enzymes have all the residues critical for racemase activity. Most of these enzymes function as proline epimerases, which are common in prokaryotes or as proline dehydratases as reported in humans [16,17].

Alignment of *Try*PRAC homologs from 13 trypanosome species, using *Tc*PRACs and prokaryotic PRAC sequences for guidance, revealed the two cysteine residues, the active site SPCGT, and the MCGH motif in all sequences. Only the MIII motif had relevant polymorphism. At the residues RI, R2, and R3, which are involved in substrate specificity, all *Try*PRAC homologs have a conserved R1 but a variable R2 and R3. All species of the subgenus *Schizotrypanum* (*T. cruzi, T. c. marinkellei*, *T. dionisii* and *T. erneyi*) share identical motifs, and highly conserved essential residues. Within this clade, only *T. erneyi* showed one non-synonymous substitution at R3. In contrast, more distantly related trypanosomes showed synonymous and non-synonymous substitutions at R2 (*T. rangeli, T. conorhini*, *T.* sp. from toad and *T.* sp. from lizard) and R3 (*T. conorhini*, *T. serpentis, T. grayi* and *T.* sp. from toad) (Figure 1). The implications of these substitutions in substrate specificity merit further investigation.

All putative *Try*PRAC sequences lacked a signal peptide, suggesting that the encoded enzymes are intracellular, and can be released through the flagellar pocket and/or parasite death [8,10,18]. Despite highly conserved catalytic domains, we identified at least one novel PRAC homolog for each species of trypanosome. *In silico* analysis suggested that most trypanosomes can express PRAC proteins with racemase activities (Figure 1). Homologous PRAC of *T. vivax* differed in several residues when compared to those from *T. cruzi*. Although the PRAC genes from trypanosomes of non-mammalian hosts such as snakes (*T. serpentis*), crocodiles (*T. grayi*), toads, lizards and birds differed in several residues when compared to homologs of *Schizotrypanum* species, all sequences could be aligned with confidence with both *Tc*PRAC and prokaryotic PRAC genes (Figure 1).

### Phylogenetic relationships of PRAC homologs from 13 trypanosome species agree with the currently recognised phylogeny of *Trypanosoma*

*T. cruzi* is highly closely related to all other species of the subgenus *Schizotrypanum* (*T. c. marinkellei*, *T. dionisii* and *T. erneyi*)*,* which are all called *T. cruzi*-like because they share morphology of blood and culture forms, although they differ in hosts, vectors and pathogenicity. Development as amastigotes and differentiation into trypomastigotes within mammalian cells *in vitro* is a unique feature of *Schizotrypanum*, whereas *in vivo,* only *T. cruzi* infects mammals other than bats. Nevertheless, as in *T. cruzi* infection, nests of amastigotes in cardiac cells can be found in bats infected with *T. cruzi*-like species. *T. cruzi* is transmitted by triatomines, while cimicids are vectors of *T. dionisii.* These trypanosomes share development restricted to the vector guts [5,38-40,44].

For phylogenetic inferences within *Schizotrypanum,* we compared isolates of all species mentioned above. According to strongly supported branching patterns on both PRAC (Figure 2A) and gGAPDH (Figure 2B) phylogenetic trees, all species clustered tightly, forming a monophyletic assemblage of trypanosomes. The relationships among the *Schizotrypanum* species and the DTUs of *T. cruzi* were inferred using entire PRAC amino acid sequences. Results corroborated the clustering of sequences according to species (Additional file 3). In agreement with previous analysis of several other genes [49] such as gGAPDH and cathepsin L-like genes, *Try*PRAC genealogy and nucleotide sequence divergences confirmed *T. c. marinkellei* as the closest relative of *T. cruzi* (~7.5 of *Try*PRAC sequence divergence between the two species). This species was followed by *T. erneyi* from African bats (~14%) and *T. dionisii* (14.5%) from Old World bats. Large genetic distances separated *T. cruzi* and *T. rangeli* (~30% PRAC sequence divergence), and *T. cruzi* and *T. vivax* (~38%) PRAC sequences. Compared to divergence among *Try*PRAC sequences, the species of the subgenus *Schizotrypanum* were separated by much smaller gGAPDH sequence divergences (~8.0% between *T. cruzi* and *T. dionisii*), whereas ~15% and ~17.5% of gGAPDH sequence divergence separated *T. cruzi* from *T. rangeli* and *T. vivax*, respectively. Divergences of gGAPDH and the more conserved SSU rRNA genes, which are the traditional genes employed for phylogenetic inferences of the Trypanosomatidae, were previously reported for the trypanosome species included in PRAC phylogeny [5,39,40].

In the bat-seeding hypothesis for the origin of the *T. cruzi* clade [39], a scenario has been proposed in which ancestral trypanosomes of bats evolved exclusively in Chiroptera, giving rise to the bat-restricted species [38-40], or evolved through multiple independent host jumps, giving rise to species infecting other mammals (such as rats, civets and monkeys) in the Old World, and to the generalists *T. cruzi* and *T. rangeli,* which are species infective to bats plus a broad range of other mammals, including human and non-human primates in the New World [3,5,38-40,50-53]. Also in agreement with previous studies, in the PRAC genealogy *T. rangeli* and *T. conorhini* formed the sister group of *Schizotrypanum,* together constituting the clade *T. cruzi* that also harbours other trypanosomes, mostly from bats [39,40]. *T. lewisi,* the basal species of the clade *T. cruzi*, is a non-pathogenic and cosmopolitan trypanosome of domestic rats transmitted by fleas, which can opportunistically infect immune-compromised human and non-human primates [54] (Figures 2A, 2B).

The toad and lizard trypanosomes included in PRAC genealogy represented the basal branches of the *Trypanosoma* gGAPDH phylogenetic trees showing PRAC genes highly divergent from *Tc*PRAC (Figure 2A). Previous [3,41-43,48] and herein inferred phylogenies based on gGAPDH genes (Figure 2B) demonstrated that anuran trypanosomes nested into the so-called “Aquatic clade”, which also includes trypanosomes transmitted by aquatic leeches of fishes, turtles and platypus, besides a lizard trypanosome of unknown vector. The aquatic clade was strongly supported as the most basal of *Trypanosoma* [3]. Also concordant with previous phylogenies, PRAC genes of trypanosomes from snakes, birds and crocodiles, which are transmitted by insects, all clustered into the “terrestrial” clade that also includes PRAC sequences from all trypanosomes of mammals (Figure 2A) in agreement with previous SSU rRNA and gGAPDH phylogenies [3,41-43,48].

### Repertoires and phylogenetic relationships among *T. cruzi* PRAC homologs of all DTUs and Tcbat

Comparison of whole *Try*PRAC amino acid sequences revealed relevant polymorphisms (~3.0% sequence divergence) within *T. cruzi*. Aiming an intra-specific analysis of *Tc*PRACs, we compared entire amino acid sequences from *T. cruzi* Sylvio X10.6, JRcl4 and G (TcI), Esmeraldo cl3 (TcII), M6241cl6 (TcIII), CANIII (TcIV), CL Brener (TcVI), Tula (TcVI), and Tcbat. Unlike all other isolates, which carried a single PRAC gene, *T. cruzi* CL Brener exhibited *Tc*PRACA and *Tc*PRACB [10], found in this work in the Esmeraldo-like and non-Esmeraldo-like haplotypes, respectively.

We evaluated all signatures defined for *Tc*PRAC activity, and polymorphisms used to differentiate between *Tc*PRACA and *Tc*PRACB. A leucine at position seven (typical of *Tc*PRACB) was found in TcI, TcIII, TcIV and Tcbat. The phenylalanine at this position that had been reported to be specific to *Tc*PRACA was found in Y and TCC1508 (TcII), but not in Esmeraldo (TcII). *Tc*PRACs from all DTUs have isoleucine at position 79 (like *Tc*PRACB), while methionine at this position in *Tc*PRACA was exclusive to CL Brener. Like *Tc*PRACA, TcII and TcIV had valine at positions 108 and 167 and asparagine at position 250. At these positions, TcI, TcIII and Tcbat had leucine, isoleucine and lysine, respectively, like *Tc*PRACB. Like the hybrid CL Brener (TcVI), both *Tc*PRACA and *Tc*PRACB were identified in the hybrid NRCL3 (TcV). New polymorphic residues evidenced novel *Tc*PRAC homologs defining TcI-TcIV-specific profiles while TcV and TcVI can be identified by the presence of both *Tc*PRACA and *Tc*PRACB. Polymorphic amino acids defining each *T. cruzi* DTU are showed in Additional file 3.

**Figure 3:**
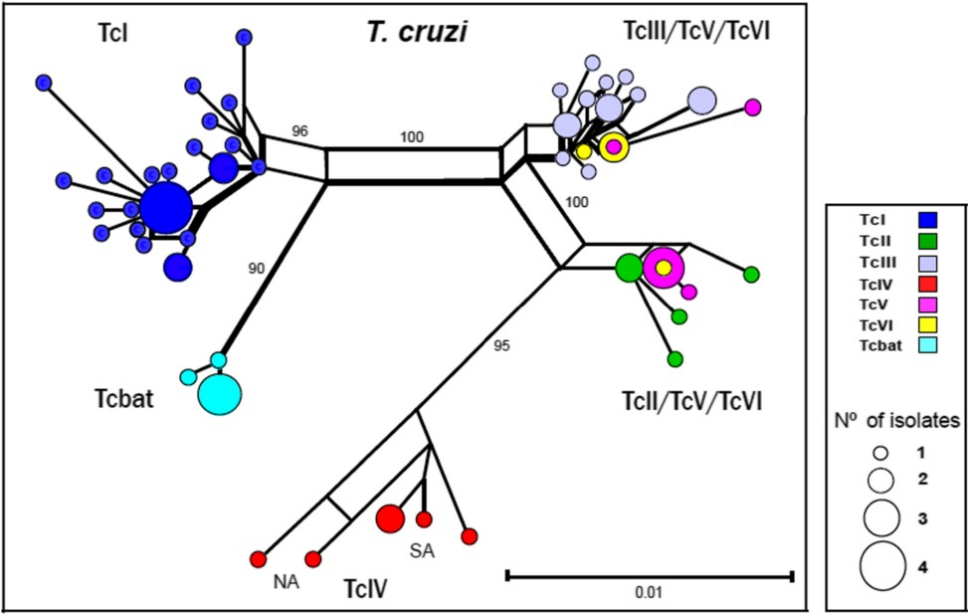
Network genealogy of partial PRAC nucleotide sequences from T. cruzi isolates showing the clustering of sequences according to DTUs, and sequences from the hybrids TcV and TcV positioned within both TcII and TcIII clades. The network branching pattern indicated high homogeneity of PRAC genes from isolates of Tcbat, TcII and TcIII contrasting with the large heterogeneity in TcI and TcIV isolates. Each colour represents one DTU, and the size of the circles indicates the number of isolates in the network. Genbank accession numbers of *Tc*PRAC genes are listed in Additional file 2, and the DTU-specific profiles of polymorphic sites is showed in Additional file 3.

Due to the high sequence conservation throughout the *Tc*PRAC genes from *T. cruzi* of some DTUs, phylogenetic analyses based on amino acid sequences were unable to clearly resolve the closely related DTUs (Additional file 3 shows the network of *Tc*PRAC amino acid sequences). To assess the relationships within *T. cruzi* using the conserved *Tc*PRAC genes (63 polymorphic sites), we constructed a network using partial nucleotide sequences obtained by PCR-sequencing from 68 isolates previously genotyped [21,44,49]. The network clearly evidenced subclades corresponding to each TcI, Tcbat, TcII, TcIII, and TcIV DTUs. Sequences from TcV and TcVI clustered with TcII or TcIII, in agreement with their hybrid origin, forming a reticulate pattern in the network. The network confirmed TcI closest to Tcbat and TcII more related to TcIV and, in addition, corroborated the heterogeneity intra-DTUs TcI, TcIII, and TcIV (Figure 3).

Most previous phylogenetic analyses within *T. cruzi* lacked isolates of all DTUs (especially TcIV and Tcbat) and/or *T. cruzi*-like outgroups and, then, were insufficient to resolve intra-*T. cruzi* phylogenetic relationships. Here, *Tc*PRAC genealogy using *T. cruzi*-like species agreed with the relationships among the DTUs (including Tcbat) previously inferred using cruzipain, SSU rRNA, and cytochrome b sequences [44,49]. Diverse genes have been employed as markers for *T. cruzi* genotyping [21]*.* We demonstrated that polymorphisms of *Tc*PRAC sequences allow the genotyping of all DTUs including hybrid genotypes, and are also valuable to infer inter-DTU relationships. To our knowledge, *Tc*PRAC is the first horizontally transferred gene (non-mitochondrial) characterized with these purposes.

Taking into account that *Tc*PRAC-A and TcPRAC-B participate in *T. cruzi* development in vertebrates and vectors and have been incriminated as host defence factors [9-12,19,22,55], it is tempting to speculate whether DTU-specific *Tc*PRAC enzymes can contribute to differential degrees of metacyclogenesis, parasitemias and virulence. *T. cruzi* of different DTUs interacts differently with the host, induces distinct immune responses and infections ranging from highly lethal to virtually asymptomatic, contributing to variable clinical forms of Chagas disease. Strains of TcI, the most widespread DTU in Latin American sylvatic cycles, exhibit high levels of metacyclogenesis. TcI is responsible for human outbreaks of oral infection and severe cardiomyopathies in Central America and Northern South America. Isolates of TcI are highly diverse genetically and in terms of virulence to mice. Although some isolates induced very low parasitemia and no mortality, it was suggested that Col strain (TcI) can evade the host immune response remaining unnoticed by mononuclear cells allowing rapid multiplication during acute infection [21,50-53,56-60]. Future studies are required to evaluate the roles played by TcI-specific *Tc*PRAC in low and highly virulent strains. TcII (PRAC-A), TcV and TcVI (PRAC-A and PRAC-B) are virulent to mice, induce high parasitemias and mortality, and have been associated with both cardiac and digestive forms in humans in Southern Cone countries [21,61]. TcIII strains (PRAC-B), found in Brazil and neighbouring countries, can induce important parasitemia and pathology in mice [57,62]. TcIV (unique *Tc*PRAC) is sylvatic, orally infects humans in Brazil and Venezuela, and induces low or moderate parasitemia and mortality in mice [21,53,60]. Tcbat, found in South and Central American bats and, apparently, able to infect humans [63], is not virulent to mice inducing extremely low parasitemias and no mortality, and is unable to develop in the commonest triatomine vectors of TcI-TcVI [50].

### *Trypanosoma rangeli* pseudogenes diverged to be lineage-specific and more closely related to *T. conorhini* than to *T. cruzi* homologous PRAC

*T. rangeli* is a non-pathogenic parasite of humans and domestic and wild animals in Central and South America. This species is thought to be restricted to the bloodstream and survives host defences for months or years by unknown mechanisms. *T. cruzi* and *T. rangeli* are the only agents of human trypanosomosis in the Americas, sharing mammalian hosts and vectors in overlapping areas. *T. rangeli* overcomes the defences of the vector (*Rhodnius* spp.), multiplying in the gut and invading the haemolymph, where the parasites multiply outside and inside of haemocytes before reaching the salivary glands where metacyclogenesis takes place. This species differs from *T. cruzi*, which develops exclusively in the triatomine gut, and from *T. brucei*, which reaches the salivary glands of the vector (tsetse flies) from the proboscid [4,64,65].

Closer phylogenetic relationships of *T. rangeli* to *T. cruzi* than to *T. brucei* were strongly supported by comprehensive phylogenetic analysis based on diverse genes [3,5,38-40]. In addition, phylogenies based on PRAC (Figure 2A), gGAPDH (Figure 2B) and SSU rRNA genes have supporting *T. rangeli* more closely related to *T. conorhini* than to *T. cruzi* [3,38,40]*. T. conorhini* is a tropicopolitan species common in rats and transmitted by the also tropicopolitan *Triatoma rubrofasciata* [66,67]*.* This species shares features with both *T. cruzi* (development restricted to the gut of its triatomine vector) and *T. rangeli* (lack of both intracellular stages and pathogenicity to vertebrates). We are currently comparing the genomes of *T. conorhini, T. rangeli* and *T. cruzi* to better understand their relationships.

In contrast to predicted PRAC proteins in most trypanosomes including *T. conorhini*, which are compatible with the expression of racemases, all *T. rangeli* PRAC sequences were found disrupted by internal stop codons resulting in pseudogenes. This finding was confirmed in the PRAC found in the genome of *T. rangeli* AM80 (human isolate of basal lineage TrB from the Amazon region) and sequences from several isolates of all lineages determined by PCR-sequencing. Additional file 4 shows the alignment of *T. rangeli* PRAC pseudogenes.

**Figure 4:**
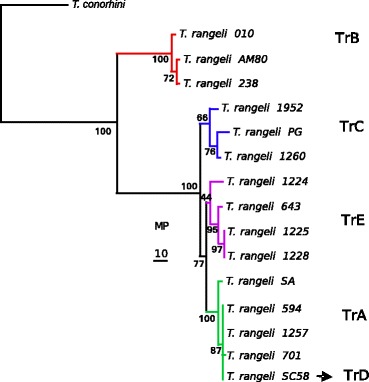
Phylogenetic inferences (MP) based on PRAC pseudogenes from 17 T. rangeli isolates using T. conorhini as outgroup. The branching pattern showing clades corresponding to the phylogenetic lineages TrA-TrE indicates a close phylogenetic relationship among the lineages TrA, C, D, E with TrB from Amazonia as the basal lineage of *T. rangeli*. Genbank accession numbers of PRAC genes included in this analysis are in Additional file 2. The alignment of *T. rangeli* pseudogenes is showed in Additional file 4.

We compared *T. rangeli* PRAC pseudogenes from 17 isolates of lineages TrA-TrE, all previously genotyped using other markers [49,50]. Previous phylogeographical studies suggest evolution within *T. rangeli* shaped by the coexistence of parasites with sympatric species of *Rhodnius* [4,51,52,64,65]. In this and previous studies based on SL, SSU rRNA, ITS rDNA, gGAPDH, and CATL sequences, TrB was always placed as the basal lineage of *T. rangeli*, whereas the relationships among the closely related TrA, C, D and E were far from resolved (Figure 4). In addition, an increasing genetic diversity within *T. rangeli* has recently been revealed [51,52,63,64]. Phylogenetic studies of all lineages using multilocus approaches are essential to better resolve the complex relationships among the lineages and to hypothesise about the evolutionary history of *T. rangeli*.

### Phylogeny and pattern of presence/absence of PRAC homologs support a single HGT from a bacterium to an ancestor of *Trypanosoma*

The identification of bacterial PRAC homologs in *T. cruzi* and *T. vivax* and their absence from *T. brucei* and *T. congolense* [7-11] suggested a complex evolution of PRAC genes in trypanosomes. Here, a broad taxon sampling comprising 15 trypanosome species, trypanosomatids of 9 additional genera and five free-living ancestors of bodonids and euglenids provides relevant insights into this process at long and short timescales. The relative timing of the HGT event was investigated by searching the presence/absence of PRAC-like genes in the increased and broad taxon sampling. No PRAC-like genes were found in trypanosomes besides those encoding putative proline racemase enzymes, such as highly similar genes coding for epimerase and dehydratase found in prokaryotes and other eukaryotes such as fungi and metazoans. Although PRAC-like genes detected in fungi and trypanosomes were all likely of prokaryotic origin, previous phylogenetic analyses revealed a polyphyletic pattern, indicating that they originated from different bacterial sources through independent HGT events [17,18]. Here, no PRAC-like genes were detected in the genomes of euglenids, bodonids or non-trypanosome trypanosomatids.

**Figure 5:**
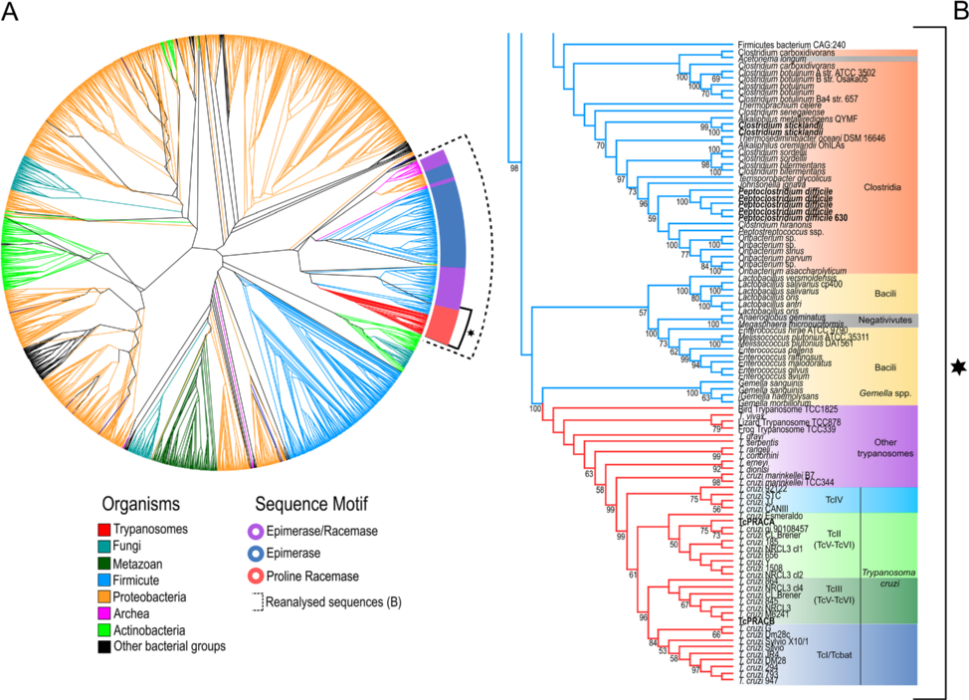
Phylogenetic analyses of PRAC-like protein sequences of prokaryotes and eukaryotes, from the NR NCBI database, and Try PRAC sequences. (A), ML analysis including 2,530 PRAC-like sequences with the best substitution model WAG + G. Bootstrap supports (100 pseudoreplicates) are displayed at nodes (only support of 50 or greater shown) in the phylogenetic tree, corresponding rectangular phylogram is showed in Additional file 5. The dotted line in (**A**) indicates the branch submitted to the reanalysis shown in Figure 5B. (**B**) ML inference restricted to *Try*PRAC (39 trypanosome species/isolates) plus 303 bacterial genes nearest relatives to *Try*PRAC homologs showing, closest to *Try*PRAC sequences, Firmicutes PRAC genes from species of *Gemella,* other bacteria of Bacilli class, and species of Clostridia class. The PRAC-like gene sequences employed in the phylogenetic analysis showed in Figures 5A and 5B are listed in Additional files 6 and 7, respectively.

After identifying in our taxon-rich phylogenetic analysis of 2,530 eukaryotic (including trypanosome) and prokaryotic PRAC-like genes (Figure 5A) the general vicinity of *Try*PRAC sequences (the names and grouping of all organisms can be found in the rectangular phylogram in Additional file 5), our targeted analysis of 342 genes showed all *Tr*yPRAC homologs in a strongly supported clade exclusive of trypanosome sequences, evidencing their common ancestry (Figure 5B). The complete list of putative donor lineages currently available on NCBI NR protein database included in the phylogenetic analysis is presented in Additional file 6.

Considering the presence/absence of PRAC genes and the congruence of all species in phylogenies based on PRAC and gGAPDH genes (Figure 2), we hypothesised gains and losses of PRAC genes during trypanosome evolution. In the most parsimonious evolutionary scenario, one prokaryotic PRAC gene was transferred through a single HGT to the root of the genus *Trypanosoma* in a common ancestor of this genus, which gave origin to all extant trypanosome species with respective *Try*PRAC homologs. The absence in *T. brucei* and *T. congolense* suggested the loss of PRAC gene by a common ancestor of the subclade comprising these species (Figure 2B). In an unlikely, less parsimonious scenario, PRAC homologs were present in all trypanosomatids or even in more basal euglenozoans, and were lost by most lineages being retained exclusively in the genus *Trypanosoma.*

Together, phylogenetic analysis and the pattern of absence/presence of PRAC-like genes strongly support our HGT hypothesis. However, it is important to consider other alternative scenarios, such as insufficient taxon sampling (donor lineages not represented in the analysis) and convergent evolution, which is an important cause of misidentified orthologs [68,69]. However, convergent evolution is in general restricted to functional domains and not across the protein length and, then, was not suggested by the alignment of *Try*PRAC with PRAC homologs from *Gemella haemolysans* and *Clostridum difficile* (Figure 1).

### Taxonomy of *Try*PRAC donor prokaryotic lineages

In the most robust approach to assess taxonomic affiliation of putative HGT donors, all the most likely candidates selected by BLAST searches (useful for a primary screen of potential donors) should be submitted to deep phylogenetic analyses [17,18,31-35,68-71]. Here, the results obtained using this approach corroborate previous phylogenetic studies of prokaryotic and eukaryotic PRAC-like genes, suggesting prokaryotic donors for *Tc*PRAC and *Tv*PRAC [8-10]. In addition, we provided new insights into the origin and evolutionary history of *Try*PRAC homologs. The finding of all *Try*PRAC homologs clustering tightly together in a monophyletic assemblage, distant from any PRAC-like genes of other eukaryotes and within a large clade of prokaryotic PRAC-like genes, corroborated a single bacterium as donor lineage (Figures 5A and 5B) [Additional file 5].

To better resolve the phylogenetic relationships and visualise the most likely *Try*PRAC donor lineages, 303 prokaryotic sequences adjacent to *Try*PRACs were employed for further ML analysis (Figure 5B). The full list of taxa included in this analysis is shown in Additional file 7. The results suggest that the donor was a bacterium related to species of the Bacilli class of the orders Lactobacillales (bacteria that live in soil, water, plants and animals) and Negativicutes (anaerobes that live in rivers, lakes and animal guts) [72], including species of the genera *Gemella, Enterococcus*, *Lactobacillus* and *Melissococus* (Figure 5B). After these species, PRAC homologs from species of *Clostridium*, *Peptoclostridium* and *Oribacterium* of the Clostridia class were the most closely related to *Try*PRAC genes. In previous studies [8,10], the closest relatives of *Tc*PRAC were *Clostridium difficile* (reclassified as *Peptoclostridium*) and *Clostridium sticklandii*, both Firmicutes of the Clostridia class [72]. In this analysis, PRAC homologs from *Gemella haemolysans*, *G. morbillorum* and *G. sanguinis,* all exhibiting typical residues of PRAC racemase enzymes, were the nearest relatives to *Try*PRACs (~57% of identity) (Figure 5B). The species of *Gemella* are oral and gastrointestinal commensals of animals including humans that, as opportunistic pathogens, cause severe pulmonary, cardiac and cerebral infections [73]. Interestingly, *G. haemolysans* and *G. morbillorum* are highly prevalent among the bacterial fauna of haematophagous dipterans of Culicidae [74], which can transmit trypanosomes among anurans [41].

### Synteny analysis revealed highly conserved gene order around *Try*PRAC homologs

**Figure 6:**
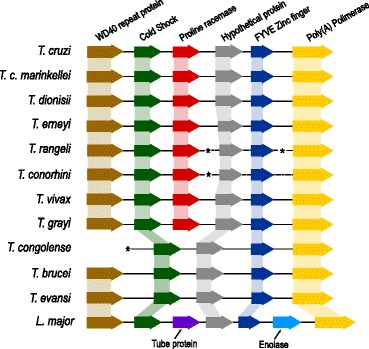
Synteny analysis showing the genomic organisation of orthologous genes arranged on Try PRAC loci in the genomes of several trypanosome species. The aligned genome segments showed total synteny in all trypanosomes exhibiting PRAC genes and the loss exclusively of PRAC gene in the corresponding *T. congolense*, *T. b. brucei* and *T. evansi* loci. Only partial synteny was observed in the *L. major* locus, with an additional putative enolase gene and the replacement of PRAC by a Tubby family gene*.*

Our findings demonstrated that *Try*PRAC homologs are ubiquitous in the genus *Trypanosoma* (Figure 2A). Previous studies revealed high conservation of gene segments containing the PRAC locus from *T. cruzi* and *T. vivax* [8,10]*.* To verify the genome organisation of PRAC genes in the different trypanosome species, we performed BLAST searches in genome databases for orthologous genes in PRAC loci. The results showed a syntenic block shared by all species (Figure 6). The adjacent regions of PRAC genes exhibited high synteny in *T. cruzi*, *T. c. marinkellei, T. erneyi*, *T. dionisii, T. vivax* and *T. grayi,* with at least 8 genes arranged in the same order: K39 kinesin, WD domain-containing protein, cold shock domain-containing protein, PRAC, hypothetical protein, zinc-finger protein, poly (A) polymerase, carbohydrate kinase and phosphatidyl serine. Syntenic organisation strongly supports orthology, with a single insertion of a prokaryotic PRAC gene between the cold-shock and hypothetical protein genes. Although *T. congolense* and *T. b. brucei* lack PRAC genes, the synteny of this genome segment was retained in these species as well as in this locus of the *T. evansi* genome. These species nested into a single clade with *T. vivax* as the basal species. The synteny in PRAC loci corroborated the loss of PRAC by a common ancestor of these species after the divergence of *T. vivax* (Figure 2B). Figure 6 shows the conserved gene order of five orthologs flanking the gene PRAC of trypanosomes.

Synteny analysis requires larger contigs still not available in some ongoing genome drafts. Consequently, synteny was confirmed partially for *T. rangeli* and *T. conorhini* (Figure 6). Only the two upstream genes were found in *T. serpentis,* and no genes flanking PRAC genes could be confidently located in the genomes of the non-mammalian trypanosomes. The PRAC locus showed only partially syntenic orthologs in the available genomes of non-trypanosomes trypanosomatids. In *Leishmania major* and *Crithidia* spp*.*, which are phylogenetically related organisms [1,2], the position occupied by the PRAC gene was taken by a Tubby superfamily gene. In addition, these species also exhibited a putative enolase gene absent in this segment of trypanosome genomes (Figure 6). The insect trypanosomatids *Angomonas desouzai* and *Strigomonas culicis* lack both PRAC and Tubby family genes, while conserving the other flanking genes (data not shown). Therefore, corroborating the high plasticity of trypanosomatid genomes, the gain and loss of the PRAC gene in trypanosome genomes was not the only event that occurred in this genome segment during the evolution of the trypanosomatids.

### PRAC homologs from trypanosomes are largely under the influence of purifying selection

One common characteristic of HGT is that the horizontally acquired bacterial gene and host vertically inherited genes can differ in GC content and codon usage, depending on the relative timing of the HGT event [68-71]. Despite considerable GC content divergences among the genomes of different trypanosome species, the averages of the GC contents of whole genomes were comparable to those from *Try*PRAC and flanking genes, such as in the genomes of *T. cruzi* G (~49, 48 and 48%, respectively, for PRAC, PRAC loci and whole genome), *T. dionisii* (~52, 55, 47%), *T. vivax* (~49, 49, 46%) and *T. grayi* (~63, 55, 54%). Results indicating that the PRAC gene acquired by HGT has been strongly adapted to the codon usage of the host genes are consistent with ancient acquisition of the bacterial PRAC gene by one ancestor of *Trypanosoma*.

Protein sequences can continuously evolve under the effect of evolutionary pressure that arises as a consequence of the host-parasite interactions including host immune defences. To examine positive or negative selection pressures on the evolution of *Try*PRAC, we calculated the dN/dS ratio for the putative *Try*PRAC homologs. Finding dN/dS ratios below one, indicative of negative or purifying selection, suggested that positive selection is not the driving evolutionary force shaping the *Try*PRAC repertoire. In addition, codon selection was specifically investigated on *Try*PRAC motifs and residues essential for enzyme activity. The results indicated 40 negatively selected codons, 7 of which have known relevance to PRAC activity in *T. cruzi* and *T. vivax* (Figure 1), and no positively selected codon. PRAC homologs, despite evolving to be species and genotype specific, are largely under the influence of purifying selection. The fixation and evolution of PRAC genes under strong constraint in several trypanosomes suggests that PRAC activity should be advantageous to these parasites.

The genetic polymorphism analysis should be one of the first steps in the selection of promising vaccine and drug candidates [20]. A target sharing high conservation of all essential motifs in isolates representing the *T. cruzi* genetic repertoire, as evidenced previously for cruzipain [44] and herein demonstrated for *Tc*PRAC enzymes are good candidates for a multivalent drug against Chagas disease.

## Conclusions

*T. cruzi* and *T. vivax* PRAC enzymes are potent host B-cell mitogens that delay specific immune defences through the generation of non-specific B-cell proliferation, allowing parasite evasion and disease progression. These enzymes have also been linked to metabolism and parasite multiplication and differentiation. We identified *Try*PRAC homologs in the genomes of 12 trypanosome species, including newly sequenced genomes from trypanosomes of mammals, birds, snakes, lizards, crocodiles and toads. *Try*PRAC homologs were identified in most trypanosomes, including pathogenic and non-pathogenic species with different life cycles in vertebrates and vectors. *T. brucei* ssp., *T. evansi, T. congolense, T. simiae* and *T. godfreyi*, which are all pathogenic for mammals, were so far the only trypanosomes that lost PRAC genes.

The *Try*PRAC genealogy is congruent with the recognised relationships within *Trypanosoma*, with genes evolving to become species-specific and genotype-specific. A taxon-rich phylogenetic analysis strongly supports a bacterial origin for these genes. The presence of *Tc*PRAC homologs in trypanosomes of the Aquatic clade (basal in the phylogeny of *Trypanosoma*) and the absence of any PRAC-like gene in other trypanosomatid genera, bodonids and euglenids, together with the high synteny of PRAC gene neighbourhood allowed us to hypothesise that a common ancestor of *Trypanosoma* gained the bacterial gene through a single HGT. Our analysis supports a Firmicutes bacterium as the donor lineage, and suggested that the closest relatives of *Try*PRACs are not in the genus *Clostridium* of Clostridia class as previously hypothesized, but more likely in the genus *Gemella* of the Bacilli class. However, this conclusion can change as more and more genome sequences become available in this important bacterial group. The results revealed unique PRAC homologs for each species as well as for each *T. cruzi* DTU and *T. rangeli* lineages. According to *in silico* analysis, all newly identified putative *Try*PRAC genes likely express functional racemases except *T. rangeli*, which has only pseudogenes. Together, our results suggest evolutionarily driven rearrangements on *Try*PRAC loci resulting in the fixation of intact PRAC genes in most trypanosomes, complete loss by the subclade *T. brucei*-*T. congolense,* and PRAC genes, apparently, in process of being lost in *T. rangeli.*

An understanding of the repertoire and evolutionary history of genes encoding *Try*PRAC homologs in a range of trypanosome species and genotypes can help understand the potential role of PRAC enzymes in host-trypanosome interactions. Further analyses are required to evaluate the expression and any involvement of novel putative *Try*PRAC enzymes in the life cycles, infection strategies, pathogenicity, virulence, and host immune evasion of the various trypanosome species.

## Additional files

Additional file 1:
**Table containing the isolates of** ***T. cruzi*** **and** ***T. rangeli*** **and respective host species, geographic origin and lineages, and Genbank accession numbers of their respective PRAC gene sequences.**
Additional file 2:
**Genbank accession numbers of gGAPDH gene sequences included in the Figure** **2** **B.**
Additional file 3:
**(A)** **Network genealogy of entire PRAC** **amino-acid** **sequences from** ***T. cruzi*** **isolates of different DTUs.** Colors represent the DTUs, and the size of circles indicates the numbers of isolates. **(B)** Polymorphic amino acids detected on the alignment comprising 68 partial PRAC sequences from *T. cruzi* isolates of TcI-TcVI DTUs and Tcbat. Additional file 4:
**Alignment of predicted amino acid sequences of PRAC pseudogenes from** ***T. rangeli*** **isolates of all phylogenetic lineages (TrA-TrE). Essential motifs (MCGH and MIII) are in green, active site in red (SPCGT), R1, R2 and R3 in blue and Cys91/267 in yellow.** Red asterisks indicate stop codons. Additional file 5:
**Maximum likelihood phylogeny of 2,530** **PRAC-like** **protein sequences (Figure** **5** **A) displayed as rectangular phylogram.**
Additional file 6:
**Genbank acession numbers of 2,530** **PRAC-like** **family genes from prokaryotes and eukaryotes retrieved from full NCBI NR database and included in the Figure** **5** **A.**
Additional file 7:
**Genbank accession numbers of 303 prokaryotic** **PRAC-like** **genes closest related to** ***Try*** **PRAC genes retrieved from full NCBI NR database and included in the Figure** 5 **B.**
