# Supplementary material for: Phylogenetic and syntenic data support a single horizontal transference to a Trypanosoma ancestor of a prokaryotic proline racemase implicated in parasite evasion from host defences
Source: Parasit Vectors. 2015 Apr 12;8:222. doi: 10.1186/s13071-015-0829-y (PMC4417235; doi:10.1186/s13071-015-0829-y)
Supplement: Additional file 1: — Table containing the isolates of T. cruzi and T. rangeli and respective host species, geographic origin and lineages, and Genbank accession numbers of their respective PRAC gene sequences. [file 13071_2015_829_MOESM1_ESM.pdf]

Additional File 1

Trypanosoma cruzi and *T. rangeli* isolates characterized in this study and respective PRAC homologous genes

TCC code/SAG	species/isolate	DTU/ lineage	Host species	Geographic origin	GenBank Acession number#
Trypanosoma cruzi					
1321	Dm28	TcI	*Didelphismarsupialis*	Colombia	KP001300
1324-1325	MM1, FC1	TcI	*Homo sapiens*	Panama	KP001276
1832		TcI	*Lutralongicaudis*	Panama	
1834		TcI	*Saguinusgeoffroyi*	Panama	KP001282
1837		TcI	*Triatoma dimidiata*	Panama	KP001283
1840/1841		TcI	*Rhodniuspallescens*	Panama	
	TcMex1	TcI	*Homo sapiens*	Mexico	
	TcMex8, TcMex11	TcI	*Triatoma* sp.	Mexico	KP001306
	TcDm1	TcI	*Didelphismarsupialis*	Colombia	
588		TcI	*Homo sapiens*	Guatemala	
1474/1478	Tc297, Tc293	TcI	*Cavia*sp.	Peru	KP001279, KP001280
	Tc3,11, 23	TcI	*Homo sapiens*	Venezuela	KP001311, KP001310
536		TcI	*Rattusrattus*	Venezuela	KP001286
1401	TcRP1	TcI	*Rhodniusprolixus*	Venezuela	KP001277, KP001278
1338		TcI	*Carolliaperspicillata*	Venezuela	
	TcDm36	TcI	*Didelphismarsupialis*	Venezuela	
183		TcI	*Triatoma infestans*	Bolivia	
1990	ATCC30823	TcI	*Canisfamiliaris*	USA	KP001284
1989	ATCC30160	TcI	*Homo sapiens*	USA	
593		TcI	*Triatoma dimidiata*	Guatemala	KP001288
34E	Y	TcII	*Homo sapiens*	Brazil	
137,139	TcEP23X,TcIB42X	TcII	*Didelphismarsupialis*	Brazil	KP001303
1508	TcJac	TcII	*Homo sapiens*	Brazil	KP001281
873	Tc573	TcII	*Homo sapiens*	Brazil	KP001287
845	MT3663	TcIII	*Panstrongylusgeniculatus*	Brazil	
844	MT3869	TcIII	*Homo sapiens*	Brazil	
863,864		TcIII	*Euphractussexcinctus*	Brazil	KP001295, KP001292
132	TcIB74P	TcIII	*Philanderfrenatus*	Brazil	
1323,1386		TcIII	*Canisfamiliaris*	Brazil	
1437	TcB6338	TcIII	*Proechimyslongicaudatus*	Brazil	
1356	TcB6056	TcIII	*Oxymycterus*sp.	Brazil	
135	TcIB14X	TcIII	*Proechimysiheringi*	Brazil	KP001297
1078-80		TcIII	*Triatoma rubrovaria*	Brazil	
	Arma13cl1	TcIII	*Dasypusnovemcinctus*	Paraguay	KP001296
85	JJ	TcIV	*Homo sapiens*	Brazil	

82,778	RbX, Rb778	TcIV	*Rhodniusbrethesi*	Brazil	KP001291, KP001290
	STC, 92122	TcIV	*Procyonlotor*	USA	KP001309, KP001293
967	NRCL3	TcV	*Homo sapiens*	Chile	KP001307, KP001308
185	185	TcV	*Triatoma infestans*	Bolivia	
656	656	TcV	*Homo sapiens*	Bolivia	KP001289
33E	CL	TcVI	*Triatoma infestans*	Brazil	KP001299
294		Tcbat	*Myotislevis*	Brazil	KP001285
793		Tcbat	*Myotislevis*	Brazil	
947		Tcbat	*Myotisnigricans*	Brazil	KP001294
203		Tcbat	*Myotisruber*	Brazil	
1122		Tcbat	*Myotisalbescens*	Brazil	KP001275
Trypanosoma cruzi marinkellei					
1705			*Artibeusplanirostris*	Brazil	
Trypanosoma rangeli					
031	San Agustin	A	*Homo sapiens*	Colombia	
594	SMH-79	A	*Homo sapiens*	Guatemala	
1257	IM5051	A	*Saguinus bicolor*	Brazil	
701	ROR-62	A	*Rhodniusrobustus*	Brazil	KP001265
238	5-31	B	*Saguinuslabiatus*	Brazil	KP001266
010	Legeri	B	*Tamanduatetradactyla*	Brazil	KP001267
014	PG	C	*Homo sapiens*	Panama	KP001268
1260	Pa479GS	C	*Rhodniuspallescens*	Panama	KP001269
1952		C	*Saguinusgeoffroyi*	Panama	KP001270
023	SC58	D	*Echimysdasythrix*	Brazil	KP001271
643	Tra643	E	*Platyrrhinuslineatus*	Brazil	KP001272
1224	IM5040	E	*Rhodniuspictipes*	Brazil	KP001273
1225	IM5136	E	*Rhodniuspictipes*	Brazil	KP001274
1228	IM5120	E	*Rhodniuspictipes*	Brazil	

#, Sequences representing the whole diversity of PRAC gene sequences from isolates representing all *T. cruzi* DTUs and all lineages of *T. rangeli* deposited in GenBank
