# Supplementary material for: Phylogenetic and syntenic data support a single horizontal transference to a Trypanosoma ancestor of a prokaryotic proline racemase implicated in parasite evasion from host defences
Source: Parasit Vectors. 2015 Apr 12;8:222. doi: 10.1186/s13071-015-0829-y (PMC4417235; doi:10.1186/s13071-015-0829-y)
Supplement: Additional file 2: — Genbank accession numbers of gGAPDH gene sequences included in the Figure 2 B. [file 13071_2015_829_MOESM2_ESM.pdf]

Additional File 2

GenBank accession numbers of gGAPDH gene sequences included in the Figure 2B

***Trypanosoma* species**

Trypanosoma sp. from toad TCC339 (KP686217), *Trypanosoma* sp. from lizard TCC878 (KP686218), *Trypanosoma* sp. from bird TCC1825 (KP686219) *Trypanosoma terena* (EU596256), *T. ralphi* (EU596257), *T. grayi* (AJ620258), *T. microti* (AJ620273), *T. lewisi* (AJ620272), *T. rangeli* (AF053742), *T. vespertilionis* (AJ620283), *T. conorhini* (AJ620267), *T. dionisii* (FJ649494), *T. cruzi* (X52898), *T. cruzi marinkellei* (AJ620270), *T.* sp. AAT (AJ620264), *T. congolense* (AJ620291), *T. brucei rhodesiense* (AJ620284), *T. cascavelli* (FJ236511), *T. serpentis* (FJ236512), *T.* sp. Gecko (AJ620259), *T. varani* (AJ620261), *T. cyclops* (AJ620265), *T.* sp. wallaby ABF (AJ620278), *T. theileri* (AJ620282), *T.* sp. D30 (AJ620279), *T. rotatorium* (AJ620256), *T. mega* (AJ620253), *T. vivax* (AF053744), *T. erneyi* (JN040964), *T. corvi* OA6 (HQ906660), *T. avium* chaffinch (AJ620262), *T. avium* rook (AJ620263)

Non-trypanosome trypanosomatids

Angomonas deanei (HM593022), *Angomonas desouzai* (HM593020), *Leptomonas seymouri* (AF047495), *Herpetomonas megaseliae* (DQ092547), *Herpetomonas muscarum* (DQ092548), *Herpetomonas samuelpessoai* (AF047494), *Phytomonas* sp. (AF047496), *Crithidia fasciculata* (AF053739), *Wallaceina brevicula* (AF316620), *Leishmania tarentolae* (DQ092549), *Leishmania major* (AF047497).

Bodonids and Euglenids

Euglena gracilis (L21903), *Eutreptiella* sp. (AB106705), *Parabodo caudatus* (DQ915848), *Bodo saltans* (DQ915847).
