# Supplementary figures and images for: Phylogenetic and syntenic data support a single horizontal transference to a Trypanosoma ancestor of a prokaryotic proline racemase implicated in parasite evasion from host defences
Source: Parasit Vectors. 2015 Apr 12;8:222. doi: 10.1186/s13071-015-0829-y (PMC4417235; doi:10.1186/s13071-015-0829-y)

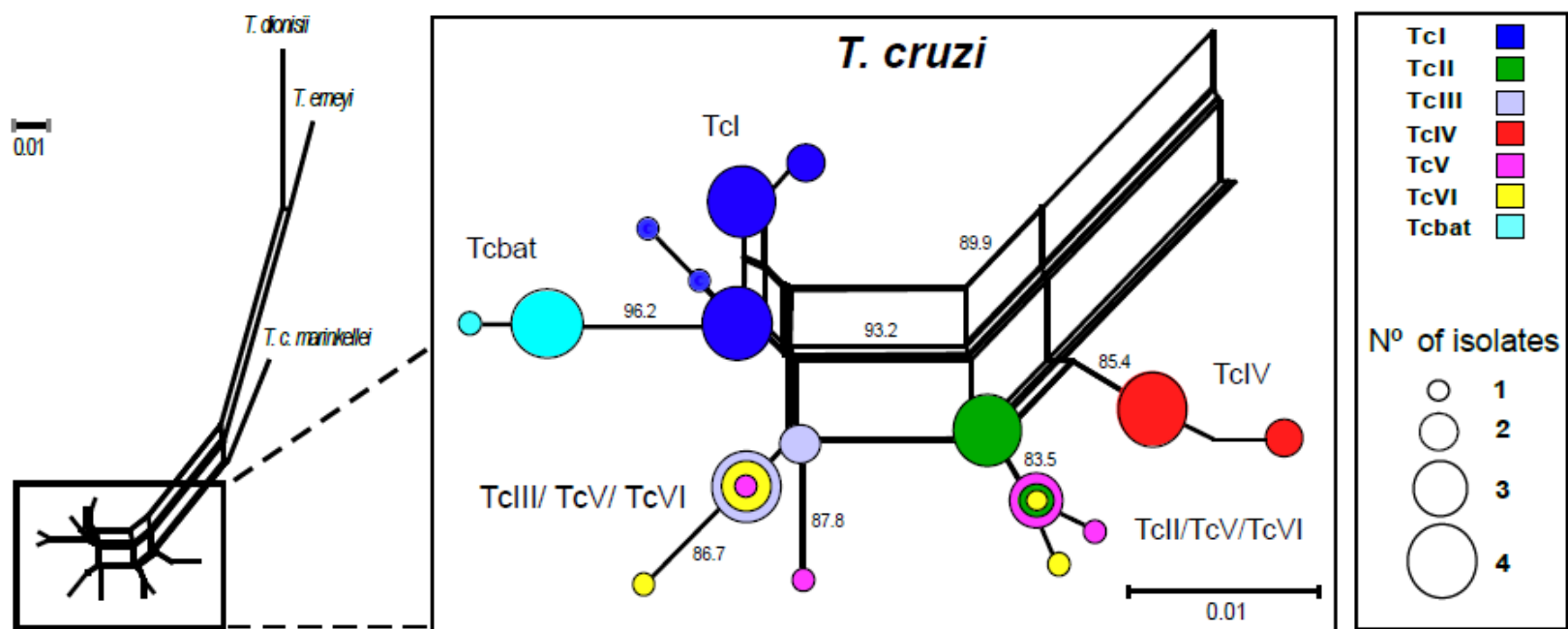

Supplement: Additional file 3: — (A) Network genealogy of entire PRAC amino-acid sequences from T. cruzi isolates of different DTUs. Colors represent the DTUs, and the size of circles indicates the numbers of isolates. (B) Polymorphic amino acids detected on the alignment comprising 68 partial PRAC sequences from T. cruzi isolates of TcI-TcVI DTUs and Tcbat. [file 13071_2015_829_MOESM3_ESM.pdf]

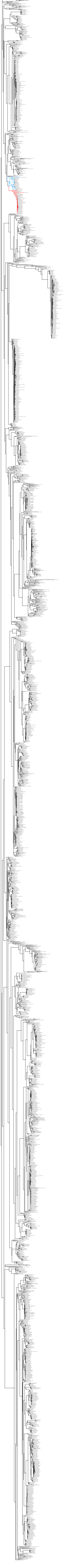

Supplement: Additional file 5: — Maximum likelihood phylogeny of 2,530 PRAC-like protein sequences (Figure 5 A) displayed as rectangular phylogram. [file 13071_2015_829_MOESM5_ESM.pdf]
