# Supplementary material for: Phylogenetic and syntenic data support a single horizontal transference to a Trypanosoma ancestor of a prokaryotic proline racemase implicated in parasite evasion from host defences
Source: Parasit Vectors. 2015 Apr 12;8:222. doi: 10.1186/s13071-015-0829-y (PMC4417235; doi:10.1186/s13071-015-0829-y)
Supplement: Additional file 6: — Genbank acession numbers of 2,530 PRAC-like family genes from prokaryotes and eukaryotes retrieved from full NCBI NR database and included in the Figure 5 A. [file 13071_2015_829_MOESM6_ESM.pdf]

Additional File 6

Access numbers of PRAC-like sequences retrieved from NR NCBI databank included in the Figure 5B

GI	Access number	Organism
6900003	CAB71312.1	Clostridium sticklandii
30020954	NP_832585.1	Bacillus cereus ATCC 14579
30262803	NP_845180.1	Bacillus anthracis str. Ames
42781920	NP_979167.1	Bacillus cereus ATCC 10987
49476772	YP_036919.1	Bacillus thuringiensis serovar konkukian str. 97-27
52142682	YP_084148.1	Bacillus cereus E33L
55379132	YP_136982.1	Haloarcula marismortui ATCC 43049
71400794	XP_803162.1	Trypanosoma cruzi strain CL Brener
71419827	XP_811287.1	Trypanosoma cruzi strain CL Brener
74812220	Q868H8.1	Trypanosoma cruzi strain CL Brener
77465520	YP_355023.1	Rhodobacter sphaeroides 2.4.1
90108457	1W61	Trypanosoma cruzi
118478186	YP_895337.1	Bacillus thuringiensis str. Al Hakam
126463923	YP_001045036.1	Rhodobacter sphaeroides ATCC 17029
126700857	YP_001089754.1	Peptoclostridium difficile 630
142933105	ABO92935.1	Trypanosoma vivax
148380430	YP_001254971.1	Clostridium botulinum A str. ATCC 3502
150388506	YP_001318555.1	Alkaliphilus metalliredigens QYMF
152113098	Q4DA80.2	Trypanosoma cruzi strain CL Brener
158319092	YP_001511599.1	Alkaliphilus oremlandii OhILAs
163940583	YP_001645467.1	Bacillus weihenstephanensis KBAB4
169825719	YP_001695877.1	Lysinibacillus sphaericus C3-41
217960275	YP_002338835.1	Bacillus cereus AH187
218232893	YP_002367554.1	Bacillus cereus B4264
218897859	YP_002446270.1	Bacillus cereus G9842
218903956	YP_002451790.1	Bacillus cereus AH820
220904954	YP_002480266.1	Desulfovibrio desulfuricans subsp. desulfuricans str. ATCC 27774
222096333	YP_002530390.1	Bacillus cereus Q1
225864808	YP_002750186.1	Bacillus cereus 03BB102
226314148	YP_002774044.1	Brevibacillus brevis NBRC 100599
237795912	YP_002863464.1	Clostridium botulinum Ba4 str. 657
242398623	YP_002994047.1	Thermococcus sibiricus MM 739
292653723	YP_003533621.1	Haloferax volcanii DS2
296503369	YP_003665069.1	Bacillus thuringiensis BMB171
298293153	YP_003695092.1	Starkeya novella DSM 506
301054356	YP_003792567.1	Bacillus cereus biovar anthracis str. CI
302390215	YP_003826036.1	Thermosediminibacter oceani DSM 16646
310659532	YP_003937253.1	Clostridium sticklandii
311108024	YP_003980877.1	Achromobacter xylosoxidans A8
332685776	YP_004455550.1	Melissococcus plutonius ATCC 35311
336118063	YP_004572831.1	Microlunatus phosphovorus NM-1
344171589	CCA84206.1	Ralstonia syzygii R24
344213146	YP_004797466.1	Haloarcula hispanica ATCC 33960
348025670	YP_004765474.1	Megasphaera elsdenii DSM 20460
374322435	YP_005075564.1	Paenibacillus terrae HPL-003
376266697	YP_005119409.1	Bacillus cereus F837/76
379726838	YP_005319023.1	Melissococcus plutonius DAT561
384180721	YP_005566483.1	Bacillus thuringiensis serovar finitimus YBT-020
384186897	YP_005572793.1	Bacillus thuringiensis serovar chinensis CT-43
392379308	YP_004986467.1	Azospirillum brasilense Sp245
392408252	YP_006444860.1	Anaerobaculum mobile DSM 13181
392989503	YP_006488096.1	Enterococcus hirae ATCC 9790

402556956	YP_006598227.1	Bacillus cereus FRI-35
402559839	YP_006602563.1	Bacillus thuringiensis HD-771
407396786	EKF27546.1	Trypanosoma cruzi marinkellei
407705259	YP_006828844.1	Bacillus thuringiensis MC28
407832562	EKF98492.1	Trypanosoma cruzi
410675203	YP_006927574.1	Bacillus thuringiensis Bt407
410866962	YP_006981573.1	Propionibacterium acidipropionici ATCC 4875
434375812	YP_006610456.1	Bacillus thuringiensis HD-789
446764790	WP_000842046.1	Bacillus cereus
446764792	WP_000842048.1	Bacillus cereus
446764794	WP_000842050.1	Bacillus cereus group
446764795	WP_000842051.1	Bacillus cereus
447180261	WP_001257517.1	Bacillus cereus
447180263	WP_001257519.1	Bacillus cereus
447180264	WP_001257520.1	Bacillus cereus
447180265	WP_001257521.1	Bacillus cereus
447180266	WP_001257522.1	Bacillus cereus
447180267	WP_001257523.1	Bacillus cereus
447180273	WP_001257529.1	Bacillus cereus
447180274	WP_001257530.1	Bacillus cereus
447180276	WP_001257532.1	Bacillus cereus
447180278	WP_001257534.1	Bacillus cereus
447180279	WP_001257535.1	Bacillus cereus
447180287	WP_001257543.1	Bacillus cereus
447180290	WP_001257546.1	Bacillus cereus
447180291	WP_001257547.1	Bacillus cereus
447180292	WP_001257548.1	Bacillus cereus
447180295	WP_001257551.1	Bacillus cereus
447180300	WP_001257556.1	Bacillus cereus
447183018	WP_001260274.1	Bacillus cereus
447183024	WP_001260280.1	Bacillus anthracis
470203393	YP_007594105.1	Bifidobacterium thermophilum RBL67
479173712	YP_007801695.1	Gordonibacter pamelaeae 7-10-1-b
487902233	WP_001975699.1	Bacillus cereus
487924190	WP_001997656.1	Bacillus cereus
487926495	WP_001999961.1	Bacillus cereus
487929472	WP_002002938.1	Bacillus cereus group
487931755	WP_002005221.1	Bacillus cereus
487934372	WP_002007838.1	Bacillus cereus
487939511	WP_002012977.1	Bacillus cereus
487945007	WP_002018473.1	Bacillus cereus
487948170	WP_002021636.1	Bacillus cereus
487950825	WP_002024291.1	Bacillus cereus
487952463	WP_002025929.1	Bacillus cereus
487954379	WP_002027844.1	Bacillus cereus
487959250	WP_002032610.1	Bacillus cereus
487963172	WP_002036436.1	Bacillus cereus group
487965708	WP_002038840.1	Bacillus cereus
487968250	WP_002041290.1	Bacillus cereus group
487970888	WP_002043811.1	Bacillus cereus
487980661	WP_002053390.1	Bacillus cereus
487983235	WP_002055853.1	Bacillus cereus
487989268	WP_002061584.1	Bacillus cereus
487993634	WP_002065759.1	Bacillus cereus
488000158	WP_002072035.1	Bacillus cereus
488004556	WP_002076249.1	Bacillus cereus group
488038931	WP_002110328.1	Bacillus cereus
488048603	WP_002120000.1	Bacillus cereus

488065364	WP_002136761.1	Bacillus cereus
488070697	WP_002142094.1	Bacillus cereus
488075934	WP_002147331.1	Bacillus cereus
488090091	WP_002161488.1	Bacillus cereus
488094225	WP_002165622.1	Bacillus cereus
488100913	WP_002172310.1	Bacillus cereus
488114254	WP_002185651.1	Bacillus cereus
488124427	WP_002195635.1	Bacillus cereus
488126659	WP_002197867.1	Bacillus cereus
488129018	WP_002200226.1	Bacillus cereus
488681165	WP_002608780.1	Erysipelotrichaceae
488811975	WP_002724381.1	Rhodobacter
488932400	WP_002843475.1	Peptostreptococcus
489239550	WP_003147788.1	Gemella haemolysans
489290531	WP_003198076.1	Bacillus mycoides
489299872	WP_003207346.1	Bacillus mycoides
489333269	WP_003240511.1	Bacillus subtilis
489360017	WP_003266965.1	Bacillus thuringiensis
489367635	WP_003274464.1	Bacillus thuringiensis
489371853	WP_003278635.1	Bacillus thuringiensis
489395032	WP_003301541.1	Bacillus thuringiensis
489400802	WP_003307202.1	Bacillus thuringiensis
489798985	WP_003702876.1	Lactobacillus salivarius
489810161	WP_003714017.1	Lactobacillus oris
489811188	WP_003715042.1	Lactobacillus oris
490197903	WP_004096428.1	Acetonema longum
490337899	WP_004224302.1	Lysinibacillus fusiformis
490540505	WP_004405641.1	Bacillus thuringiensis
490576384	WP_004441404.1	Clostridium botulinum
490651098	WP_004516092.1	Haloarcula vallismortis
490728202	WP_004590607.1	Haloarcula japonica
490745003	WP_004607311.1	Clostridium scindens
490751284	WP_004613592.1	Clostridiales
490771798	WP_004634022.1	Gemella morbillorum
491097603	WP_004959204.1	Haloarcula sinaiiensis
491119548	WP_004977990.1	Haloferax gibbonsii
491165907	WP_005024278.1	Bilophila wadsworthia
491680942	WP_005537083.1	Haloarcula argentinensis
491684031	WP_005540169.1	Johnsonella ignava
492406589	WP_005834650.1	Bacillales
492750836	WP_005948987.1	Blautia hydrogenotrophica
493019249	WP_006095057.1	Bacillus pseudomycoides
493042320	WP_006106937.1	Natrialba asiatica
493146288	WP_006159973.1	Cupriavidus basilensis
493253520	WP_006220994.1	Achromobacter piechaudii
493269141	WP_006227860.1	Achromobacter piechaudii
493407964	WP_006363981.1	Gemella sanguinis
493435119	WP_006390651.1	Achromobacter xylosoxidans
493472321	WP_006427363.1	Dorea longicatena
493478044	WP_006432982.1	Natrinema versiforme
493484901	WP_006439715.1	Clostridium hiranonis
493490252	WP_006444975.1	Clostridium hylemonae
493717294	WP_006666828.1	Natrialba aegyptia
493725064	WP_006674436.1	Halobiforma nitratireducens
493842604	WP_006789705.1	Anaeroglobus geminatus
493877956	WP_006824303.1	Natrialba taiwanensis
493977600	WP_006920611.1	Bacillus sp. GeD10
494000017	WP_006942606.1	Megasphaera micronuciformis

494122896	WP_007062673.1	Clostridium carboxidivorans
494122905	WP_007062682.1	Clostridium carboxidivorans
494200202	WP_007124906.1	Lactobacillus antri
494232992	WP_007140037.1	Halobiforma lacisalsi
494273400	WP_007158106.1	Oribacterium sinus
494347286	WP_007189434.1	Haloarcula californiae
494486947	WP_007276420.1	Haloferax sulfurifontis
494673993	WP_007431933.1	Paenibacillus sp. Aloe-11
494989837	WP_007715857.1	Brevibacillus sp. BC25
495062684	WP_007787514.1	Brevibacillus sp. CF112
495369461	WP_008094174.1	Haloferax prahovense
495452331	WP_008177025.1	Bacillus sp. B14905
495573414	WP_008297993.1	Bhargavaea cecembensis
495582862	WP_008307441.1	Haloarcula amylolytica
495600043	WP_008324622.1	Haloferax elongans
495647913	WP_008372492.1	Coprococcus comes
495664170	WP_008388749.1	Halosarcina pallida
495847922	WP_008572501.1	Haloferax
495870406	WP_008594985.1	Nitratireductor pacificus
495882984	WP_008607563.1	Haloferax sp. BAB2207
496092930	WP_008817437.1	Firmicutes
496171357	WP_008895864.1	Haloterrigena salina
496244391	WP_008957776.1	Halomonas sp. HAL1
496263625	WP_008977010.1	Lachnospiraceae
496289811	WP_009001849.1	Clostridium sp. D5
496460564	WP_009169409.1	Clostridium sp. DL-VIII
496505691	WP_009213974.1	Oribacterium sp. oral taxon 078
496542038	WP_009248186.1	Lachnospiraceae bacterium 5_1_57FAA
496557251	WP_009263326.1	Lachnospiraceae
496588255	WP_009286883.1	Halomonas titanicae
496690290	WP_009331833.1	Bacillus sp. 2_A_57_CT2
496774940	WP_009368752.1	Bilophila sp. 4_1_30
496829151	WP_009376761.1	Halogranum salarium
496992334	WP_009429838.1	Oribacterium sp. oral taxon 108
497077691	WP_009461342.1	Lachnospiraceae bacterium 2_1_46FAA
497220727	WP_009534989.1	Oribacterium parvum
497223232	WP_009537494.1	Oribacterium asaccharolyticum
497329448	WP_009643661.1	Mogibacterium sp. CM50
497329749	WP_009643962.1	Mogibacterium sp. CM50
497711481	WP_010025665.1	Sporolactobacillus inulinus
497980698	WP_010294854.1	Clostridium senegalense
498033334	WP_010347490.1	Paenibacillus peoriae
498310803	WP_010624959.1	Lactobacillus versmoldensis
498315795	WP_010629951.1	Halomonas sp. KM-1
498434013	WP_010739705.1	Enterococcus malodoratus
498435912	WP_010741592.1	Enterococcus malodoratus
498438345	WP_010744008.1	Enterococcus raffinosus
498452247	WP_010757752.1	Enterococcus pallens
498474540	WP_010778865.1	Enterococcus gilvus
498476948	WP_010781267.1	Enterococcus gilvus
506990671	WP_016081265.1	Bacillus cereus
507012007	WP_016087521.1	Bacillus cereus
507019432	WP_016091545.1	Bacillus cereus
507029534	WP_016101254.1	Bacillus cereus
507033398	WP_016105097.1	Bacillus cereus
507036894	WP_016108577.1	Bacillus cereus
507044065	WP_016115119.1	Bacillus cereus
507051687	WP_016122676.1	Bacillus

507062085	WP_016132924.1	Bacillus cereus
510804130	WP_016178899.1	Enterococcus avium
510885559	WP_016220098.1	Dorea sp. 5-2
510889445	WP_016222783.1	Lachnospiraceae bacterium 3-2
510890479	WP_016223794.1	Lachnospiraceae bacterium 3-2
512440296	WP_016416105.1	Halomonas anticariensis
513823126	WP_016512683.1	Bacillus cereus
515112335	WP_016741395.1	Brevibacillus brevis
515239020	WP_016821425.1	Paenibacillus polymyxa
515567843	WP_017000674.1	Staphylococcus lentus
515821443	WP_017252196.1	Brevibacillus brevis
515995749	WP_017426332.1	Paenibacillus sp. ICGEB2008
516119230	WP_017549810.1	Salinicoccus carnicancri
516129580	WP_017560160.1	Bacillus sp. WBUNB001
516269298	WP_017673261.1	Bacillus sp. WBUNB004
516338807	WP_017728840.1	Bacillus sp. L1(2012)
516361638	WP_017751671.1	Clostridium tyrobutyricum
517200077	WP_018388895.1	Xanthobacteraceae
517421282	WP_018592765.1	Terrisporobacter glycolicus
517493005	WP_018663582.1	Thermobrachium celere
517536735	WP_018706943.1	Bacillus fordii
517582363	WP_018752571.1	Paenibacillus sanguinis
517595458	WP_018765666.1	Bacillus sp. 105MF
517610430	WP_018780638.1	Bacillus sp. 95MFCvi2.1
517752843	WP_018923051.1	Salsuginibacillus kocurii
517999945	WP_019170153.1	Pseudaminobacter salicylatoxidans
518071528	WP_019241736.1	Bacillus massilioanorexius
518207255	WP_019377463.1	Virgibacillus halodenitrificans
518213100	WP_019383308.1	Bacillus oceanisediminis
518223348	WP_019393556.1	Bacillus endophyticus
518373066	WP_019543273.1	Selenomonas bovis
518652147	YP_008141667.1	Ferroplasma acidarmanus fer1
518850425	WP_020006315.1	Salinicoccus albus
518904221	WP_020060096.1	Bacillus sp. 123MFChir2
520879191	WP_020310842.1	Megasphaera
528985416	YP_008030733.1	Achromobacter xylosoxidans NH44784-1996
530547727	YP_008428259.1	Thermococcus litoralis DSM 5473
544695260	WP_021126550.1	Clostridium sordellii
544699021	WP_021130144.1	Clostridium sordellii
544705636	WP_021136255.1	Clostridium botulinum
544970884	WP_021373750.1	Peptoclostridium difficile
544988145	WP_021386243.1	Peptoclostridium difficile
545031873	WP_021412177.1	Peptoclostridium difficile
545034084	WP_021413071.1	Peptoclostridium difficile
545057010	WP_021430157.1	Clostridium bifermentans
545061010	WP_021434104.1	Clostridium bifermentans
545089756	WP_021459396.1	Staphylococcus sp. EGD-HP3
545398543	WP_021638234.1	Clostridium sp. KLE 1755
545645245	WP_021752997.1	Gemella bergeri
545668688	WP_021775011.1	Oribacterium sp. oral taxon 078
546145506	WP_021788041.1	Ferroplasma sp. Type II
546357443	WP_021844514.1	Blautia hydrogenotrophica CAG:147
546420484	WP_021858850.1	Firmicutes bacterium CAG:83
546428562	WP_021859784.1	Dorea sp. CAG:105
547779839	WP_022190796.1	Firmicutes bacterium CAG:240
547811030	WP_022220198.1	Coprococcus comes CAG:19
547916271	WP_022319005.1	Dorea sp. CAG:317
547958439	WP_022358812.1	Clostridium sp. CAG:43

548195963	WP_022416476.1	Dorea longicatena CAG:42
548433789	WP_022523626.1	Halomonas
557619685	YP_008780816.1	Bacillus toyonensis BCT-7112
557861670	ESS65020.1	Trypanosoma cruzi Dm28c
558681348	YP_008819477.1	Bacillus thuringiensis YBT-1518
559781388	GAE02922.1	Clostridium botulinum B str. Osaka05
563693667	YP_008868650.1	Spiroplasma apis B31
565990887	YP_008910479.1	Paenibacillus polymyxa CR1
568129811	YP_008926458.1	Achromobacter xylosoxidans NBRC 15126 = ATCC 27061
571138016	ETP67359.1	Planomicrobium glaciei CHR43
573024906	AHF80440.1	Thermococcus sp. ES1
573481958	AHG00369.1	Halostagnicola larsenii XH-48
573589845	ETT88049.1	Viridibacillus arenosi FSL R5-213
574961650	CDK35962.1	Lactobacillus salivarius cp400
576980661	EUC53405.1	Mogibacterium timidum ATCC 33093
584381297	EWG98416.1	Halomonas sp. BC04
589236709	CDN36294.1	Bacillus thuringiensis DB27
595632423	AHM64681.1	Paenibacillus polymyxa SQR-21
612166716	EZQ03357.1	Azospirillum brasilense
