# Supplementary material for: Phylogenetic and syntenic data support a single horizontal transference to a Trypanosoma ancestor of a prokaryotic proline racemase implicated in parasite evasion from host defences
Source: Parasit Vectors. 2015 Apr 12;8:222. doi: 10.1186/s13071-015-0829-y (PMC4417235; doi:10.1186/s13071-015-0829-y)
Supplement: Additional file 7: — Genbank accession numbers of 303 prokaryotic PRAC-like genes closest related to Try PRAC genes retrieved from full NCBI NR database and included in the Figure 5 B. [file 13071_2015_829_MOESM7_ESM.pdf]

Additional File 7

PRAC-like sequences (2,530) retrieved from NR NCBI databank included in Figure 5A

GI	Access number	Organism
152113098	Q4DA80.2	Trypanosoma cruzi strain CL Brener
74812220	Q868H8.1	Trypanosoma cruzi strain CL Brener
50513779	1TM0	Brucella Melitensis
90108457	1W61	Trypanosoma cruzi
459359065	4JD7	Pseudomonas putida F1
460417677	4J9W	Pseudomonas Fluorescens Pf-5
460417683	4JCI	Chromohalobacter salexigens DSM 3043
480312348	4JUU	Xanthomonas campestris ATCC 33913
485602245	4K7G	Agrobacterium vitis S4
524935068	4LB0	Agrobacterium vitis S4
470395539	XP_004335083.1	Acanthamoeba castellanii str. Neff
493607961	WP_006560430.1	Acetobacter tropicalis
490197903	WP_004096428.1	Acetonema longum
490194189	WP_004092757.1	Acetonema longum
493269141	WP_006227860.1	Achromobacter piechaudii
493253520	WP_006220994.1	Achromobacter piechaudii
493250286	WP_006218157.1	Achromobacter piechaudii
493251632	WP_006219497.1	Achromobacter piechaudii
493435119	WP_006390651.1	Achromobacter xylosoxidans
311108024	YP_003980877.1	Achromobacter xylosoxidans A8
568129811	YP_008926458.1	Achromobacter xylosoxidans NBRC 15126 = ATCC 27061
528985416	YP_008030733.1	Achromobacter xylosoxidans NH44784-1996
522203526	WP_020710993.1	Acidobacteriaceae bacterium KBS 83
522208073	WP_020715540.1	Acidobacteriaceae bacterium KBS 89
522213307	WP_020720419.1	Acidobacteriaceae bacterium KBS 96
225874816	YP_002756275.1	Acidobacterium capsulatum ATCC 51196
498144400	WP_010458556.1	Acidovorax radicis
495136647	WP_007861455.1	Acidovorax sp. CF316
407938907	YP_006854548.1	Acidovorax sp. KKS102
496181775	WP_008906282.1	Acidovorax sp. NO-1
446355158	WP_000433013.1	Acinetobacter baumannii
491252537	WP_005110721.1	Acinetobacter baumannii
447189907	WP_001267163.1	Acinetobacter baumannii
446782327	WP_000859583.1	Acinetobacter baumannii
446782335	WP_000859591.1	Acinetobacter baumannii
446782326	WP_000859582.1	Acinetobacter baumannii
446782336	WP_000859592.1	Acinetobacter baumannii
490885674	WP_004747643.1	Acinetobacter baumannii
446782331	WP_000859587.1	Acinetobacter baumannii
446782330	WP_000859586.1	Acinetobacter baumannii
446782333	WP_000859589.1	Acinetobacter baumannii
446782329	WP_000859585.1	Acinetobacter baumannii
490979914	WP_004841677.1	Acinetobacter baumannii
446782323	WP_000859579.1	Acinetobacter baumannii
496867101	WP_009391071.1	Acinetobacter baumannii
545154991	WP_021510279.1	Acinetobacter baumannii
488061761	WP_002133158.1	Acinetobacter baumannii
497201168	WP_009515430.1	Acinetobacter baumannii
489248699	WP_003156842.1	Acinetobacter baumannii
589430595	EXH14489.1	Acinetobacter baumannii 1245593
589465914	EXH47895.1	Acinetobacter baumannii 1412924
588244136	EXF15912.1	Acinetobacter baumannii 1417041
588189366	EXE63037.1	Acinetobacter baumannii 1542444
593952228	EXU05546.1	Acinetobacter baumannii 25253_5
610950633	EZI57820.1	Acinetobacter baumannii 25442_7
595126418	EXV52128.1	Acinetobacter baumannii 25935_8
595120483	EXV46359.1	Acinetobacter baumannii 25935_9
587910944	EXB98807.1	Acinetobacter baumannii 342950
610979460	EZI85605.1	Acinetobacter baumannii 44298_4
598506093	EYC59740.1	Acinetobacter baumannii 44437_9
587903092	EXB91321.1	Acinetobacter baumannii 466760
593644695	EXS24779.1	Acinetobacter baumannii 573719
588085619	EXD65791.1	Acinetobacter baumannii 58452
587920665	EXC08096.1	Acinetobacter baumannii 625974
587953068	EXC38809.1	Acinetobacter baumannii 951631
184157646	YP_001845985.1	Acinetobacter baumannii ACICU
126641371	YP_001084355.1	Acinetobacter baumannii ATCC 17978
169796424	YP_001714217.1	Acinetobacter baumannii AYE
523530630	YP_008216657.1	Acinetobacter baumannii BJAB0715
469821514	YP_007595884.1	Acinetobacter baumannii D1279779
571614495	ETQ06698.1	Acinetobacter baumannii UH12208
571667851	ETQ58310.1	Acinetobacter baumannii UH22908
491173225	WP_005031591.1	Acinetobacter bereziniae
375134290	YP_004994940.1	Acinetobacter calcoaceticus PHEA-2
446782324	WP_000859580.1	Acinetobacter calcoaceticus/baumannii complex
490847449	WP_004709512.1	Acinetobacter nosocomialis
549993313	WP_022574849.1	Acinetobacter nosocomialis

299770655	YP_003732681.1	Acinetobacter oleivorans DR1
507070111	WP_016140889.1	Acinetobacter pittii
507070094	WP_016140872.1	Acinetobacter pittii
446782334	WP_000859590.1	Acinetobacter sp. NCTC 10304
491399781	WP_005257627.1	Acinetobacter sp. NIPH 2168
491427671	WP_005285466.1	Acinetobacter sp. NIPH 3623
491446300	WP_005304087.1	Acinetobacter sp. NIPH 542
490931294	WP_004793147.1	Acinetobacter sp. NIPH 817
490918705	WP_004780582.1	Acinetobacter sp. NIPH 899
497272648	WP_009586865.1	Acinetobacter sp. WC-743
518690062	WP_019851755.1	actinobacterium SCGC AAA027-J17
497297737	WP_009611954.1	actinobacterium SCGC AAA027-L06
517037801	WP_018226619.1	actinobacterium SCGC AAA028-A23
517034897	WP_018224252.1	actinobacterium SCGC AAA278-I18
516580466	WP_017955505.1	actinobacterium SCGC AAA278-O22
522003040	WP_020514311.1	Actinoplanes globisporus
383779571	YP_005464137.1	Actinoplanes missouriensis 431
522068339	WP_020579548.1	Actinopolymorpha alba
256378738	YP_003102398.1	Actinosynnema mirum DSM 43827
566133066	WP_024003682.1	Advenella kashmirensis
389873672	YP_006381091.1	Advenella kashmirensis WT001
489615574	WP_003520015.1	Agrobacterium
493779563	WP_006727991.1	Agrobacterium albertimagni
15891016	NP_356688.1	Agrobacterium fabrum str. C58
15887747	NP_353428.1	Agrobacterium fabrum str. C58
15890309	NP_355981.1	Agrobacterium fabrum str. C58
222081720	YP_002541085.1	Agrobacterium radiobacter K84
222084751	YP_002543280.1	Agrobacterium radiobacter K84
518396501	WP_019566708.1	Agrobacterium sp. 10MFCol1.1
518853958	WP_020009848.1	Agrobacterium sp. 224MFTsu3.1
493357094	WP_006313644.1	Agrobacterium sp. ATCC 31749
493354009	WP_006310648.1	Agrobacterium sp. ATCC 31749
493355785	WP_006312377.1	Agrobacterium sp. ATCC 31749
325291831	YP_004277695.1	Agrobacterium sp. H13-3
332715521	YP_004442987.1	Agrobacterium sp. H13-3
489600029	WP_003504471.1	Agrobacterium tumefaciens
489589193	WP_003493637.1	Agrobacterium tumefaciens
489610428	WP_003514869.1	Agrobacterium tumefaciens
489599183	WP_003503625.1	Agrobacterium tumefaciens
489597382	WP_003501825.1	Agrobacterium tumefaciens
489595070	WP_003499513.1	Agrobacterium tumefaciens
523692947	WP_020811661.1	Agrobacterium tumefaciens
489617263	WP_003521703.1	Agrobacterium tumefaciens
489601220	WP_003505661.1	Agrobacterium tumefaciens
490566295	WP_004431315.1	Agrobacterium tumefaciens
523692088	WP_020810900.1	Agrobacterium tumefaciens
586948507	AHK00296.1	Agrobacterium tumefaciens
		LBA4213 (Ach5)
586952651	AHK04439.1	Agrobacterium tumefaciens LBA4213 (Ach5)
222102601	YP_002539640.1	Agrobacterium vitis S4
222147413	YP_002548370.1	Agrobacterium vitis S4
222102428	YP_002539467.1	Agrobacterium vitis S4
551267657	WP_022887826.1	Agromyces italicus
551267659	WP_022887828.1	Agromyces italicus
551273816	WP_022893964.1	Agromyces subbeticus
551271315	WP_022891474.1	Agromyces subbeticus
517518279	WP_018688487.1	Ahrensia kielensis
517518274	WP_018688482.1	Ahrensia kielensis
301754455	XP_002913068.1	Ailuropoda melanoleuca
517522927	WP_018693135.1	Algicola sagamiensis
495473773	WP_008198461.1	Algoriphagus machipongonensis
114321207	YP_742890.1	Alkalilimnicola ehrlichii MLHE-1
150388506	YP_001318555.1	Alkaliphilus metalliredigens QYMF
158319092	YP_001511599.1	Alkaliphilus oremlandii OhILAs
564263013	XP_006270376.1	Alligator mississippiensis
494946795	WP_007672823.1	alpha proteobacterium BAL199
495875061	WP_008599640.1	alpha proteobacterium JLT2015
516071880	WP_017502463.1	alpha proteobacterium LLX12A
518900848	WP_020056723.1	alpha proteobacterium SCGC AAA076-C03
518888039	WP_020043914.1	alpha proteobacterium SCGC AAA298-K06
516678596	WP_018037164.1	alpha proteobacterium SCGC AB-629-F11
493845969	WP_006793008.1	Alteromonadales bacterium TW-7
519105454	WP_020261329.1	Aminicenantes bacterium SCGC AAA252-J15
493343267	WP_006300097.1	Aminomonas paucivorans
493345122	WP_006301915.1	Aminomonas paucivorans
340370310	XP_003383689.1	Amphimedon queenslandica
522125145	WP_020636354.1	Amycolatopsis alba
522124337	WP_020635546.1	Amycolatopsis alba
491295884	WP_005153899.1	Amycolatopsis azurea
522135538	WP_020646747.1	Amycolatopsis balhimycina
522147561	WP_020658769.1	Amycolatopsis benzoatilytica
522147569	WP_020658777.1	Amycolatopsis benzoatilytica
494088240	WP_007029075.1	Amycolatopsis decaplanina
300785137	YP_003765428.1	Amycolatopsis mediterranei U32
522157060	WP_020668268.1	Amycolatopsis nigrescens
506932171	YP_008012609.1	Amycolatopsis orientalis HCCB10007
490695345	WP_004559062.1	Amycolatopsis vancoresmycina
392408252	YP_006444860.1	Anaerobaculum mobile DSM 13181
493842604	WP_006789705.1	Anaeroglobus geminatus
327280055	XP_003224770.1	Anolis carolinensis
524892045	XP_005102554.1	Aplysia californica
590079118	EXL05434.1	Aquamicrobium defluvii
590074976	EXL01344.1	Aquamicrobium defluvii

590076045	EXL02385.1	Aquamicrobium defluvii
610625252	EZH71389.1	Aquimarina sp. 22II-S11-z7
610626765	EZH72848.1	Aquimarina sp. 22II-S11-z7
296272750	YP_003655381.1	Arcobacter nitrofigilis DSM 7299
518215928	WP_019386136.1	Arenitalea lutea
517546462	WP_018716670.1	Arhodomonas aquaeolei
517547599	WP_018717807.1	Arhodomonas aquaeolei
403529650	YP_006664389.1	Arthrobacter sp. Rue61a
403529639	YP_006664378.1	Arthrobacter sp. Rue61a
238506185	XP_002384294.1	Aspergillus flavus NRRL3357
238506299	XP_002384351.1	Aspergillus flavus NRRL3357
238485578	XP_002374027.1	Aspergillus flavus NRRL3357
159125934	EDP51050.1	Aspergillus fumigatus A1163
70985272	XP_748142.1	Aspergillus fumigatus Af293
358371851	GAA88457.1	Aspergillus kawachii IFO 4308
358372441	GAA89044.1	Aspergillus kawachii IFO 4308
259487495	CBF86218.1	Aspergillus nidulans FGSC A4
134077107	CAK45448.1	Aspergillus niger
350634127	EHA22490.1	Aspergillus niger ATCC 1015
350637288	EHA25645.1	Aspergillus niger ATCC 1015
350629705	EHA18078.1	Aspergillus niger ATCC 1015
317030994	XP_001392593.2	Aspergillus niger CBS 513.88
145236282	XP_001390789.1	Aspergillus niger CBS 513.88
391873331	EIT82374.1	Aspergillus oryzae 3.042
391864413	EIT73709.1	Aspergillus oryzae 3.042
391873333	EIT82376.1	Aspergillus oryzae 3.042
169785209	XP_001827065.1	Aspergillus oryzae RIB40
83775684	BAE65804.1	Aspergillus oryzae RIB40
317158606	XP_001827113.2	Aspergillus oryzae RIB40
317158247	XP_001826937.2	Aspergillus oryzae RIB40
83775861	BAE65980.1	Aspergillus oryzae RIB40
317144796	XP_003189630.1	Aspergillus oryzae RIB40
599156422	EYE95570.1	Aspergillus ruber CBS 135680
599150499	EYE90047.1	Aspergillus ruber CBS 135680
599156408	EYE95556.1	Aspergillus ruber CBS 135680
516751608	WP_018083033.1	Asticcacaulis benevestitus
493313442	WP_006270875.1	Asticcacaulis biprosthecium
315500377	YP_004089180.1	Asticcacaulis excentricus CB 48
557824517	WP_023452780.1	Asticcacaulis sp. AC460
557833189	WP_023457987.1	Asticcacaulis sp. AC466
597753967	XP_007238803.1	Astyanax mexicanus
496502823	WP_009211106.1	Aurantimonas manganoxydans
518838793	WP_019994683.1	Aureimonas ureilytica
518838795	WP_019994685.1	Aureimonas ureilytica
323454288	EGB10158.1	Aureococcus anophagefferens
490397605	WP_004273902.1	Azospirillum amazonense
565411963	BAO21621.1	Azospirillum brasilense
612166716	EZQ03357.1	Azospirillum brasilense
531034622	BAN78527.1	Azospirillum brasilense
392379308	YP_004986467.1	Azospirillum brasilense Sp245
447183024	WP_001260280.1	Bacillus anthracis
30262803	NP_845180.1	Bacillus anthracis str. Ames

30261035	NP_843412.1	Bacillus anthracis str. Ames
30262802	NP_845179.1	Bacillus anthracis str. Ames
386734730	YP_006207911.1	Bacillus anthracis str. H9401
612488478	AHX17146.1	Bacillus bombysepticus str. Wang
612490200	AHX18868.1	Bacillus bombysepticus str. Wang
447180287	WP_001257543.1	Bacillus cereus
506990671	WP_016081265.1	Bacillus cereus
487983235	WP_002055853.1	Bacillus cereus
447180263	WP_001257519.1	Bacillus cereus
507044065	WP_016115119.1	Bacillus cereus
447180265	WP_001257521.1	Bacillus cereus
507033398	WP_016105097.1	Bacillus cereus
507062085	WP_016132924.1	Bacillus cereus
446764795	WP_000842051.1	Bacillus cereus
513823126	WP_016512683.1	Bacillus cereus
488065364	WP_002136761.1	Bacillus cereus
507051687	WP_016122676.1	Bacillus cereus
447180261	WP_001257517.1	Bacillus cereus
488090091	WP_002161488.1	Bacillus cereus
507029534	WP_016101254.1	Bacillus cereus
447180300	WP_001257556.1	Bacillus cereus
446764792	WP_000842048.1	Bacillus cereus
488114254	WP_002185651.1	Bacillus cereus
446764790	WP_000842046.1	Bacillus cereus
488075934	WP_002147331.1	Bacillus cereus
447180266	WP_001257522.1	Bacillus cereus
447180295	WP_001257551.1	Bacillus cereus
507012007	WP_016087521.1	Bacillus cereus
447180278	WP_001257534.1	Bacillus cereus
447180279	WP_001257535.1	Bacillus cereus
507019432	WP_016091545.1	Bacillus cereus
488094225	WP_002165622.1	Bacillus cereus
447180267	WP_001257523.1	Bacillus cereus
488129018	WP_002200226.1	Bacillus cereus
488100913	WP_002172310.1	Bacillus cereus
507036894	WP_016108577.1	Bacillus cereus
488048603	WP_002120000.1	Bacillus cereus
447180291	WP_001257547.1	Bacillus cereus
488070697	WP_002142094.1	Bacillus cereus
447180274	WP_001257530.1	Bacillus cereus
447180276	WP_001257532.1	Bacillus cereus
488038931	WP_002110328.1	Bacillus cereus
447180273	WP_001257529.1	Bacillus cereus
447180264	WP_001257520.1	Bacillus cereus
447180290	WP_001257546.1	Bacillus cereus
447180292	WP_001257548.1	Bacillus cereus
447183018	WP_001260274.1	Bacillus cereus
487986469	WP_002058932.1	Bacillus cereus
446791405	WP_000868661.1	Bacillus cereus
446626045	WP_000703391.1	Bacillus cereus
507041655	WP_016113311.1	Bacillus cereus
513831477	WP_016513827.1	Bacillus cereus

487934372	WP_002007838.1	Bacillus cereus	446791413	WP_000868669.1	Bacillus cereus
446791423	WP_000868679.1	Bacillus cereus	446791485	WP_000868741.1	Bacillus cereus
487921097	WP_001994563.1	Bacillus cereus	507055703	WP_016126624.1	Bacillus cereus
487964873	WP_002038054.1	Bacillus cereus	446791434	WP_000868690.1	Bacillus cereus
488099219	WP_002170616.1	Bacillus cereus	446791419	WP_000868675.1	Bacillus cereus
488093225	WP_002164622.1	Bacillus cereus	488069162	WP_002140559.1	Bacillus cereus
487925704	WP_001999170.1	Bacillus cereus	487950825	WP_002024291.1	Bacillus cereus
446791505	WP_000868761.1	Bacillus cereus	488043371	WP_002114768.1	Bacillus cereus
506986622	WP_016078048.1	Bacillus cereus	488087693	WP_002159090.1	Bacillus cereus
488078838	WP_002150235.1	Bacillus cereus	507046238	WP_016117275.1	Bacillus cereus
487967632	WP_002040711.1	Bacillus cereus	446791488	WP_000868744.1	Bacillus cereus
446791518	WP_000868774.1	Bacillus cereus	446791403	WP_000868659.1	Bacillus cereus
487919580	WP_001993046.1	Bacillus cereus	446791498	WP_000868754.1	Bacillus cereus
446791493	WP_000868749.1	Bacillus cereus	487896840	WP_001970306.1	Bacillus cereus
446626044	WP_000703390.1	Bacillus cereus	446791483	WP_000868739.1	Bacillus cereus
446791409	WP_000868665.1	Bacillus cereus	446791414	WP_000868670.1	Bacillus cereus
487989268	WP_002061584.1	Bacillus cereus	488015917	WP_002087316.1	Bacillus cereus
446791501	WP_000868757.1	Bacillus cereus	507032170	WP_016103872.1	Bacillus cereus
446791509	WP_000868765.1	Bacillus cereus	446791514	WP_000868770.1	Bacillus cereus
487933559	WP_002007025.1	Bacillus cereus	507016864	WP_016089126.1	Bacillus cereus
507030009	WP_016101723.1	Bacillus cereus	507025788	WP_016097806.1	Bacillus cereus
487910809	WP_001984275.1	Bacillus cereus	507037633	WP_016109315.1	Bacillus cereus
488130394	WP_002201602.1	Bacillus cereus	488054186	WP_002125583.1	Bacillus cereus
487928698	WP_002002164.1	Bacillus cereus	487993634	WP_002065759.1	Bacillus cereus
446791433	WP_000868689.1	Bacillus cereus	446791515	WP_000868771.1	Bacillus cereus
446791429	WP_000868685.1	Bacillus cereus	487945007	WP_002018473.1	Bacillus cereus
488045210	WP_002116607.1	Bacillus cereus	487944162	WP_002017628.1	Bacillus cereus
487926495	WP_001999961.1	Bacillus cereus	487939511	WP_002012977.1	Bacillus cereus
446791407	WP_000868663.1	Bacillus cereus	446791420	WP_000868676.1	Bacillus cereus
488000158	WP_002072035.1	Bacillus cereus	507062926	WP_016133736.1	Bacillus cereus
515087255	WP_016716604.1	Bacillus cereus	507021108	WP_016093220.1	Bacillus cereus
446791494	WP_000868750.1	Bacillus cereus	487948170	WP_002021636.1	Bacillus cereus
446791411	WP_000868667.1	Bacillus cereus	487959250	WP_002032610.1	Bacillus cereus
487953652	WP_002027118.1	Bacillus cereus	487954379	WP_002027844.1	Bacillus cereus
487952463	WP_002025929.1	Bacillus cereus	488097660	WP_002169057.1	Bacillus cereus
487978345	WP_002051129.1	Bacillus cereus	487924190	WP_001997656.1	Bacillus cereus
487931755	WP_002005221.1	Bacillus cereus	487970888	WP_002043811.1	Bacillus cereus
488124427	WP_002195635.1	Bacillus cereus	507065745	WP_016136545.1	Bacillus cereus
507057897	WP_016128803.1	Bacillus cereus	507022589	WP_016094682.1	Bacillus cereus
446791416	WP_000868672.1	Bacillus cereus	487956666	WP_002030067.1	Bacillus cereus
487974490	WP_002047365.1	Bacillus cereus	487980661	WP_002053390.1	Bacillus cereus
488126659	WP_002197867.1	Bacillus cereus	488063258	WP_002134655.1	Bacillus cereus
488002950	WP_002074711.1	Bacillus cereus	446791431	WP_000868687.1	Bacillus cereus
446791512	WP_000868768.1	Bacillus cereus	488094893	WP_002166290.1	Bacillus cereus
446791502	WP_000868758.1	Bacillus cereus	487965708	WP_002038840.1	Bacillus cereus
446791426	WP_000868682.1	Bacillus cereus	487937421	WP_002010887.1	Bacillus cereus
487969784	WP_002042746.1	Bacillus cereus	487902233	WP_001975699.1	Bacillus cereus
487991830	WP_002064014.1	Bacillus cereus	487988477	WP_002060831.1	Bacillus cereus
446791503	WP_000868759.1	Bacillus cereus	487983233	WP_002055851.1	Bacillus cereus
446791481	WP_000868737.1	Bacillus cereus	507062084	WP_016132923.1	Bacillus cereus
446625952	WP_000703298.1	Bacillus cereus	488100912	WP_002172309.1	Bacillus cereus
507050978	WP_016121972.1	Bacillus cereus	507060589	WP_016131482.1	Bacillus cereus
488023312	WP_002094711.1	Bacillus cereus	446948273	WP_001025529.1	Bacillus cereus

507044064	WP_016115118.1	Bacillus cereus
446948274	WP_001025530.1	Bacillus cereus
488129019	WP_002200227.1	Bacillus cereus
488038928	WP_002110325.1	Bacillus cereus
488075935	WP_002147332.1	Bacillus cereus
446948277	WP_001025533.1	Bacillus cereus
446948288	WP_001025544.1	Bacillus cereus
446948272	WP_001025528.1	Bacillus cereus
446948291	WP_001025547.1	Bacillus cereus
507058750	WP_016129656.1	Bacillus cereus
506990672	WP_016081266.1	Bacillus cereus
507029535	WP_016101255.1	Bacillus cereus
507049343	WP_016120361.1	Bacillus cereus
507026610	WP_016098628.1	Bacillus cereus
446948313	WP_001025569.1	Bacillus cereus
515088378	WP_016717711.1	Bacillus cereus
446948308	WP_001025564.1	Bacillus cereus
488048602	WP_002119999.1	Bacillus cereus
487980664	WP_002053393.1	Bacillus cereus
487975465	WP_002048310.1	Bacillus cereus
513823128	WP_016512684.1	Bacillus cereus
487970890	WP_002043813.1	Bacillus cereus
488070696	WP_002142093.1	Bacillus cereus
488090092	WP_002161489.1	Bacillus cereus
487965709	WP_002038841.1	Bacillus cereus
446948320	WP_001025576.1	Bacillus cereus
487945008	WP_002018474.1	Bacillus cereus
488004555	WP_002076248.1	Bacillus cereus
488013656	WP_002085055.1	Bacillus cereus
507023518	WP_016095600.1	Bacillus cereus
446948326	WP_001025582.1	Bacillus cereus
487929474	WP_002002940.1	Bacillus cereus
487924191	WP_001997657.1	Bacillus cereus
446948298	WP_001025554.1	Bacillus cereus
446948306	WP_001025562.1	Bacillus cereus
488000164	WP_002072040.1	Bacillus cereus
507019431	WP_016091544.1	Bacillus cereus
446948314	WP_001025570.1	Bacillus cereus
446948325	WP_001025581.1	Bacillus cereus
506986130	WP_016077556.1	Bacillus cereus
446948299	WP_001025555.1	Bacillus cereus
487939513	WP_002012979.1	Bacillus cereus
487959252	WP_002032612.1	Bacillus cereus
446948315	WP_001025571.1	Bacillus cereus
487950824	WP_002024290.1	Bacillus cereus
488065362	WP_002136759.1	Bacillus cereus
487952462	WP_002025928.1	Bacillus cereus
446948327	WP_001025583.1	Bacillus cereus
507012010	WP_016087522.1	Bacillus cereus
487954387	WP_002027845.1	Bacillus cereus
487993635	WP_002065760.1	Bacillus cereus
488126658	WP_002197866.1	Bacillus cereus
507038458	WP_016110140.1	Bacillus cereus
507063902	WP_016134712.1	Bacillus cereus
487902231	WP_001975697.1	Bacillus cereus
487989269	WP_002061585.1	Bacillus cereus
446948317	WP_001025573.1	Bacillus cereus
446948297	WP_001025553.1	Bacillus cereus
446948318	WP_001025574.1	Bacillus cereus
487934371	WP_002007837.1	Bacillus cereus
487948171	WP_002021637.1	Bacillus cereus
487926494	WP_001999960.1	Bacillus cereus
225864808	YP_002750186.1	Bacillus cereus 03BB102
225862863	YP_002748241.1	Bacillus cereus 03BB102
225864807	YP_002750185.1	Bacillus cereus 03BB102
217960275	YP_002338835.1	Bacillus cereus AH187
217958504	YP_002337052.1	Bacillus cereus AH187
217960274	YP_002338834.1	Bacillus cereus AH187
218903956	YP_002451790.1	Bacillus cereus AH820
218902107	YP_002449941.1	Bacillus cereus AH820
218903955	YP_002451789.1	Bacillus cereus AH820
42780070	NP_977317.1	Bacillus cereus ATCC 10987
42781920	NP_979167.1	Bacillus cereus ATCC 10987
42781919	NP_979166.1	Bacillus cereus ATCC 10987
30020954	NP_832585.1	Bacillus cereus ATCC 14579
30019060	NP_830691.1	Bacillus cereus ATCC 14579
30020953	NP_832584.1	Bacillus cereus ATCC 14579
218232893	YP_002367554.1	Bacillus cereus B4264
218232621	YP_002365676.1	Bacillus cereus B4264
218233634	YP_002367553.1	Bacillus cereus B4264
301054356	YP_003792567.1	Bacillus cereus biovar anthracis str. CI
301052550	YP_003790761.1	Bacillus cereus biovar anthracis str. CI
301054355	YP_003792566.1	Bacillus cereus biovar anthracis str. CI
52142682	YP_084148.1	Bacillus cereus E33L
52144422	YP_082406.1	Bacillus cereus E33L
52142681	YP_084147.1	Bacillus cereus E33L
376266697	YP_005119409.1	Bacillus cereus F837/76
402556956	YP_006598227.1	Bacillus cereus FRI-35
402553581	YP_006594852.1	Bacillus cereus FRI-35
402556957	YP_006598228.1	Bacillus cereus FRI-35
218897859	YP_002446270.1	Bacillus cereus G9842
218895938	YP_002444349.1	Bacillus cereus G9842
218897858	YP_002446269.1	Bacillus cereus G9842
487963172	WP_002036436.1	Bacillus cereus group
487968250	WP_002041290.1	Bacillus cereus group
446791511	WP_000868767.1	Bacillus cereus group
446791417	WP_000868673.1	Bacillus cereus group
487929472	WP_002002938.1	Bacillus cereus group
488004556	WP_002076249.1	Bacillus cereus group
487963173	WP_002036437.1	Bacillus cereus group
222094650	YP_002528710.1	Bacillus cereus Q1
222096333	YP_002530390.1	Bacillus cereus Q1
518223348	WP_019393556.1	Bacillus endophyticus
518223347	WP_019393555.1	Bacillus endophyticus

517536735	WP_018706943.1	Bacillus fordii
517536736	WP_018706944.1	Bacillus fordii
518071528	WP_019241736.1	Bacillus massilioanorexius
518071534	WP_019241742.1	Bacillus massilioanorexius
489288018	WP_003195581.1	Bacillus mycoides
489302805	WP_003210262.1	Bacillus mycoides
489290531	WP_003198076.1	Bacillus mycoides
489299872	WP_003207346.1	Bacillus mycoides
489279973	WP_003187619.1	Bacillus mycoides
489299874	WP_003207348.1	Bacillus mycoides
489290533	WP_003198078.1	Bacillus mycoides
489282158	WP_003189780.1	Bacillus mycoides
518213100	WP_019383308.1	Bacillus oceanisediminis
518213099	WP_019383307.1	Bacillus oceanisediminis
493016099	WP_006093712.1	Bacillus pseudomycoides
493019249	WP_006095057.1	Bacillus pseudomycoides
493019247	WP_006095056.1	Bacillus pseudomycoides
517595458	WP_018765666.1	Bacillus sp. 105MF
517596888	WP_018767096.1	Bacillus sp. 105MF
517595457	WP_018765665.1	Bacillus sp. 105MF
518904221	WP_020060096.1	Bacillus sp. 123MFChir2
518906322	WP_020062197.1	Bacillus sp. 123MFChir2
518904222	WP_020060097.1	Bacillus sp. 123MFChir2
496690290	WP_009331833.1	Bacillus sp. 2_A_57_CT2
496690289	WP_009331832.1	Bacillus sp. 2_A_57_CT2
446764794	WP_000842050.1	Bacillus sp. 7_6_55CFAA_CT2
446791412	WP_000868668.1	Bacillus sp. 7_6_55CFAA_CT2
446948310	WP_001025566.1	Bacillus sp. 7_6_55CFAA_CT2
517610430	WP_018780638.1	Bacillus sp. 95MFCvi2.1
517612732	WP_018782940.1	Bacillus sp. 95MFCvi2.1
517610429	WP_018780637.1	Bacillus sp. 95MFCvi2.1
495452331	WP_008177025.1	Bacillus sp. B14905
493977600	WP_006920611.1	Bacillus sp. GeD10
493977601	WP_006920612.1	Bacillus sp. GeD10
516338807	WP_017728840.1	Bacillus sp. L1(2012)
516129580	WP_017560160.1	Bacillus sp. WBUNB001
516129894	WP_017560474.1	Bacillus sp. WBUNB001
516269298	WP_017673261.1	Bacillus sp. WBUNB004
516270516	WP_017674479.1	Bacillus sp. WBUNB004
516269299	WP_017673262.1	Bacillus sp. WBUNB004
516252935	WP_017656898.1	Bacillus sp. WBUNB009
446948311	WP_001025567.1	Bacillus sp. WBUNB009
489333269	WP_003240511.1	Bacillus subtilis
489400802	WP_003307202.1	Bacillus thuringiensis
489402787	WP_003309134.1	Bacillus thuringiensis
446791506	WP_000868762.1	Bacillus thuringiensis
489367635	WP_003274464.1	Bacillus thuringiensis
516372802	WP_017762835.1	Bacillus thuringiensis
489380453	WP_003287145.1	Bacillus thuringiensis
489371853	WP_003278635.1	Bacillus thuringiensis
490540505	WP_004405641.1	Bacillus thuringiensis
489399984	WP_003306403.1	Bacillus thuringiensis
489376625	WP_003283354.1	Bacillus thuringiensis

489390280	WP_003296845.1	Bacillus thuringiensis
489395032	WP_003301541.1	Bacillus thuringiensis
489360017	WP_003266965.1	Bacillus thuringiensis
489393647	WP_003300170.1	Bacillus thuringiensis
516373224	WP_017763257.1	Bacillus thuringiensis
489387713	WP_003294312.1	Bacillus thuringiensis
489400801	WP_003307201.1	Bacillus thuringiensis
489398639	WP_003305093.1	Bacillus thuringiensis
489403253	WP_003309588.1	Bacillus thuringiensis
489363914	WP_003270793.1	Bacillus thuringiensis
489371854	WP_003278636.1	Bacillus thuringiensis
489395034	WP_003301543.1	Bacillus thuringiensis
489360018	WP_003266966.1	Bacillus thuringiensis
490540502	WP_004405638.1	Bacillus thuringiensis
489377808	WP_003284524.1	Bacillus thuringiensis
296503369	YP_003665069.1	Bacillus thuringiensis BMB171
296501621	YP_003663321.1	Bacillus thuringiensis BMB171
296503368	YP_003665068.1	Bacillus thuringiensis BMB171
410675203	YP_006927574.1	Bacillus thuringiensis Bt407
589236709	CDN36294.1	Bacillus thuringiensis DB27
589238100	CDN34292.1	Bacillus thuringiensis DB27
402559839	YP_006602563.1	Bacillus thuringiensis HD-771
402562100	YP_006604824.1	Bacillus thuringiensis HD-771
402559840	YP_006602564.1	Bacillus thuringiensis HD-771
434375812	YP_006610456.1	Bacillus thuringiensis HD-789
434373917	YP_006608561.1	Bacillus thuringiensis HD-789
434375811	YP_006610455.1	Bacillus thuringiensis HD-789
407705259	YP_006828844.1	Bacillus thuringiensis MC28
407703397	YP_006826982.1	Bacillus thuringiensis MC28
407705258	YP_006828843.1	Bacillus thuringiensis MC28
384184946	YP_005570842.1	Bacillus thuringiensis serovar chinensis CT-43
384186897	YP_005572793.1	Bacillus thuringiensis serovar chinensis CT-43
384186896	YP_005572792.1	Bacillus thuringiensis serovar chinensis CT-43
384180721	YP_005566483.1	Bacillus thuringiensis serovar finitimus YBT-020
384178858	YP_005564620.1	Bacillus thuringiensis serovar finitimus YBT-020
384180720	YP_005566482.1	Bacillus thuringiensis serovar finitimus YBT-020
49476772	YP_036919.1	Bacillus thuringiensis serovar konkukian str. 97-27
49480213	YP_035142.1	Bacillus thuringiensis serovar konkukian str. 97-27
49479615	YP_036918.1	Bacillus thuringiensis serovar konkukian str. 97-27
449087682	YP_007420123.1	Bacillus thuringiensis serovar kurstaki str. HD73
449089802	YP_007422243.1	Bacillus thuringiensis serovar kurstaki str. HD73
118478186	YP_895337.1	Bacillus thuringiensis str. Al Hakam
118476542	YP_893693.1	Bacillus thuringiensis str. Al Hakam
118478185	YP_895336.1	Bacillus thuringiensis str. Al Hakam
558681348	YP_008819477.1	Bacillus thuringiensis YBT-1518

558679366	YP_008817495.1	Bacillus thuringiensis YBT-1518
558681347	YP_008819476.1	Bacillus thuringiensis YBT-1518
557619685	YP_008780816.1	Bacillus toyonensis BCT-7112
557619684	YP_008780815.1	Bacillus toyonensis BCT-7112
573585083	ETT83354.1	Bacillus weihenstephanensis FSL H7-687
573584489	ETT82766.1	Bacillus weihenstephanensis FSL R5-860
163940583	YP_001645467.1	Bacillus weihenstephanensis KBAB4
163938804	YP_001643688.1	Bacillus weihenstephanensis KBAB4
163940582	YP_001645466.1	Bacillus weihenstephanensis KBAB4
569022459	GAE41889.1	Bacillus weihenstephanensis NBRC 101238 = DSM 11821
594691318	XP_007192998.1	Balaenoptera acutorostrata scammoni
400593642	EJP61568.1	Beauveria bassiana ARSEF 2860
390942363	YP_006406124.1	Belliella baltica DSM 15883
516078634	WP_017509217.1	beta proteobacterium L13
495573414	WP_008297993.1	Bhargavaea cecembensis
495573413	WP_008297992.1	Bhargavaea cecembensis
470203393	YP_007594105.1	Bifidobacterium thermophilum RBL67
496774940	WP_009368752.1	Bilophila sp. 4_1_30
491165907	WP_005024278.1	Bilophila wadsworthia
491169193	WP_005027563.1	Bilophila wadsworthia
452000198	EMD92660.1	Bipolaris maydis C5
576935531	EUC49034.1	Bipolaris oryzae ATCC 44560
451854282	EMD67575.1	Bipolaris sorokiniana ND90Pr
578485147	EUN22650.1	Bipolaris victoriae FI3
576914960	EUC29284.1	Bipolaris zeicola 26-R-13
516268367	WP_017672330.1	Blastomonas sp. AAP53
564012750	WP_023838164.1	Blastomonas sp. CACIA14H2
488730604	WP_002654021.1	Blastopirellula marina
492750836	WP_005948987.1	Blautia hydrogenotrophica
546357443	WP_021844514.1	Blautia hydrogenotrophica CAG:147
163855568	YP_001629866.1	Bordetella petrii DSM 12804
518780848	WP_019938137.1	Bordetella sp. FB-8
440900862	ELR51903.1	Bos mutus
555980135	XP_005901995.1	Bos mutus
77735719	NP_001029558.1	Bos taurus
257070253	YP_003156508.1	Brachybacterium faecium DSM 4810
257067669	YP_003153924.1	Brachybacterium faecium DSM 4810
586886103	EWS80354.1	Brachybacterium phenoliresistens
608609907	GAJ37780.1	Bradyrhizobium sp. DOA9
494874324	WP_007600419.1	Bradyrhizobium sp. WSM1253
261289669	XP_002604811.1	Branchiostoma floridae
261289665	XP_002604809.1	Branchiostoma floridae
261289667	XP_002604810.1	Branchiostoma floridae
261289671	XP_002604812.1	Branchiostoma floridae
261289663	XP_002604808.1	Branchiostoma floridae
492406589	WP_005834650.1	Brevibacillus agri
492406579	WP_005834644.1	Brevibacillus agri
515112335	WP_016741395.1	Brevibacillus brevis
515821443	WP_017252196.1	Brevibacillus brevis
515821440	WP_017252193.1	Brevibacillus brevis
515112332	WP_016741392.1	Brevibacillus brevis
226314148	YP_002774044.1	Brevibacillus brevis NBRC 100599
226314145	YP_002774041.1	Brevibacillus brevis NBRC 100599
494989837	WP_007715857.1	Brevibacillus sp. BC25
494989834	WP_007715854.1	Brevibacillus sp. BC25
495062684	WP_007787514.1	Brevibacillus sp. CF112
495062680	WP_007787510.1	Brevibacillus sp. CF112
496830742	WP_009377281.1	Brevibacterium casei
496829089	WP_009376699.1	Brevibacterium casei
497568374	WP_009882558.1	Brevibacterium linens
497570303	WP_009884487.1	Brevibacterium linens
489256635	WP_003164570.1	Brevundimonas diminuta
489262002	WP_003169865.1	Brevundimonas diminuta
489257294	WP_003165218.1	Brevundimonas diminuta
493169055	WP_006172804.1	Brucella
496221300	WP_008935335.1	Brucella
493009457	WP_006088903.1	Brucella abortus
493088738	WP_006129376.1	Brucella abortus
514962411	WP_016650778.1	Brucella abortus
489053312	WP_002963502.1	Brucella abortus
493015257	WP_006093304.1	Brucella abortus
62290658	YP_222451.1	Brucella abortus bv. 1 str. 9-941
161618320	YP_001592207.1	Brucella canis ATCC 23365
560147661	YP_008840477.1	Brucella ceti TE10759-12
495782910	WP_008507489.1	Brucella inopinata
495783426	WP_008508005.1	Brucella inopinata
490823760	WP_004685850.1	Brucella melitensis
490823344	WP_004685434.1	Brucella melitensis
493306368	WP_006263910.1	Brucella melitensis
493107164	WP_006139538.1	Brucella melitensis
493104394	WP_006137744.1	Brucella melitensis
493130658	WP_006151420.1	Brucella melitensis
225853238	YP_002733471.1	Brucella melitensis ATCC 23457
17986541	NP_539175.1	Brucella melitensis bv. 1 str. 16M
17987869	NP_540503.1	Brucella melitensis bv. 1 str. 16M
256368795	YP_003106301.1	Brucella microti CCM 4915
490826627	WP_004688717.1	Brucella neotomae
490825904	WP_004687994.1	Brucella neotomae
148560609	YP_001258374.1	Brucella ovis ATCC 25840
493691256	WP_006641268.1	Brucella pinnipedialis
493153062	WP_006163367.1	Brucella sp. 63/311
496764367	WP_009364131.1	Brucella sp. BO2
496765822	WP_009364714.1	Brucella sp. BO2
496219733	WP_008933770.1	Brucella sp. NVSL 07-0026

493209501	WP_006195325.1	Brucella suis
23502646	NP_698773.1	Brucella suis 1330
23501244	NP_697371.1	Brucella suis 1330
163845368	YP_001623023.1	Brucella suis ATCC 23445
594092748	XP_006069721.1	Bubalus bubalis
493807600	WP_006755369.1	Burkholderia ambifaria
493814447	WP_006762086.1	Burkholderia ambifaria
493814222	WP_006761867.1	Burkholderia ambifaria
493807101	WP_006754886.1	Burkholderia ambifaria
115358519	YP_775657.1	Burkholderia ambifaria AMMD
115358300	YP_775438.1	Burkholderia ambifaria AMMD
172063272	YP_001810923.1	Burkholderia ambifaria MC40-6
172062266	YP_001809917.1	Burkholderia ambifaria MC40-6
172062263	YP_001809914.1	Burkholderia ambifaria MC40-6
172063061	YP_001810712.1	Burkholderia ambifaria MC40-6
518910341	WP_020066216.1	Burkholderia bryophila
575856661	ETY75950.1	Burkholderia caribensis MBA4
575857956	ETY77206.1	Burkholderia caribensis MBA4
575860652	ETY79866.1	Burkholderia caribensis MBA4
493541013	WP_006494886.1	Burkholderia cenocepacia
493525007	WP_006479059.1	Burkholderia cenocepacia
493541184	WP_006495057.1	Burkholderia cenocepacia
107026358	YP_623869.1	Burkholderia cenocepacia AU 1054
107026574	YP_624085.1	Burkholderia cenocepacia AU 1054
206563338	YP_002234101.1	Burkholderia cenocepacia J2315
206563118	YP_002233881.1	Burkholderia cenocepacia J2315
170738345	YP_001779605.1	Burkholderia cenocepacia MC0-3
170735763	YP_001777023.1	Burkholderia cenocepacia MC0-3
402569012	YP_006618356.1	Burkholderia cepacia GG4
402569453	YP_006618797.1	Burkholderia cepacia GG4
402569397	YP_006618741.1	Burkholderia cepacia GG4
402569217	YP_006618561.1	Burkholderia cepacia GG4
571036043	ETP62334.1	Burkholderia dolosa PC543
330819785	YP_004348647.1	Burkholderia gladioli BSR3
330819676	YP_004348538.1	Burkholderia gladioli BSR3
238023840	YP_002908072.1	Burkholderia glumae BGR1
492939797	WP_006052729.1	Burkholderia graminis
516385758	WP_017775434.1	Burkholderia kururiensis
78062510	YP_372418.1	Burkholderia lata
78062744	YP_372652.1	Burkholderia lata
53716979	YP_106025.1	Burkholderia mallei ATCC 23344
126446932	YP_001078080.1	Burkholderia mallei NCTC 10247
493460017	WP_006415254.1	Burkholderia multivorans
493441664	WP_006397095.1	Burkholderia multivorans
493449232	WP_006404539.1	Burkholderia multivorans
493449235	WP_006404542.1	Burkholderia multivorans
493451508	WP_006406785.1	Burkholderia multivorans
493461493	WP_006416713.1	Burkholderia multivorans
493448462	WP_006403784.1	Burkholderia multivorans
493444474	WP_006399858.1	Burkholderia multivorans
493456522	WP_006411786.1	Burkholderia multivorans
161520806	YP_001584233.1	Burkholderia multivorans ATCC 17616
161521800	YP_001585227.1	Burkholderia multivorans ATCC 17616
161521803	YP_001585230.1	Burkholderia multivorans ATCC 17616
189352828	YP_001948455.1	Burkholderia multivorans ATCC 17616
161520992	YP_001584419.1	Burkholderia multivorans ATCC 17616
497796801	WP_010110985.1	Burkholderia oklahomensis
407711417	YP_006836190.1	Burkholderia phenoliruptrix BR3459a
407709279	YP_006793143.1	Burkholderia phenoliruptrix BR3459a
186472203	YP_001859545.1	Burkholderia phymatum STM815
186473481	YP_001860823.1	Burkholderia phymatum STM815
187920379	YP_001889410.1	Burkholderia phytofirmans PsJN
187919272	YP_001888303.1	Burkholderia phytofirmans PsJN
497618643	WP_009932827.1	Burkholderia pseudomallei
76817590	YP_337044.1	Burkholderia pseudomallei 1710b
53721367	YP_110352.1	Burkholderia pseudomallei K96243
515898935	WP_017329518.1	Burkholderia pyrrocinia
515899147	WP_017329730.1	Burkholderia pyrrocinia
495024721	WP_007750493.1	Burkholderia sp. BT03
495008421	WP_007734433.1	Burkholderia sp. BT03
495004183	WP_007730197.1	Burkholderia sp. BT03
495019364	WP_007745261.1	Burkholderia sp. BT03
323528430	YP_004230582.1	Burkholderia sp. CCGE1001
295699712	YP_003607605.1	Burkholderia sp. CCGE1002
295699990	YP_003607883.1	Burkholderia sp. CCGE1002
307727295	YP_003910508.1	Burkholderia sp. CCGE1003
494317733	WP_007177657.1	Burkholderia sp. Ch1-1
494316885	WP_007177278.1	Burkholderia sp. Ch1-1
494318228	WP_007177867.1	Burkholderia sp. Ch1-1
496200934	WP_008920771.1	Burkholderia sp. H160
496204248	WP_008924083.1	Burkholderia sp. H160
517249407	WP_018438225.1	Burkholderia sp. JPY251
517245407	WP_018434225.1	Burkholderia sp. JPY251
522054830	WP_020566039.1	Burkholderia sp. JPY347
387905272	YP_006335610.1	Burkholderia sp. KJ006
507518235	YP_008039851.1	Burkholderia sp. RPE64
507518401	YP_008040016.1	Burkholderia sp. RPE64
507526191	YP_008047814.1	Burkholderia sp. RPE64
495622730	WP_008347309.1	Burkholderia sp. SJ98
495622724	WP_008347303.1	Burkholderia sp. SJ98
495628986	WP_008353565.1	Burkholderia sp. SJ98
325520013	EGC99246.1	Burkholderia sp. TJI49
325519646	EGC98985.1	Burkholderia sp. TJI49

517229036	WP_018417854.1	Burkholderia sp. WSM4176
517236508	WP_018425326.1	Burkholderia sp. WSM4176
517234606	WP_018423424.1	Burkholderia sp. WSM4176
377811535	YP_005043975.1	Burkholderia sp. YI23
377811886	YP_005044326.1	Burkholderia sp. YI23
377807950	YP_004979142.1	Burkholderia sp. YI23
377807952	YP_004979144.1	Burkholderia sp. YI23
377807727	YP_004978919.1	Burkholderia sp. YI23
377808695	YP_004979887.1	Burkholderia sp. YI23
377808233	YP_004979425.1	Burkholderia sp. YI23
377813016	YP_005042265.1	Burkholderia sp. YI23
494851793	WP_007577893.1	Burkholderia terrae
494862312	WP_007588412.1	Burkholderia terrae
494861424	WP_007587524.1	Burkholderia terrae
497593870	WP_009908054.1	Burkholderia thailandensis
497587058	WP_009901242.1	Burkholderia thailandensis
492898578	WP_006028984.1	Burkholderia thailandensis
83718189	YP_440255.1	Burkholderia thailandensis E264
488605793	YP_007920850.1	Burkholderia thailandensis MSMB121
497784460	WP_010098644.1	Burkholderia ubonensis
497775870	WP_010090054.1	Burkholderia ubonensis
544729350	WP_021159162.1	Burkholderia vietnamiensis
544726804	WP_021156666.1	Burkholderia vietnamiensis
134293388	YP_001117124.1	Burkholderia vietnamiensis G4
91779072	YP_554280.1	Burkholderia xenovorans LB400
91778687	YP_553895.1	Burkholderia xenovorans LB400
91779222	YP_554430.1	Burkholderia xenovorans LB400
557726988	GAD94392.1	Byssochlamys spectabilis No. 5
557726188	GAD95183.1	Byssochlamys spectabilis No. 5
296215168	XP_002807287.1	Callithrix jacchus
560919099	XP_006185773.1	Camelus ferus
354545171	CCE41897.1	Candida parapsilosis
494804704	WP_007540112.1	Candidatus Aquiluna sp. IMCC13023
494056614	WP_006998705.1	Candidatus Burkholderia kirkii
575412461	ETW98196.1	Candidatus Entotheonella sp. TSY1
575417260	ETX02176.1	Candidatus Entotheonella sp. TSY2
170290417	YP_001737233.1	Candidatus Korarchaeum cryptofilum OPF8
294083884	YP_003550641.1	Candidatus Puniceispirillum marinum IMCC1322
294083883	YP_003550640.1	Candidatus Puniceispirillum marinum IMCC1322
116625630	YP_827786.1	Candidatus Solibacter usitatus Ellin6076
57090613	XP_547837.1	Canis lupus familiaris
443731097	ELU16335.1	Capitella teleta
548483764	XP_005685977.1	Capra hircus
590018467	EXJ93666.1	Capronia coronata CBS 617.96
590006647	EXJ81856.1	Capronia coronata CBS 617.96
590016467	EXJ91667.1	Capronia epimyces CBS 606.96
590009418	EXJ84624.1	Capronia epimyces CBS 606.96
589267939	CDM25875.1	Castellaniella defragrans 65Phen
522008485	WP_020519756.1	Catelliglobosispora koreensis
256395050	YP_003116614.1	Catenulispora acidiphila DSM 44928
516719023	WP_018062604.1	Caulobacter
348573264	XP_003472411.1	Cavia porcellus
496477013	WP_009185774.1	Cecembia lonarensis
319955709	YP_004166976.1	Cellulophaga algicola DSM 14237
584428683	EWH13359.1	Cellulophaga geojensis KL-A
325288132	YP_004263922.1	Cellulophaga lytica DSM 7489
478503920	XP_004426262.1	Ceratotherium simum simum
449546484	EMD37453.1	Ceriporiopsis subvermispora B
110633939	YP_674147.1	Chelativorans sp. BNC1
518232762	WP_019402970.1	Chelatococcus sp. GW1
533171622	XP_005399284.1	Chinchilla lanigera
256424547	YP_003125200.1	Chitinophaga pinensis DSM 2588
163848508	YP_001636552.1	Chloroflexus aurantiacus J-10-fl
34498281	NP_902496.1	Chromobacterium violaceum ATCC 12472
92114823	YP_574751.1	Chromohalobacter salexigens DSM 3043
92114460	YP_574388.1	Chromohalobacter salexigens DSM 3043
530584374	XP_005285995.1	Chrysemys picta bellii
489069390	WP_002979369.1	Chryseobacterium gleum
573452204	GAE65221.1	Chryseobacterium indologenes NBRC 14944
515973427	WP_017404010.1	Chryseobacterium taeanense
586468102	XP_006864657.1	Chrysochloris asiatica
494037191	WP_006979317.1	Chthoniobacter flavus
198422372	XP_002129543.1	Ciona intestinalis
198412596	XP_002124652.1	Ciona intestinalis
489926777	WP_003830113.1	Citrobacter freundii
575565961	ETX65600.1	Citrobacter freundii UCI 32
283786214	YP_003366079.1	Citrobacter rodentium ICC168
496061569	WP_008786076.1	Citrobacter sp. 30_2
565935309	ETI24538.1	Cladophialophora carrionii CBS 160.54
589992107	EXJ74689.1	Cladophialophora psammophila CBS 110553
589975512	EXJ58813.1	Cladophialophora yegresii CBS 114405
589977331	EXJ60615.1	Cladophialophora yegresii CBS 114405
148271990	YP_001221551.1	Clavibacter michiganensis subsp. michiganensis NCPPB 382
473832772	YP_007685070.1	Clavibacter michiganensis subsp. nebraskensis NCPPB 2581
170780526	YP_001708858.1	Clavibacter michiganensis subsp. sepedonicus
544699021	WP_021130144.1	Clostridium
545061010	WP_021434104.1	Clostridium

545057010	WP_021430157.1	Clostridium
517421282	WP_018592765.1	Clostridium
493484901	WP_006439715.1	Clostridium
310659532	YP_003937253.1	Clostridium
544695260	WP_021126550.1	Clostridium
6900003	CAB71312.1	Clostridium
490751284	WP_004613592.1	Clostridium
490576384	WP_004441404.1	Clostridium botulinum
544705636	WP_021136255.1	Clostridium botulinum
148380430	YP_001254971.1	Clostridium botulinum A str. ATCC 3502
559781388	GAE02922.1	Clostridium botulinum B str. Osaka05
237795912	YP_002863464.1	Clostridium botulinum Ba4 str. 657
494122896	WP_007062673.1	Clostridium carboxidivorans
494122905	WP_007062682.1	Clostridium carboxidivorans
495138414	WP_007863221.1	Clostridium citroniae
488671168	WP_002599959.1	Clostridium hathewayi
493490252	WP_006444975.1	Clostridium hylemonae
490745003	WP_004607311.1	Clostridium scindens
497980698	WP_010294854.1	Clostridium senegalense
547958439	WP_022358812.1	Clostridium sp. CAG:43
496289811	WP_009001849.1	Clostridium sp. D5
496460564	WP_009169409.1	Clostridium sp. DL-VIII
545398543	WP_021638234.1	Clostridium sp. KLE 1755
516361638	WP_017751671.1	Clostridium tyrobutyricum
588898121	EXF79245.1	Colletotrichum fioriniae PJ7
588902099	EXF82477.1	Colletotrichum fioriniae PJ7
588905783	EXF85472.1	Colletotrichum fioriniae PJ7
588904662	EXF84549.1	Colletotrichum fioriniae PJ7
530479791	EQB58914.1	Colletotrichum gloeosporioides Cg-14
530479669	EQB58816.1	Colletotrichum gloeosporioides Cg-14
530474688	EQB54879.1	Colletotrichum gloeosporioides Cg-14
530462298	EQB45234.1	Colletotrichum gloeosporioides Cg-14
596666583	XP_007276135.1	Colletotrichum gloeosporioides Nara gc5
596678444	XP_007278372.1	Colletotrichum gloeosporioides Nara gc5
596718578	XP_007285769.1	Colletotrichum gloeosporioides Nara gc5
596710356	XP_007284291.1	Colletotrichum gloeosporioides Nara gc5
310800425	EFQ35318.1	Colletotrichum graminicola M1.001
380491410	CCF35343.1	Colletotrichum higginsianum
477535076	ENH86604.1	Colletotrichum orbiculare MAFF 240422
477533226	ENH84816.1	Colletotrichum orbiculare MAFF 240422
340788726	YP_004754191.1	Collimonas fungivorans Ter331
543730802	XP_005506452.1	Columba livia
449278496	EMC86318.1	Columba livia
517856755	WP_019026963.1	Colwellia piezophila
517856758	WP_019026966.1	Colwellia piezophila

71280867	YP_268192.1	Colwellia psychrerythraea 34H
71277747	YP_268195.1	Colwellia psychrerythraea 34H
507945413	XP_004681880.1	Condylura cristata
494825404	EON62590.1	Coniosporium apollinis CBS 100218
495647913	WP_008372492.1	Coprococcus comes
547811030	WP_022220198.1	Coprococcus comes CAG:19
517108380	WP_018297198.1	Corynebacterium lubricantis
405958634	EKC24743.1	Crassostrea gigas
405950622	EKC18597.1	Crassostrea gigas
354501541	XP_003512849.1	Cricetulus griseus
344249669	EGW05773.1	Cricetulus griseus
493146288	WP_006159973.1	Cupriavidus basilensis
493142280	WP_006157872.1	Cupriavidus basilensis
601455033	EYT01341.1	Cupriavidus sp. SK-3
568122136	ETN44731.1	Cyphellophora europaea CBS 101466
551205267	WP_022833317.1	Cytophagales str. B6
68391886	XP_694147.1	Danio rerio
488521224	XP_004452534.1	Dasypus novemcinctus
291280010	YP_003496845.1	Deferribacter desulfuricans SSM1
226357446	YP_002787185.1	Deinococcus deserti VCD115
516904165	WP_018157906.1	Demetria terragena
224371801	YP_002605965.1	Desulfobacterium autotrophicum HRM2
224370569	YP_002604733.1	Desulfobacterium autotrophicum HRM2
550912207	WP_022665217.1	Desulfospira joergensenii
550913254	WP_022666252.1	Desulfospira joergensenii
392424548	YP_006465542.1	Desulfosporosinus acidiphilus SJ4
497309999	WP_009624216.1	Desulfosporosinus sp. OT
494023180	WP_006965434.1	Desulfotignum phosphitoxidans
494025489	WP_006967703.1	Desulfotignum phosphitoxidans
220904954	YP_002480266.1	Desulfovibrio desulfuricans subsp. desulfuricans str. ATCC 27774
550908369	WP_022661474.1	Desulfovibrio longus
597974293	XP_007361675.1	Dichomitus squalens LYAD-421 SS1
494903361	WP_007629406.1	Dietzia cinnamea
85818225	EAQ39385.1	Dokdonia donghaensis MED134
493472321	WP_006427363.1	Dorea longicatena
548195963	WP_022416476.1	Dorea longicatena CAG:42
510885559	WP_016220098.1	Dorea sp. 5-2
546428562	WP_021859784.1	Dorea sp. CAG:105
547916271	WP_022319005.1	Dorea sp. CAG:317
452843265	EME45200.1	Dothistroma septosporum NZE10
518767344	WP_019924633.1	Duganella zoogloeoides
518784378	WP_019941667.1	Dyadobacter beijingensis
255038019	YP_003088640.1	Dyadobacter fermentans DSM 18053
518297193	WP_019467401.1	Dyella japonica
518294007	WP_019464215.1	Dyella japonica
612504296	AHX14134.1	Dyella jiangningensis

517285757	WP_018474575.1	Echinicola pacifica
431799267	YP_007226171.1	Echinicola vietnamensis DSM 17526
507629322	XP_004698679.1	Echinops telfairi
585680807	XP_006891834.1	Elephantulus edwardii
496379655	WP_009088645.1	Elizabethkingia anophelis
609710570	CDN80243.1	Elizabethkingia anophelis
544940425	WP_021348755.1	Elizabethkingia meningoseptica
551555851	XP_005765085.1	Emiliania huxleyi CCMP1516
408674880	YP_006874628.1	Emticicia oligotrophica DSM 17448
589089477	AHK46232.1	Ensifer adhaerens OV14
589090302	AHK47055.1	Ensifer adhaerens OV14
589088852	AHK45607.1	Ensifer adhaerens OV14
518923817	WP_020079692.1	Enterobacter aerogenes
444353313	YP_007389457.1	Enterobacter aerogenes EA1509E
336248562	YP_004592272.1	Enterobacter aerogenes KCTC 2190
345297720	YP_004827078.1	Enterobacter asburiae LF7a
311281085	YP_003943316.1	Enterobacter lignolyticus SCF1
510804130	WP_016178899.1	Enterococcus avium
537093523	ERE50372.1	Enterococcus gallinarum EGD-AAK12
498474540	WP_010778865.1	Enterococcus gilvus
498476948	WP_010781267.1	Enterococcus gilvus
392989503	YP_006488096.1	Enterococcus hirae ATCC 9790
498435912	WP_010741592.1	Enterococcus malodoratus
498434013	WP_010739705.1	Enterococcus malodoratus
498436385	WP_010742063.1	Enterococcus malodoratus
498452247	WP_010757752.1	Enterococcus pallens
498452150	WP_010757655.1	Enterococcus pallens
498438345	WP_010744008.1	Enterococcus raffinosus
545203003	XP_005605579.1	Equus caballus
490272062	WP_004168187.1	Erwinia amylovora
312173214	CBX81469.1	Erwinia amylovora ATCC BAA-2158
292489059	YP_003531946.1	Erwinia amylovora CFBP1430
387870478	YP_005801848.1	Erwinia pyrifoliae DSM 12163
259907707	YP_002648063.1	Erwinia pyrifoliae Ep1/96
385785513	YP_005816622.1	Erwinia sp. Ejp617
446480551	WP_000558405.1	Escherichia coli
446480550	WP_000558404.1	Escherichia coli
486168698	WP_001531993.1	Escherichia coli
485733760	WP_001361167.1	Escherichia coli
310825669	YP_003958026.1	Eubacterium limosum KIST612
518500342	WP_019670549.1	Eudoraea adriatica
378728459	EHY54918.1	Exophiala dermatitidis NIH/UT8656
378728343	EHY54802.1	Exophiala dermatitidis NIH/UT8656
529419229	XP_005229480.1	Falco peregrinus
410962375	XP_003987746.1	Felis catus
308050111	YP_003913677.1	Ferrimonas balearica DSM 9799
308050109	YP_003913675.1	Ferrimonas balearica DSM 9799

518652147	YP_008141667.1	Ferroplasma acidarmanus fer1
546145506	WP_021788041.1	Ferroplasma sp. Type II
436837795	YP_007323011.1	Fibrella aestuarina BUZ 2
496584909	WP_009284748.1	Fibrisoma limi
525000291	XP_005047884.1	Ficedula albicollis
578020649	AHI08971.1	Fimbriimonas ginsengisoli Gsoil 348
496092930	WP_008817437.1	Firmicutes
547779839	WP_022190796.1	Firmicutes bacterium CAG:240
546420484	WP_021858850.1	Firmicutes bacterium CAG:83
496141426	WP_008865933.1	Flavobacteria bacterium MS024-2A
496143301	WP_008867808.1	Flavobacteria bacterium MS024-3C
495545846	WP_008270425.1	Flavobacteriales bacterium ALC-1
365961180	YP_004942747.1	Flavobacterium columnare ATCC 49512
495086140	WP_007810963.1	Flavobacterium sp. CF136
495744549	WP_008469128.1	Flavobacterium sp. F52
495743241	WP_008467820.1	Flavobacterium sp. F52
518925659	WP_020081534.1	Flavobacterium sp. SCGC AAA160-P02
516064262	WP_017494845.1	Flavobacterium sp. WG21
516065750	WP_017496333.1	Flavobacterium sp. WG21
522016241	WP_020527512.1	Flexithrix dorotheae
550978757	WP_022726868.1	Fodinicurvata sediminis
550978816	WP_022726927.1	Fodinicurvata sediminis
527299018	EPS99821.1	Fomitopsis pinicola FP-58527 SS1
494923654	WP_007649689.1	Formosa sp. AK20
494923650	WP_007649685.1	Formosa sp. AK20
551294047	WP_022914137.1	Frankia sp. Iso899
383316637	YP_005377479.1	Frateuria aurantia DSM 6220
479198794	YP_007828020.1	Fretibacterium fastidiosum
494125569	WP_007065344.1	Fulvimarina pelagi
497268441	WP_009582658.1	Fulvivirga imtechensis
497268435	WP_009582652.1	Fulvivirga imtechensis
517319564	CCT70383.1	Fusarium fujikuroi IMI 58289
517310095	CCT61554.1	Fusarium fujikuroi IMI 58289
517315603	CCT67602.1	Fusarium fujikuroi IMI 58289
596544210	EYB24406.1	Fusarium graminearum
596550858	EYB30358.1	Fusarium graminearum
46109694	XP_381905.1	Fusarium graminearum PH-1
46127015	XP_388061.1	Fusarium graminearum PH-1
477520607	ENH72733.1	Fusarium oxysporum f. sp. cubense race 1
477512615	ENH65152.1	Fusarium oxysporum f. sp. cubense race 1
477519149	ENH71330.1	Fusarium oxysporum f. sp. cubense race 1
475667008	EMT64798.1	Fusarium oxysporum f. sp. cubense race 4
475669214	EMT66976.1	Fusarium oxysporum f. sp. cubense race 4
475676863	EMT73885.1	Fusarium oxysporum f. sp. cubense race 4
591477778	EXM08899.1	Fusarium oxysporum f. sp. cubense tropical race 4 54006

587710634	EWZ81971.1	Fusarium oxysporum f. sp. lycopersici MN25
587716092	EWZ87429.1	Fusarium oxysporum f. sp. lycopersici MN25
587721159	EWZ92496.1	Fusarium oxysporum f. sp. lycopersici MN25
590031789	EXK33647.1	Fusarium oxysporum f. sp. melonis 26406
590044621	EXK46479.1	Fusarium oxysporum f. sp. melonis 26406
590031966	EXK33824.1	Fusarium oxysporum f. sp. melonis 26406
590042915	EXK44773.1	Fusarium oxysporum f. sp. melonis 26406
587743721	EXA41437.1	Fusarium oxysporum f. sp. pisi HDV247
587743542	EXA41258.1	Fusarium oxysporum f. sp. pisi HDV247
587745254	EXA42970.1	Fusarium oxysporum f. sp. pisi HDV247
591409470	EXL44607.1	Fusarium oxysporum f. sp. radicis-lycopersici 26381
591405824	EXL40961.1	Fusarium oxysporum f. sp. radicis-lycopersici 26381
590071856	EXK99380.1	Fusarium oxysporum f. sp. raphani 54005
590070437	EXK97961.1	Fusarium oxysporum f. sp. raphani 54005
591505127	EXM34430.1	Fusarium oxysporum f. sp. vasinfectum 25433
591491454	EXM21082.1	Fusarium oxysporum f. sp. vasinfectum 25433
587702633	EWZ49238.1	Fusarium oxysporum Fo47
587693872	EWZ40477.1	Fusarium oxysporum Fo47
587700849	EWZ47454.1	Fusarium oxysporum Fo47
342880654	EGU81692.1	Fusarium oxysporum Fo5176
342884446	EGU84661.1	Fusarium oxysporum Fo5176
342885186	EGU85285.1	Fusarium oxysporum Fo5176
587665692	EWY88033.1	Fusarium oxysporum FOSC 3-a
587658278	EWY80669.1	Fusarium oxysporum FOSC 3-a
587679094	EWZ01412.1	Fusarium oxysporum FOSC 3-a
408388741	EKJ68420.1	Fusarium pseudograminearum CS3096
408389309	EKJ68770.1	Fusarium pseudograminearum CS3096
584129239	EWG38644.1	Fusarium verticillioides 7600
584141350	EWG50681.1	Fusarium verticillioides 7600
584144390	EWG53679.1	Fusarium verticillioides 7600
496279890	WP_008991928.1	Galbibacter marinus
513189796	XP_421428.4	Gallus gallus
498233252	WP_010547408.1	gamma proteobacterium HIMB30
498233249	WP_010547405.1	gamma proteobacterium HIMB30
497104266	WP_009471456.1	gamma proteobacterium HIMB55
518868573	WP_020024463.1	gamma proteobacterium SCGC AAA076-D02
518870748	WP_020026623.1	gamma proteobacterium SCGC AAA076-D13
518871157	WP_020027032.1	gamma proteobacterium SCGC AAA076-E13

518871156	WP_020027031.1	gamma proteobacterium SCGC AAA076-E13
518871866	WP_020027741.1	gamma proteobacterium SCGC AAA076-F14
516059526	WP_017490109.1	gamma proteobacterium WG36
545645245	WP_021752997.1	Gemella bergeri
489239550	WP_003147788.1	Gemella haemolysans
490771798	WP_004634022.1	Gemella morbillorum
493407964	WP_006363981.1	Gemella sanguinis
497728191	WP_010042375.1	Gemmata obscuriglobus
575461365	AHG92417.1	Gemmatimonadetes bacterium KBS708
575461360	AHG92412.1	Gemmatimonadetes bacterium KBS708
284990813	YP_003409367.1	Geodermatophilus obscurus DSM 43160
522163861	WP_020675069.1	Geopsychrobacter electrodiphilus
494893050	WP_007619095.1	Glaciecola arctica
551264342	WP_022884550.1	Glaciibacter superstes
551264371	WP_022884579.1	Glaciibacter superstes
551264373	WP_022884581.1	Glaciibacter superstes
496128168	WP_008852675.1	Gluconobacter morbifer
491325498	WP_005183461.1	Gordonia amarae
491325481	WP_005183444.1	Gordonia amarae
519014209	WP_020170084.1	Gordonia polyisoprenivorans
378716377	YP_005281266.1	Gordonia polyisoprenivorans VH2
493374214	WP_006330517.1	Gordonia rhizosphera
479173712	YP_007801695.1	Gordonibacter pamelaeae 7-10-1-b
426377053	XP_004055291.1	Gorilla gorilla gorilla
374313636	YP_005060066.1	Granulicella mallensis MP5ACTX8
322433091	YP_004210340.1	Granulicella tundricola MP5ACTX9
551261240	WP_022881474.1	Gryllotalpicola ginsengisoli
83645920	YP_434355.1	Hahella chejuensis KCTC 2396
521079502	WP_020410409.1	Hahella ganghwensis
521076068	WP_020406975.1	Hahella ganghwensis
332663198	YP_004445986.1	Haliscomenobacter hydrossis DSM 1100
495582862	WP_008307441.1	Haloarcula amylolytica
491680942	WP_005537083.1	Haloarcula argentinensis
494347286	WP_007189434.1	Haloarcula californiae
344213146	YP_004797466.1	Haloarcula hispanica ATCC 33960
490728202	WP_004590607.1	Haloarcula japonica
55379132	YP_136982.1	Haloarcula marismortui ATCC 43049
491097603	WP_004959204.1	Haloarcula sinaiiensis
490651098	WP_004516092.1	Haloarcula vallismortis
494232992	WP_007140037.1	Halobiforma lacisalsi
493725064	WP_006674436.1	Halobiforma nitratireducens
492979562	WP_006076636.1	Halococcus saccharolyticus
491180600	WP_005038962.1	Halococcus salifodinae
495847922	WP_008572501.1	Haloferax
495600043	WP_008324622.1	Haloferax elongans

491119548	WP_004977990.1	Haloferax gibbonsii
495369461	WP_008094174.1	Haloferax prahovense
495882984	WP_008607563.1	Haloferax sp. BAB2207
494486947	WP_007276420.1	Haloferax sulfurifontis
292653723	YP_003533621.1	Haloferax volcanii DS2
496829151	WP_009376761.1	Halogranum salarium
496591115	WP_009288221.1	Halomonas
512440296	WP_016416105.1	Halomonas anticariensis
512443155	WP_016418809.1	Halomonas anticariensis
512443206	WP_016418860.1	Halomonas anticariensis
512440788	WP_016416575.1	Halomonas anticariensis
512438904	WP_016414724.1	Halomonas anticariensis
494173862	WP_007113589.1	Halomonas boliviensis
494173752	WP_007113479.1	Halomonas boliviensis
307544144	YP_003896623.1	Halomonas elongata DSM 2581
307545477	YP_003897956.1	Halomonas elongata DSM 2581
307546897	YP_003899376.1	Halomonas elongata DSM 2581
517848713	WP_019018921.1	Halomonas lutea
517848711	WP_019018919.1	Halomonas lutea
515330573	WP_016855316.1	Halomonas smyrnensis
548433789	WP_022523626.1	Halomonas sp. A3H3
548420109	WP_022520341.1	Halomonas sp. A3H3
584381297	EWG98416.1	Halomonas sp. BC04
584386312	EWH03082.1	Halomonas sp. BC04
584386634	EWH03384.1	Halomonas sp. BC04
546177901	WP_021818656.1	Halomonas sp. BJGMM-B45
546180177	WP_021820908.1	Halomonas sp. BJGMM-B45
496391437	WP_009100427.1	Halomonas sp. GFAJ-1
496244391	WP_008957776.1	Halomonas sp. HAL1
496243984	WP_008957369.1	Halomonas sp. HAL1
498315795	WP_010629951.1	Halomonas sp. KM-1
498312555	WP_010626711.1	Halomonas sp. KM-1
552545866	WP_023004224.1	Halomonas sp. PBN3
497407390	WP_009721588.1	Halomonas sp. TD01
515483193	WP_016916461.1	Halomonas stevensii
496588255	WP_009286883.1	Halomonas titanicae
517746371	WP_018916579.1	Halomonas zhanjiangensis
495664170	WP_008388749.1	Halosarcina pallida
573481958	AHG00369.1	Halostagnicola larsenii XH-48
496171357	WP_008895864.1	Haloterrigena salina
554807179	XP_005914513.1	Haplochromis burtoni
555704675	ESO07908.1	Helobdella robusta
493510522	WP_006464873.1	Herbaspirillum frisingense
515269373	WP_016832043.1	Herbaspirillum lusitanum
516022478	WP_017453061.1	Herbaspirillum seropedicae
300309517	YP_003773609.1	Herbaspirillum seropedicae SmR1
495150327	WP_007875133.1	Herbaspirillum sp. CF444
495609653	WP_008334232.1	Herbaspirillum sp. GW103
495388378	WP_008113081.1	Herbaspirillum sp. YR522
159899018	YP_001545265.1	Herpetosiphon aurantiacus DSM 785
512855306	XP_004888295.1	Heterocephalus glaber
512950155	XP_004837517.1	Heterocephalus glaber
494372180	WP_007198955.1	Hoeflea phototrophica
494373738	WP_007199816.1	Hoeflea phototrophica
517241432	WP_018430250.1	Hoeflea sp. 108
517241430	WP_018430248.1	Hoeflea sp. 108
15082426	AAH12131.1	Homo sapiens
21389361	NP_653182.1	Homo sapiens
578825445	XP_006720079.1	Homo sapiens
551279435	WP_022899549.1	Humibacter albus
221105094	XP_002160119.1	Hydra vulgaris
495512835	WP_008237480.1	Imtechella halotolerans
496326402	WP_009035580.1	Indibacter alkaliphilus
317126279	YP_004100391.1	Intrasporangium calvum DSM 43043
317126273	YP_004100385.1	Intrasporangium calvum DSM 43043
586971661	EWT07023.1	Intrasporangium chromatireducens Q5-1
586971667	EWT07029.1	Intrasporangium chromatireducens Q5-1
507530406	XP_004649287.1	Jaculus jaculus
89054020	YP_509471.1	Jannaschia sp. CCS1
516490051	WP_017878495.1	Janthinobacterium sp. CG3
495727362	WP_008451941.1	Janthinobacterium sp. HH01
491684031	WP_005540169.1	Johnsonella ignava
495886875	WP_008611454.1	Joostella marina
516951438	WP_018181128.1	Kaistia granuli
310830249	YP_003965349.1	Ketogulonicigenium vulgare Y25
522083138	WP_020594347.1	Kiloniella laminariae
522081177	WP_020592386.1	Kiloniella laminariae
357393553	YP_004908394.1	Kitasatospora setae KM-6054
357393555	YP_004908396.1	Kitasatospora setae KM-6054
507089527	WP_016160240.1	Klebsiella
556470290	WP_023322031.1	Klebsiella pneumoniae
556335582	WP_023297019.1	Klebsiella pneumoniae
490309392	WP_004204193.1	Klebsiella pneumoniae
556267326	WP_023288208.1	Klebsiella pneumoniae
556467911	WP_023319670.1	Klebsiella pneumoniae
206576988	YP_002240611.1	Klebsiella pneumoniae 342
583546968	EWE10358.1	Klebsiella pneumoniae BIDMC 46b
550447455	YP_008663303.1	Klebsiella pneumoniae CG43
583704215	EWF66363.1	Klebsiella pneumoniae MGH 35
583698999	EWF61185.1	Klebsiella pneumoniae MGH 43
583478151	EWD42050.1	Klebsiella pneumoniae UCI 26
496083060	WP_008807567.1	Klebsiella sp. 1_1_55
591283029	CDN04099.1	Klebsiella sp. 18A069
288937307	YP_003441366.1	Klebsiella variicola At-22
489715758	WP_003619882.1	Komagataeibacter hansenii
494154142	WP_007093885.1	Kordia algicida
521053509	WP_020385460.1	Kribbella catacumbae
521057645	WP_020389596.1	Kribbella catacumbae
284034417	YP_003384348.1	Kribbella flavida DSM 17836

332291525	YP_004430134.1	Krokinobacter sp. 4H-3-7-5
495181795	WP_007906585.1	Ktedonobacter racemifer
495182323	WP_007907113.1	Ktedonobacter racemifer
495179900	WP_007904690.1	Ktedonobacter racemifer
518793270	WP_019950559.1	Kushneria aurantia
578005346	AHH93604.1	Kutzneria albida DSM 43870
585092721	EWM14662.1	Kutzneria sp. 744
552541681	WP_023000145.1	Labrenzia
552541677	WP_023000141.1	Labrenzia
493994423	WP_006937102.1	Labrenzia aggregata
493994427	WP_006937106.1	Labrenzia aggregata
495468829	WP_008193519.1	Labrenzia alexandrii
495463964	WP_008188656.1	Labrenzia alexandrii
496557251	WP_009263326.1	Lachnospiraceae
496263625	WP_008977010.1	Lachnospiraceae
497077691	WP_009461342.1	Lachnospiraceae bacterium 2_1_46FAA
510890479	WP_016223794.1	Lachnospiraceae bacterium 3-2
510889445	WP_016222783.1	Lachnospiraceae bacterium 3-2
496542038	WP_009248186.1	Lachnospiraceae bacterium 5_1_57FAA
494200202	WP_007124906.1	Lactobacillus antri
489810161	WP_003714017.1	Lactobacillus oris
489811188	WP_003715042.1	Lactobacillus oris
489798985	WP_003702876.1	Lactobacillus salivarius
574961650	CDK35962.1	Lactobacillus salivarius cp400
498310803	WP_010624959.1	Lactobacillus versmoldensis
556946625	XP_005986491.1	Latimeria chalumnae
312130496	YP_003997836.1	Leadbetterella byssophila DSM 17132
516893905	WP_018152247.1	Leeia oryzae
516892601	WP_018151492.1	Leeia oryzae
497464642	WP_009778840.1	Leeuwenhoekiella blandensis
568284937	YP_008973076.1	Leisingera methylohalidivorans DSM 14336
568284935	YP_008973074.1	Leisingera methylohalidivorans DSM 14336
573888883	XP_006632209.1	Lepisosteus oculatus
585155525	XP_006730098.1	Leptonychotes weddellii
602711731	XP_007466101.1	Lipotes vexillifer
517161861	WP_018350679.1	Longispora albida
517163469	WP_018352287.1	Longispora albida
556105508	ESO94160.1	Lottia gigantea
556105489	ESO94141.1	Lottia gigantea
344273853	XP_003408733.1	Loxodonta africana
490337899	WP_004224302.1	Lysinibacillus fusiformis
169825719	YP_001695877.1	Lysinibacillus sphaericus C3-41
601088271	EYR66081.1	Lysobacter capsici AZ78
544447586	XP_005561443.1	Macaca fascicularis
355693320	EHH27923.1	Macaca mulatta
109083793	XP_001094065.1	Macaca mulatta
407918904	EKG12165.1	Macrophomina phaseolina MS6
305666708	YP_003862995.1	Maribacter sp. HTCC2170
305665980	YP_003862267.1	Maribacter sp. HTCC2170
114568849	YP_755529.1	Maricaulis maris MCS10

497459635	WP_009773833.1	marine actinobacterium PHSC20C1
516700916	WP_018052109.1	Marinimicrobia bacterium SCGC AB-629-J13
495899420	WP_008623999.1	Mariniradius saccharolyticus
385330663	YP_005884614.1	Marinobacter adhaerens HP15
494259899	WP_007151900.1	Marinobacter algicola
120555056	YP_959407.1	Marinobacter aquaeolei VT8
387813506	YP_005428988.1	Marinobacter hydrocarbonoclasticus ATCC 49840
501088391	WP_012138648.1	Marinobacter lipolyticus
495451233	WP_008175927.1	Marinobacter manganoxydans
490720553	WP_004583115.1	Marinobacter nanhaiticus
496223508	WP_008937543.1	Marinobacter santoriniensis
582025531	AHI28867.1	Marinobacter sp. A3d10
399546107	YP_006559415.1	Marinobacter sp. BSs20148
552528590	WP_022993488.1	Marinobacter sp. C1S70
494591022	WP_007349615.1	Marinobacter sp. ELB17
552559757	WP_023012155.1	Marinobacter sp. EN3
552204363	WP_022989454.1	Marinobacter sp. ES-1
552554043	WP_023009383.1	Marinobacter sp. EVN1
498009412	WP_010323568.1	Marinobacterium stanieri
326793361	YP_004311181.1	Marinomonas mediterranea MMB-1
333906444	YP_004480030.1	Marinomonas posidonica IVIA-Po-181
567432457	WP_024024331.1	Marinomonas sp. D104
497518434	WP_009832632.1	Marinomonas sp. MED121
152994094	YP_001338929.1	Marinomonas sp. MWYL1
516721842	WP_018064680.1	Martelella mediterranea
498939595	XP_004541895.1	Maylandia zebra
498939591	XP_004541894.1	Maylandia zebra
517463199	WP_018633932.1	Meganema perideroedes
520879191	WP_020310842.1	Megasphaera
348025670	YP_004765474.1	Megasphaera elsdenii DSM 20460
494000017	WP_006942606.1	Megasphaera micronuciformis
332685776	YP_004455550.1	Melissococcus plutonius ATCC 35311
379726838	YP_005319023.1	Melissococcus plutonius DAT561
527263017	XP_005149740.1	Melopsittacus undulatus
524943394	XP_005072835.1	Mesocricetus auratus
498202842	WP_010516998.1	Mesoflavibacter zeaxanthinifaciens
563472306	WP_023697251.1	Mesorhizobium
563546721	WP_023752640.1	Mesorhizobium
563549669	WP_023755542.1	Mesorhizobium
563468131	WP_023693148.1	Mesorhizobium
563493281	WP_023712006.1	Mesorhizobium
563459019	WP_023684136.1	Mesorhizobium
563481289	WP_023704920.1	Mesorhizobium
563531699	WP_023737892.1	Mesorhizobium
563560515	WP_023766090.1	Mesorhizobium
563460014	WP_023685122.1	Mesorhizobium
563479906	WP_023703542.1	Mesorhizobium

563540585	WP_023746646.1	Mesorhizobium
563537057	WP_023743199.1	Mesorhizobium
563549798	WP_023755668.1	Mesorhizobium
563467631	WP_023692651.1	Mesorhizobium
563493903	WP_023712622.1	Mesorhizobium
496110246	WP_008834753.1	Mesorhizobium alhagi
496109807	WP_008834314.1	Mesorhizobium alhagi
493226774	WP_006204845.1	Mesorhizobium amorphae
493229838	WP_006206599.1	Mesorhizobium amorphae
493229785	WP_006206582.1	Mesorhizobium amorphae
493217297	WP_006199511.1	Mesorhizobium amorphae
433772654	YP_007303121.1	Mesorhizobium australicum WSM2073
433771588	YP_007302055.1	Mesorhizobium australicum WSM2073
319780970	YP_004140446.1	Mesorhizobium ciceri biovar biserrulae WSM1271
319779924	YP_004139400.1	Mesorhizobium ciceri biovar biserrulae WSM1271
319777967	YP_004134397.1	Mesorhizobium ciceri biovar biserrulae WSM1271
518700443	WP_019861794.1	Mesorhizobium loti
518698585	WP_019860078.1	Mesorhizobium loti
13473394	NP_104961.1	Mesorhizobium loti MAFF303099
13475682	NP_107249.1	Mesorhizobium loti MAFF303099
563516076	ETA71432.1	Mesorhizobium loti R7A
496149215	WP_008873722.1	Mesorhizobium metallidurans
496147906	WP_008872413.1	Mesorhizobium metallidurans
496151591	WP_008876098.1	Mesorhizobium metallidurans
496153980	WP_008878487.1	Mesorhizobium metallidurans
337265784	YP_004609839.1	Mesorhizobium opportunistum WSM2075
337267309	YP_004611364.1	Mesorhizobium opportunistum WSM2075
337266589	YP_004610644.1	Mesorhizobium opportunistum WSM2075
564008206	WP_023834357.1	Mesorhizobium sp. L103C105A0
564006398	WP_023832612.1	Mesorhizobium sp. L103C119B0
564003028	WP_023829296.1	Mesorhizobium sp. L103C120A0
564002293	WP_023828568.1	Mesorhizobium sp. L103C120A0
564000701	WP_023827032.1	Mesorhizobium sp. L103C131B0
563996039	WP_023822440.1	Mesorhizobium sp. L2C054A000
563989234	WP_023818228.1	Mesorhizobium sp. L2C066B000
563993067	WP_023819533.1	Mesorhizobium sp. L2C066B000
563983400	WP_023816971.1	Mesorhizobium sp. L2C067A000
563939675	WP_023811801.1	Mesorhizobium sp. L2C084A000
563938637	WP_023811648.1	Mesorhizobium sp. L2C084A000
563925257	WP_023809519.1	Mesorhizobium sp. L2C084A000
563892309	WP_023806582.1	Mesorhizobium sp. L2C089B000
563830210	WP_023800039.1	Mesorhizobium sp. L48C026A00
563857230	WP_023803746.1	Mesorhizobium sp. L48C026A00
563791880	WP_023794841.1	Mesorhizobium sp. LNHC209A00
563781309	WP_023792706.1	Mesorhizobium sp. LNHC209A00
563579101	WP_023784249.1	Mesorhizobium sp. LNHC220B00
563578179	WP_023783332.1	Mesorhizobium sp. LNHC220B00
563573205	WP_023778423.1	Mesorhizobium sp. LNHC221B00
563566225	WP_023771723.1	Mesorhizobium sp. LNHC229A00
563567037	WP_023772534.1	Mesorhizobium sp. LNHC229A00
563560114	WP_023765690.1	Mesorhizobium sp. LNHC232B00
563552827	WP_023758598.1	Mesorhizobium sp. LNHC252B00
563552919	WP_023758689.1	Mesorhizobium sp. LNHC252B00
563552486	WP_023758258.1	Mesorhizobium sp. LNHC252B00
563541245	WP_023747303.1	Mesorhizobium sp. LNJC391B00
563538864	WP_023744963.1	Mesorhizobium sp. LNJC394B00
563540046	WP_023746136.1	Mesorhizobium sp. LNJC394B00
563536075	WP_023742231.1	Mesorhizobium sp. LNJC395A00
563530634	WP_023736863.1	Mesorhizobium sp. LNJC399B00
563526711	WP_023732988.1	Mesorhizobium sp. LNJC403B00
563526227	WP_023732505.1	Mesorhizobium sp. LNJC403B00
563522309	WP_023728653.1	Mesorhizobium sp. LNJC405B00
563519435	WP_023725827.1	Mesorhizobium sp. LSHC412B00
563501759	WP_023719661.1	Mesorhizobium sp. LSHC420B00
563500014	WP_023717924.1	Mesorhizobium sp. LSHC420B00
563475392	WP_023700251.1	Mesorhizobium sp. LSJC264A00
563478741	WP_023702636.1	Mesorhizobium sp. LSJC264A00
563474694	WP_023699558.1	Mesorhizobium sp. LSJC264A00
563473487	WP_023698416.1	Mesorhizobium sp. LSJC265A00
563474043	WP_023698957.1	Mesorhizobium sp. LSJC265A00
563463859	WP_023688921.1	Mesorhizobium sp. LSJC269B00
563464983	WP_023690037.1	Mesorhizobium sp. LSJC269B00
563452679	WP_023678053.1	Mesorhizobium sp. LSJC280B00

563455655	WP_023680949.1	Mesorhizobium sp. LSJC280B00
563449907	WP_023676374.1	Mesorhizobium sp. LSJC280B00
563441848	WP_023668714.1	Mesorhizobium sp. LSJC285A00
563446050	WP_023672875.1	Mesorhizobium sp. LSJC285A00
493371271	WP_006327593.1	Mesorhizobium sp. STM 4661
493368816	WP_006325154.1	Mesorhizobium sp. STM 4661
493373514	WP_006329825.1	Mesorhizobium sp. STM 4661
517269468	WP_018458286.1	Mesorhizobium sp. WSM4349
322694231	EFY86066.1	Metarhizium acridum CQMa 102
322711542	EFZ03115.1	Metarhizium anisopliae ARSEF 23
322706529	EFY98109.1	Metarhizium anisopliae ARSEF 23
220923857	YP_002499159.1	Methylobacterium nodulans ORS 2060
220923865	YP_002499167.1	Methylobacterium nodulans ORS 2060
170741627	YP_001770282.1	Methylobacterium sp. 4-46
170741990	YP_001770645.1	Methylobacterium sp. 4-46
494838461	WP_007564561.1	Methylobacterium sp. GXF4
517071436	WP_018260254.1	Methylobacterium sp. WSM2598
517075275	WP_018264093.1	Methylobacterium sp. WSM2598
565872386	WP_023953736.1	Microbacterium sp. TS-1
518011541	WP_019181749.1	Microbacterium yannicii
488944428	WP_002855503.1	Micrococcus luteus
516058028	WP_017488611.1	Micrococcus luteus
336118089	YP_004572857.1	Microlunatus phosphovorus NM-1
336118060	YP_004572828.1	Microlunatus phosphovorus NM-1
336118063	YP_004572831.1	Microlunatus phosphovorus NM-1
336115874	YP_004570640.1	Microlunatus phosphovorus NM-1
315505122	YP_004084009.1	Micromonospora sp. L5
585245434	EWM64966.1	Micromonospora sp. M42
488792410	WP_002704816.1	Microscilla marina
488783161	WP_002695568.1	Microscilla marina
531997348	XP_005343233.1	Microtus ochrogaster
497163564	WP_009492975.1	Microvirga lotononidis
557623002	YP_008784134.1	missing catalytic residues
493976736	WP_006919769.1	missing catalytic residues
491636508	WP_005494037.1	missing catalytic residues
497329749	WP_009643962.1	Mogibacterium sp. CM50
497329448	WP_009643661.1	Mogibacterium sp. CM50
576980661	EUC53405.1	Mogibacterium timidum ATCC 33093
554906233	ESK88189.1	Moniliophthora roreri MCA 2997
554908909	ESK90533.1	Moniliophthora roreri MCA 2997
126282889	XP_001377162.1	Monodelphis domestica
495787942	WP_008512521.1	Mucilaginibacter paludis

344201938	YP_004787081.1	Muricauda ruestringensis DSM 13258
21313074	NP_080314.1	Mus musculus
13435795	AAH04753.1	Mus musculus
148704596	EDL36543.1	Mus musculus
511828384	XP_004738925.1	Mustela putorius furo
367030697	XP_003664632.1	Myceliophthora thermophila ATCC 42464
597305095	CDO06720.1	Mycobacterium cosmeticum
518814339	WP_019970293.1	Mycobacterium sp. 141
518814362	WP_019970316.1	Mycobacterium sp. 141
517431494	WP_018602439.1	Mycobacterium sp. 155
517431467	WP_018602413.1	Mycobacterium sp. 155
558173685	XP_006099650.1	Myotis lucifugus
442317802	YP_007357823.1	Myxococcus stipitatus DSM 14675
108758190	YP_634330.1	Myxococcus xanthus DK 1622
493717294	WP_006666828.1	Natrialba aegyptia
493042320	WP_006106937.1	Natrialba asiatica
493877956	WP_006824303.1	Natrialba taiwanensis
493478044	WP_006432982.1	Natrinema versiforme
491715365	WP_005559587.1	Natronococcus amylolyticus
302882811	XP_003040311.1	Nectria haematococca mpVI 77-13-4
302886928	XP_003042353.1	Nectria haematococca mpVI 77-13-4
302919683	XP_003052914.1	Nectria haematococca mpVI 77-13-4
302889982	XP_003043876.1	Nectria haematococca mpVI 77-13-4
302891297	XP_003044531.1	Nectria haematococca mpVI 77-13-4
302886826	XP_003042302.1	Nectria haematococca mpVI 77-13-4
302907276	XP_003049610.1	Nectria haematococca mpVI 77-13-4
302888363	XP_003043068.1	Nectria haematococca mpVI 77-13-4
302882051	XP_003039936.1	Nectria haematococca mpVI 77-13-4
489852739	WP_003756414.1	Neisseria sicca
515029511	WP_016686765.1	Neisseria sicca
496406917	WP_009115781.1	Neisseria wadsworthii
156368785	XP_001627872.1	Nematostella vectensis
156352977	XP_001622857.1	Nematostella vectensis
156317921	XP_001618064.1	Nematostella vectensis
485922238	EOD47793.1	Neofusicoccum parvum UCRNP2
485929380	EOD52796.1	Neofusicoccum parvum UCRNP2
584004362	XP_006797184.1	Neolamprologus brichardi
584004360	XP_006797183.1	Neolamprologus brichardi
119499151	XP_001266333.1	Neosartorya fischeri NRRL 181
551253652	WP_022873897.1	Nesterenkonia alba
375145878	YP_005008319.1	Niastella koreensis GR20-10
494068819	WP_007010886.1	Nitratireductor aquibiodomus
497054508	WP_009449384.1	Nitratireductor indicus
497054502	WP_009449382.1	Nitratireductor indicus
495871608	WP_008596187.1	Nitratireductor pacificus

495870406	WP_008594985.1	Nitratireductor pacificus
496344882	WP_009054060.1	Nitritalea halalkaliphila
407644053	YP_006807812.1	Nocardia brasiliensis ATCC 700358
516219984	WP_017623947.1	Nocardiopsis chromatogenes
516216555	WP_017620518.1	Nocardiopsis gilva
516172914	WP_017593035.1	Nocardiopsis potens
441595505	XP_003267893.2	Nomascus leucogenys
522033582	WP_020544791.1	Nonomuraea coxensis
522033583	WP_020544792.1	Nonomuraea coxensis
518476182	WP_019646389.1	Novispirillum itersonii
518476180	WP_019646387.1	Novispirillum itersonii
544817850	WP_021234184.1	Novosphingobium lindaniclasticum
495340851	WP_008065585.1	Novosphingobium nitrogenifigens
550929918	WP_022678380.1	Novosphingobium sp. B-7
496229331	WP_008943366.1	Oceanibaculum indicum
496229333	WP_008943368.1	Oceanibaculum indicum
494189467	WP_007120758.1	Oceanibulbus indolifex
494189463	WP_007120756.1	Oceanibulbus indolifex
494465405	WP_007254929.1	Oceanicola granulosus
494465404	WP_007254928.1	Oceanicola granulosus
374334335	YP_005091022.1	Oceanimonas sp. GK1
504176989	XP_004597715.1	Ochotona princeps
498347303	WP_010661459.1	Ochrobactrum anthropi
498344106	WP_010658262.1	Ochrobactrum anthropi
590080915	EXL07224.1	Ochrobactrum anthropi
153008445	YP_001369660.1	Ochrobactrum anthropi ATCC 49188
153007784	YP_001368999.1	Ochrobactrum anthropi ATCC 49188
493513502	WP_006467803.1	Ochrobactrum intermedium
549933018	WP_022568915.1	Ochrobactrum intermedium
493516798	WP_006471041.1	Ochrobactrum intermedium
493511771	WP_006466095.1	Ochrobactrum intermedium
495151999	WP_007876805.1	Ochrobactrum sp. CDB2
495148742	WP_007873549.1	Ochrobactrum sp. CDB2
545308835	WP_021588058.1	Ochrobactrum sp. EGD-AQ16
545306953	WP_021586216.1	Ochrobactrum sp. EGD-AQ16
478181358	YP_007702428.1	Octadecabacter antarcticus 307
478177906	YP_007698979.1	Octadecabacter arcticus 238
507619803	XP_004624997.1	Octodon degus
472392975	XP_004415760.1	Odobenus rosmarus divergens
562974169	ESW99897.1	Ogataea parapolymorpha DL-1
565322866	ETE73509.1	Ophiophagus hannah
466055919	XP_004279343.1	Orcinus orca
542232935	XP_005454800.1	Oreochromis niloticus
348524917	XP_003449969.1	Oreochromis niloticus
497220727	WP_009534989.1	Oribacterium
494273400	WP_007158106.1	Oribacterium sinus
497223232	WP_009537494.1	Oribacterium sp. ACB7
545668688	WP_021775011.1	Oribacterium sp. oral taxon 078
496505691	WP_009213974.1	Oribacterium sp. oral taxon 078
496992334	WP_009429838.1	Oribacterium sp. oral taxon 108
551302456	WP_022922413.1	Ornithinimicrobium pekingense
291413628	XP_002723072.1	Oryctolagus cuniculus
432946180	XP_004083807.1	Oryzias latipes
518645209	WP_019815228.1	Osedax symbiont Rs1
518645096	WP_019815118.1	Osedax symbiont Rs1
520768530	WP_020286306.1	Osedax symbiont Rs2
520772717	WP_020287583.1	Osedax symbiont Rs2
395843389	XP_003794468.1	Otolemur garnettii
426233466	XP_004010738.1	Ovis aries
522193766	WP_020701233.1	Oxalobacteraceae bacterium AB_14
498033334	WP_010347490.1	Paenibacillus peoriae
498032362	WP_010346518.1	Paenibacillus peoriae
515239020	WP_016821425.1	Paenibacillus polymyxa
565990887	YP_008910479.1	Paenibacillus polymyxa CR1
595632423	AHM64681.1	Paenibacillus polymyxa SQR-21
517582363	WP_018752571.1	Paenibacillus sanguinis
517582364	WP_018752572.1	Paenibacillus sanguinis
494673993	WP_007431933.1	Paenibacillus sp. Aloe-11
515995749	WP_017426332.1	Paenibacillus sp. ICGEB2008
374322435	YP_005075564.1	Paenibacillus terrae HPL-003
397523351	XP_003831698.1	Pan paniscus
556759230	XP_005974943.1	Pantholops hodgsonii
556775862	XP_005983043.1	Pantholops hodgsonii
556731356	XP_005961363.1	Pantholops hodgsonii
556766714	XP_005985887.1	Pantholops hodgsonii
556748029	XP_005969462.1	Pantholops hodgsonii
556756027	XP_005973366.1	Pantholops hodgsonii
556759228	XP_005974942.1	Pantholops hodgsonii
515918328	WP_017348911.1	Pantoea sp. A4
515918480	WP_017349063.1	Pantoea sp. A4
515918338	WP_017348921.1	Pantoea sp. A4
544759644	WP_021186478.1	Pantoea sp. AS-PWVM4
317053035	YP_004119389.1	Pantoea sp. At-9b
402876332	XP_003901927.1	Papio anubis
530320680	YP_008406641.1	Paracoccus aminophilus JCM 7686
530320682	YP_008406643.1	Paracoccus aminophilus JCM 7686
119387581	YP_918615.1	Paracoccus denitrificans PD1222
119383796	YP_914852.1	Paracoccus denitrificans PD1222
119386179	YP_917234.1	Paracoccus denitrificans PD1222
119383929	YP_914985.1	Paracoccus denitrificans PD1222
498082960	WP_010397116.1	Paracoccus sp. TRP
498082962	WP_010397118.1	Paracoccus sp. TRP
576322395	ETZ22079.1	Pedobacter sp. V48
357383252	YP_004897976.1	Pelagibacterium halotolerans B2
357384257	YP_004898981.1	Pelagibacterium halotolerans B2
255930749	XP_002556931.1	Penicillium chrysogenum Wisconsin 54-1255

255935273	XP_002558663.1	Penicillium chrysogenum Wisconsin 54-1255
525581534	EPS27784.1	Penicillium oxalicum 114-2
584406114	CDM38205.1	Penicillium roqueforti
544988145	WP_021386243.1	Peptoclostridium difficile
545031873	WP_021412177.1	Peptoclostridium difficile
544970884	WP_021373750.1	Peptoclostridium difficile
545034084	WP_021413071.1	Peptoclostridium difficile
126700857	YP_001089754.1	Peptoclostridium difficile 630
488932400	WP_002843475.1	Peptostreptococcus
589956348	XP_006990785.1	Peromyscus maniculatus bairdii
573054782	ETS74717.1	Pestalotiopsis fici W106-1
573067515	ETS87043.1	Pestalotiopsis fici W106-1
573066728	ETS86256.1	Pestalotiopsis fici W106-1
573059576	ETS79374.1	Pestalotiopsis fici W106-1
573054166	ETS74125.1	Pestalotiopsis fici W106-1
518128286	WP_019298494.1	Phaeobacter gallaeciensis
518128283	WP_019298491.1	Phaeobacter gallaeciensis
400760014	YP_006589617.1	Phaeobacter gallaeciensis 2.10
400753363	YP_006561731.1	Phaeobacter gallaeciensis 2.10
568305411	YP_008977695.1	Phaeobacter gallaeciensis DSM 26640
568304675	YP_008976960.1	Phaeobacter gallaeciensis DSM 26640
399994797	YP_006575029.1	Phaeobacter inhibens DSM 17395
399991723	YP_006571963.1	Phaeobacter inhibens DSM 17395
169598914	XP_001792880.1	Phaeosphaeria nodorum SN15
253989628	YP_003040984.1	Photorhabdus asymbiotica
604177701	EYU14422.1	Photorhabdus luminescens BA1
37526156	NP_929500.1	Photorhabdus luminescens subsp. laumondii TTO1
544915404	WP_021325140.1	Photorhabdus temperata
572732832	ETS31018.1	Photorhabdus temperata subsp. khanii NC19
495397180	WP_008121880.1	Phyllobacterium sp. YR531
495403038	WP_008127737.1	Phyllobacterium sp. YR531
593753998	XP_007115895.1	Physeter catodon
593753996	XP_007115894.1	Physeter catodon
283779040	YP_003369795.1	Pirellula staleyi DSM 6068
325109793	YP_004270861.1	Planctomyces brasiliensis DSM 5305
296122957	YP_003630735.1	Planctomyces limnophilus DSM 3776
571138016	ETP67359.1	Planomicrobium glaciei CHR43
494031068	WP_006973205.1	Plesiocystis pacifica
494031073	WP_006973210.1	Plesiocystis pacifica
495138853	WP_007863660.1	Polaromonas sp. CF318
197101771	NP_001125373.1	Pongo abelii
517289662	WP_018478480.1	Pontibacter roseus
494933487	WP_007659520.1	Pontibacter sp. BAB1700
518862376	WP_020018266.1	Promicromonospora sukumoe
518859059	WP_020014949.1	Promicromonospora sukumoe
410866962	YP_006981573.1	Propionibacterium acidipropionici ATCC 4875

493710333	WP_006659977.1	Providencia alcalifaciens
493712240	WP_006661854.1	Providencia alcalifaciens
577074818	EUD11000.1	Providencia alcalifaciens 205/92
573504964	ETT05246.1	Providencia alcalifaciens F90-2004
577057148	EUC94566.1	Providencia alcalifaciens PAL-2
573498639	ETS99164.1	Providencia alcalifaciens PAL-3
491048988	WP_004910640.1	Providencia rettgeri
490375514	WP_004255115.1	Providencia rettgeri
493866528	WP_006813168.1	Providencia rustigianii
491057770	WP_004919407.1	Providencia stuartii
386745323	YP_006218502.1	Providencia stuartii MRSN 2154
518002176	WP_019172384.1	Pseudaminobacter salicylatoxidans
518001859	WP_019172067.1	Pseudaminobacter salicylatoxidans
517999945	WP_019170153.1	Pseudaminobacter salicylatoxidans
498049713	WP_010363869.1	Pseudoalteromonas citrea
498290502	WP_010604658.1	Pseudoalteromonas flavipulchra
498290523	WP_010604679.1	Pseudoalteromonas flavipulchra
515077247	WP_016707040.1	Pseudoalteromonas haloplanktis
515077232	WP_016707025.1	Pseudoalteromonas haloplanktis
584418748	EWH04345.1	Pseudoalteromonas lipolytica SCSIO 04301
491636498	WP_005494027.1	Pseudoalteromonas luteoviolacea
557381667	WP_023402095.1	Pseudoalteromonas luteoviolacea
498243688	WP_010557844.1	Pseudoalteromonas marina
498243684	WP_010557840.1	Pseudoalteromonas marina
498057282	WP_010371438.1	Pseudoalteromonas piscicida
498058404	WP_010372560.1	Pseudoalteromonas piscicida
498071369	WP_010385525.1	Pseudoalteromonas rubra
498067953	WP_010382109.1	Pseudoalteromonas rubra
515784840	WP_017217281.1	Pseudoalteromonas sp. NJ631
515785039	WP_017217480.1	Pseudoalteromonas sp. NJ631
586922275	EWS99345.1	Pseudoalteromonas sp. SCSIO_11900
586922271	EWS99341.1	Pseudoalteromonas sp. SCSIO_11900
498247509	WP_010561665.1	Pseudoalteromonas spongiae
498247385	WP_010561541.1	Pseudoalteromonas spongiae
497524302	WP_009838500.1	Pseudoalteromonas tunicata
452985388	EME85145.1	Pseudocercospora fijiensis CIRAD86
550961593	WP_022709904.1	Pseudochrobactrum sp. AO18b
518449570	WP_019619777.1	Pseudoclavibacter faecalis
496239583	WP_008952968.1	Pseudogulbenkiania ferrooxidans
496239605	WP_008952990.1	Pseudogulbenkiania ferrooxidans
496239610	WP_008952995.1	Pseudogulbenkiania ferrooxidans

545112876	WP_021475786.1	Pseudogulbenkiania ferrooxidans
347540320	YP_004847745.1	Pseudogulbenkiania sp. NH8B
347539259	YP_004846684.1	Pseudogulbenkiania sp. NH8B
347539264	YP_004846689.1	Pseudogulbenkiania sp. NH8B
495191264	WP_007916054.1	Pseudomonas
517758087	WP_018928295.1	Pseudomonas
497896123	WP_010210279.1	Pseudomonas
517758097	WP_018928305.1	Pseudomonas
553796692	WP_023127317.1	Pseudomonas aeruginosa
489252434	WP_003160463.1	Pseudomonas aeruginosa
187939704	ACD38846.1	Pseudomonas aeruginosa
553752523	WP_023085326.1	Pseudomonas aeruginosa
553765165	WP_023097486.1	Pseudomonas aeruginosa
553774594	WP_023106432.1	Pseudomonas aeruginosa
553752526	WP_023085329.1	Pseudomonas aeruginosa
553765163	WP_023097484.1	Pseudomonas aeruginosa
553756265	WP_023088954.1	Pseudomonas aeruginosa
518201790	WP_019371998.1	Pseudomonas aeruginosa
553765546	WP_023097860.1	Pseudomonas aeruginosa
489225108	WP_003133510.1	Pseudomonas aeruginosa
489211730	WP_003120521.1	Pseudomonas aeruginosa
515716254	WP_017148854.1	Pseudomonas aeruginosa
553780562	WP_023112188.1	Pseudomonas aeruginosa
553767582	WP_023099819.1	Pseudomonas aeruginosa
489254292	WP_003162279.1	Pseudomonas aeruginosa
489237084	WP_003145351.1	Pseudomonas aeruginosa
550048135	WP_022580868.1	Pseudomonas aeruginosa
510946054	WP_016263761.1	Pseudomonas aeruginosa
553780567	WP_023112193.1	Pseudomonas aeruginosa
489195738	WP_003105050.1	Pseudomonas aeruginosa
490478401	WP_004348758.1	Pseudomonas aeruginosa
557682525	WP_023436523.1	Pseudomonas aeruginosa
565828678	WP_023912528.1	Pseudomonas aeruginosa
489201659	WP_003110832.1	Pseudomonas aeruginosa
553765549	WP_023097863.1	Pseudomonas aeruginosa
553775103	WP_023106936.1	Pseudomonas aeruginosa
553777263	WP_023109048.1	Pseudomonas aeruginosa
489214407	WP_003123108.1	Pseudomonas aeruginosa
489211740	WP_003120530.1	Pseudomonas aeruginosa
553795702	WP_023126360.1	Pseudomonas aeruginosa
611851105	EZO27480.1	Pseudomonas aeruginosa 3575
478479954	YP_007710605.1	Pseudomonas aeruginosa B136-33
478479968	YP_007710619.1	Pseudomonas aeruginosa B136-33
611946975	EZP22915.1	Pseudomonas aeruginosa BWH049
611929777	EZP05781.1	Pseudomonas aeruginosa BWH054
611917580	EZO93637.1	Pseudomonas aeruginosa BWH054
574085650	ETV62105.1	Pseudomonas aeruginosa BWHPSA037
574077502	ETV54001.1	Pseudomonas aeruginosa BWHPSA038
574049483	ETV26111.1	Pseudomonas aeruginosa BWHPSA042
574029053	ETV05776.1	Pseudomonas aeruginosa BWHPSA046
574029067	ETV05790.1	Pseudomonas aeruginosa BWHPSA046
392985263	YP_006483850.1	Pseudomonas aeruginosa DK2
218892758	YP_002441627.1	Pseudomonas aeruginosa LESB58
218892771	YP_002441640.1	Pseudomonas aeruginosa LESB58
386059822	YP_005976344.1	Pseudomonas aeruginosa M18
386059837	YP_005976359.1	Pseudomonas aeruginosa M18
571152673	CDH72181.1	Pseudomonas aeruginosa MH38
571152658	CDH72193.1	Pseudomonas aeruginosa MH38
564953776	YP_008886346.1	Pseudomonas aeruginosa MTB-1
386066631	YP_005981935.1	Pseudomonas aeruginosa NCGM2.S1
386066634	YP_005981938.1	Pseudomonas aeruginosa NCGM2.S1
386065091	YP_005980395.1	Pseudomonas aeruginosa NCGM2.S1
386065077	YP_005980381.1	Pseudomonas aeruginosa NCGM2.S1
575872237	CDI89416.1	Pseudomonas aeruginosa PA38182
575872221	CDI89400.1	Pseudomonas aeruginosa PA38182
152983543	YP_001349476.1	Pseudomonas aeruginosa PA7
152986562	YP_001349491.1	Pseudomonas aeruginosa PA7
602743287	EYU03944.1	Pseudomonas aeruginosa PA99
15596465	NP_249959.1	Pseudomonas aeruginosa PAO1
15596452	NP_249946.1	Pseudomonas aeruginosa PAO1
514408753	YP_008131812.1	Pseudomonas aeruginosa RP73
568310880	YP_008983149.1	Pseudomonas aeruginosa SCV20265
568310894	YP_008983163.1	Pseudomonas aeruginosa SCV20265
116049220	YP_791977.1	Pseudomonas aeruginosa UCBPP-PA14
116049207	YP_791990.1	Pseudomonas aeruginosa UCBPP-PA14
582070297	EVT86711.1	Pseudomonas aeruginosa VRFPA09
576905555	AHH50045.1	Pseudomonas aeruginosa YL84
515700924	WP_017133524.1	Pseudomonas agarici
515700935	WP_017133535.1	Pseudomonas agarici
544803569	WP_021220726.1	Pseudomonas alcaligenes
545465497	WP_021702091.1	Pseudomonas alcaligenes
545465485	WP_021702079.1	Pseudomonas alcaligenes
591393066	AHL33959.1	Pseudomonas brassicacearum
591393057	AHL33950.1	Pseudomonas brassicacearum
330810048	YP_004354510.1	Pseudomonas brassicacearum subsp. brassicacearum NFM421

330810500	YP_004354962.1	Pseudomonas brassicacearum subsp. brassicacearum NFM421
330810058	YP_004354520.1	Pseudomonas brassicacearum subsp. brassicacearum NFM421
496340366	WP_009049544.1	Pseudomonas chlororaphis
515073197	WP_016703035.1	Pseudomonas chlororaphis
496333375	WP_009042553.1	Pseudomonas chlororaphis
565886961	WP_023968128.1	Pseudomonas chlororaphis
515072265	WP_016702110.1	Pseudomonas chlororaphis
496338282	WP_009047460.1	Pseudomonas chlororaphis
472323930	YP_007656176.1	Pseudomonas denitrificans ATCC 13867
472323918	YP_007656164.1	Pseudomonas denitrificans ATCC 13867
104783032	YP_609530.1	Pseudomonas entomophila L48
498248596	WP_010562752.1	Pseudomonas extremaustralis
489317022	WP_003224386.1	Pseudomonas fluorescens
489266360	WP_003174162.1	Pseudomonas fluorescens
489283948	WP_003191554.1	Pseudomonas fluorescens
515545021	WP_016978039.1	Pseudomonas fluorescens
515554799	WP_016987711.1	Pseudomonas fluorescens
515706272	WP_017138872.1	Pseudomonas fluorescens
489274710	WP_003182410.1	Pseudomonas fluorescens
489322856	WP_003230172.1	Pseudomonas fluorescens
517924613	WP_019094821.1	Pseudomonas fluorescens
516099961	WP_017530541.1	Pseudomonas fluorescens
489312607	WP_003220004.1	Pseudomonas fluorescens
489274728	WP_003182428.1	Pseudomonas fluorescens
489295047	WP_003202563.1	Pseudomonas fluorescens
387893998	YP_006324295.1	Pseudomonas fluorescens A506
378951638	YP_005209126.1	Pseudomonas fluorescens F113
378951628	YP_005209116.1	Pseudomonas fluorescens F113
77458535	YP_348040.1	Pseudomonas fluorescens Pf0-1
229590776	YP_002872895.1	Pseudomonas fluorescens SBW25
515151476	WP_016780118.1	Pseudomonas fragi
498342793	WP_010656949.1	Pseudomonas fragi
498342450	WP_010656606.1	Pseudomonas fragi
515152443	WP_016781079.1	Pseudomonas fragi
333902591	YP_004476464.1	Pseudomonas fulva 12-X
516515561	WP_017903999.1	Pseudomonas fuscovaginae
498136641	WP_010450797.1	Pseudomonas fuscovaginae
518191714	WP_019361922.1	Pseudomonas fuscovaginae
516518076	WP_017906442.1	Pseudomonas fuscovaginae
498136659	WP_010450815.1	Pseudomonas fuscovaginae
518191706	WP_019361914.1	Pseudomonas fuscovaginae
516518086	WP_017906452.1	Pseudomonas fuscovaginae
518194078	WP_019364286.1	Pseudomonas luteola
498146125	WP_010460281.1	Pseudomonas mandelii
518411751	WP_019581958.1	Pseudomonas mandelii
518411742	WP_019581949.1	Pseudomonas mandelii
568183496	YP_008956166.1	Pseudomonas monteilii SB3078
568183487	YP_008956156.1	Pseudomonas monteilii SB3078
566143709	WP_024012524.1	Pseudomonas moraviensis
516091145	WP_017521725.1	Pseudomonas nitroreducens
516091155	WP_017521735.1	Pseudomonas nitroreducens
551345216	WP_022964628.1	Pseudomonas pelagia
511758402	WP_016391646.1	Pseudomonas plecoglossicida
447916626	YP_007397194.1	Pseudomonas poae RE*1-1-14
610525409	BAO60813.1	Pseudomonas protegens Cab57
501678917	YP_007998743.1	Pseudomonas protegens CHA0
70728792	YP_258541.1	Pseudomonas protegens Pf-5
489547451	WP_003452093.1	Pseudomonas pseudoalcaligenes
494277901	WP_007160066.1	Pseudomonas psychrotolerans
545906582	WP_021783359.1	Pseudomonas putida
552950071	WP_023047730.1	Pseudomonas putida
519037212	WP_020193087.1	Pseudomonas putida
489349373	WP_003256469.1	Pseudomonas putida
490712896	WP_004575725.1	Pseudomonas putida
489347586	WP_003254707.1	Pseudomonas putida
496902298	WP_009403640.1	Pseudomonas putida
518266595	WP_019436803.1	Pseudomonas putida
518580460	WP_019750667.1	Pseudomonas putida
386010902	YP_005929179.1	Pseudomonas putida BIRD-1
397695896	YP_006533779.1	Pseudomonas putida DOT-T1E
148546528	YP_001266630.1	Pseudomonas putida F1
167035128	YP_001670359.1	Pseudomonas putida GB-1
515082271	WP_016711959.1	Pseudomonas putida group
512579100	YP_008096779.1	Pseudomonas putida H8234
431803882	YP_007230785.1	Pseudomonas putida HB3267
431803873	YP_007230776.1	Pseudomonas putida HB3267
26987993	NP_743418.1	Pseudomonas putida KT2440
512684254	YP_008112234.1	Pseudomonas putida NBRC 14164
395447792	YP_006388045.1	Pseudomonas putida ND6
339488866	YP_004703394.1	Pseudomonas putida S16
339488857	YP_004703385.1	Pseudomonas putida S16
170722486	YP_001750174.1	Pseudomonas putida W619
516238075	WP_017642038.1	Pseudomonas sp. 313
516235245	WP_017639208.1	Pseudomonas sp. 313
518479335	WP_019649542.1	Pseudomonas sp. 45MFCol3.1
518479342	WP_019649549.1	Pseudomonas sp. 45MFCol3.1
495714153	WP_008438732.1	Pseudomonas sp. Ag1
516346064	WP_017736097.1	Pseudomonas sp. CBZ-4
518665882	WP_019827589.1	Pseudomonas sp. CF149
518665547	WP_019827258.1	Pseudomonas sp. CF149
520825317	WP_020302724.1	Pseudomonas sp. CF150
520814343	WP_020300439.1	Pseudomonas sp. CF161
520803643	WP_020296190.1	Pseudomonas sp. CF161
520784119	WP_020289105.1	Pseudomonas sp. CFII64
520784099	WP_020289095.1	Pseudomonas sp. CFII64
517438727	WP_018609601.1	Pseudomonas sp. CFII68

518653739	WP_019817118.1	Pseudomonas sp. CFT9
550709408	WP_022643720.1	Pseudomonas sp. CMAA1215
550707986	WP_022642835.1	Pseudomonas sp. CMAA1215
568241179	AHD15901.1	Pseudomonas sp. FGI182
567638748	ETK14293.1	Pseudomonas sp. FH1
567645198	ETK20539.1	Pseudomonas sp. FH4
523666450	WP_020797790.1	Pseudomonas sp. G5(2012)
523666440	WP_020797780.1	Pseudomonas sp. G5(2012)
495171122	WP_007895918.1	Pseudomonas sp. GM102
495176108	WP_007900902.1	Pseudomonas sp. GM102
495178924	WP_007903714.1	Pseudomonas sp. GM102
495197950	WP_007922739.1	Pseudomonas sp. GM17
495210725	WP_007935505.1	Pseudomonas sp. GM18
495209002	WP_007933782.1	Pseudomonas sp. GM18
495210712	WP_007935492.1	Pseudomonas sp. GM18
495222158	WP_007946932.1	Pseudomonas sp. GM21
495217836	WP_007942613.1	Pseudomonas sp. GM21
495219535	WP_007944310.1	Pseudomonas sp. GM21
495233351	WP_007958122.1	Pseudomonas sp. GM25
495244496	WP_007969259.1	Pseudomonas sp. GM30
495254740	WP_007979495.1	Pseudomonas sp. GM33
495255775	WP_007980530.1	Pseudomonas sp. GM33
495254758	WP_007979513.1	Pseudomonas sp. GM33
495432514	WP_008157209.1	Pseudomonas sp. GM41(2012)
495432504	WP_008157199.1	Pseudomonas sp. GM41(2012)
495265783	WP_007990538.1	Pseudomonas sp. GM48
495269559	WP_007994314.1	Pseudomonas sp. GM49
495272975	WP_007997730.1	Pseudomonas sp. GM49
495287037	WP_008011791.1	Pseudomonas sp. GM50
495288297	WP_008013051.1	Pseudomonas sp. GM50
495282534	WP_008007288.1	Pseudomonas sp. GM50
495296792	WP_008021545.1	Pseudomonas sp. GM55
495293903	WP_008018656.1	Pseudomonas sp. GM55
495293892	WP_008018645.1	Pseudomonas sp. GM55
495308642	WP_008033391.1	Pseudomonas sp. GM60
495304883	WP_008029633.1	Pseudomonas sp. GM60
495299893	WP_008024643.1	Pseudomonas sp. GM60
495314052	WP_008038800.1	Pseudomonas sp. GM67
495317870	WP_008042617.1	Pseudomonas sp. GM67
495317881	WP_008042628.1	Pseudomonas sp. GM67
495328004	WP_008052746.1	Pseudomonas sp. GM74
495328650	WP_008053391.1	Pseudomonas sp. GM74
495339731	WP_008064465.1	Pseudomonas sp. GM78
495335014	WP_008059751.1	Pseudomonas sp. GM78
495335033	WP_008059770.1	Pseudomonas sp. GM78
495350657	WP_008075382.1	Pseudomonas sp. GM79
495350660	WP_008075385.1	Pseudomonas sp. GM79
495348428	WP_008073155.1	Pseudomonas sp. GM79
495349966	WP_008074692.1	Pseudomonas sp. GM79
495347003	WP_008071733.1	Pseudomonas sp. GM79
495363981	WP_008088699.1	Pseudomonas sp. GM80
495375929	WP_008100641.1	Pseudomonas sp. GM84
498494741	WP_010795476.1	Pseudomonas sp. HPB0071
497909715	WP_010223871.1	Pseudomonas sp. HYS
497909730	WP_010223886.1	Pseudomonas sp. HYS
489534754	WP_003439482.1	Pseudomonas sp. Lz4W
489540491	WP_003445199.1	Pseudomonas sp. Lz4W
497308366	WP_009622583.1	Pseudomonas sp. M1
497305531	WP_009619748.1	Pseudomonas sp. M1
497308372	WP_009622589.1	Pseudomonas sp. M1
497305337	WP_009619554.1	Pseudomonas sp. M1
495644857	WP_008369436.1	Pseudomonas sp. M47T1
498493189	WP_010793943.1	Pseudomonas sp. P179
497856764	WP_010170920.1	Pseudomonas sp. PAMC 25886
497861533	WP_010175689.1	Pseudomonas sp. PAMC 25886
516048232	WP_017478815.1	Pseudomonas sp. PAMC 26793
612067553	EZP26587.1	Pseudomonas sp. RIT288
612066672	EZP25710.1	Pseudomonas sp. RIT288
518301868	WP_019472076.1	Pseudomonas sp. S13.1.2
497369102	WP_009683315.1	Pseudomonas sp. TJI-51
568140473	YP_008933058.1	Pseudomonas sp. TKP
426409928	YP_007030027.1	Pseudomonas sp. UW4
557224393	YP_008764608.1	Pseudomonas sp. VLB120
594026361	AHL74364.1	Pseudomonas stutzeri
594025756	AHL73759.1	Pseudomonas stutzeri
594025746	AHL73749.1	Pseudomonas stutzeri
492246215	WP_005788088.1	Pseudomonas synxantha
520893460	WP_020319541.1	Pseudomonas syringae
516297816	WP_017701123.1	Pseudomonas syringae
515540463	WP_016973516.1	Pseudomonas tolaasii
515538792	WP_016971845.1	Pseudomonas tolaasii
516457507	WP_017846347.1	Pseudomonas veronii
543350687	XP_005520425.1	Pseudopodoces humilis
493645849	WP_006597464.1	Pseudoramibacter alactolyticus
374332973	YP_005083157.1	Pseudovibrio sp. FO-BEG1
374329157	YP_005079341.1	Pseudovibrio sp. FO-BEG1
374330175	YP_005080359.1	Pseudovibrio sp. FO-BEG1
495827076	WP_008551655.1	Pseudovibrio sp. JE062
495823869	WP_008548448.1	Pseudovibrio sp. JE062
495823995	WP_008548574.1	Pseudovibrio sp. JE062
495823762	WP_008548341.1	Pseudovibrio sp. JE062
93006047	YP_580484.1	Psychrobacter cryohalolentis K5
518501632	WP_019671839.1	Psychrobacter lutiphocae
521179403	YP_008163148.1	Psychrobacter sp. G
589899391	GAF53322.1	Psychrobacter sp. JCM 18900
589904241	GAF62729.1	Psychrobacter sp. JCM 18903
408491963	YP_006868332.1	Psychroflexus torquis ATCC 700755
119945781	YP_943461.1	Psychromonas ingrahamii 37
586530569	XP_006920194.1	Pteropus alecto
599100244	XP_007380862.1	Punctularia strigosozonata HHB-11173 SS5
548381775	XP_005734442.1	Pundamilia nyererei
602643426	XP_007444944.1	Python bivittatus

344175960	CCA87108.1	Ralstonia syzygii R24
344171589	CCA84206.1	Ralstonia syzygii R24
344171603	CCA84221.1	Ralstonia syzygii R24
344171595	CCA84212.1	Ralstonia syzygii R24
481850892	YP_007875332.1	Raoultella ornithinolytica B6
157820257	NP_001101501.1	Rattus norvegicus
495320085	WP_008044831.1	Reinekea blandensis
163840560	YP_001624965.1	Renibacterium salmoninarum ATCC 33209
569426516	ETO31383.1	Reticulomyxa filosa
496173083	WP_008897590.1	Rheinheimera sp. A13L
528827297	YP_008363253.1	Rhizobium etli bv. mimosae str. Mim1
528834336	YP_008367990.1	Rhizobium etli bv. mimosae str. Mim1
528834315	YP_008367969.1	Rhizobium etli bv. mimosae str. Mim1
86361121	YP_473008.1	Rhizobium etli CFN 42
86356108	YP_468000.1	Rhizobium etli CFN 42
190894907	YP_001985200.1	Rhizobium etli CIAT 652
190894973	YP_001985266.1	Rhizobium etli CIAT 652
190894981	YP_001985274.1	Rhizobium etli CIAT 652
190894978	YP_001985271.1	Rhizobium etli CIAT 652
190890148	YP_001976690.1	Rhizobium etli CIAT 652
327193267	EGE60173.1	Rhizobium etli CNPAF512
327192642	EGE59582.1	Rhizobium etli CNPAF512
490222574	WP_004120939.1	Rhizobium freirei
490220906	WP_004119278.1	Rhizobium freirei
490211825	WP_004110222.1	Rhizobium freirei
517258896	WP_018447714.1	Rhizobium gallicum
517256302	WP_018445120.1	Rhizobium gallicum
517135168	WP_018323986.1	Rhizobium giardinii
517138767	WP_018327585.1	Rhizobium giardinii
517136591	WP_018325409.1	Rhizobium giardinii
517137376	WP_018326194.1	Rhizobium giardinii
514391678	WP_016552564.1	Rhizobium grahamii
514391281	WP_016552169.1	Rhizobium grahamii
516581806	WP_017956845.1	Rhizobium leguminosarum
489686888	WP_003591100.1	Rhizobium leguminosarum
518891458	WP_020047333.1	Rhizobium leguminosarum
517057204	WP_018246022.1	Rhizobium leguminosarum
516731280	WP_018071459.1	Rhizobium leguminosarum
489646615	WP_003551055.1	Rhizobium leguminosarum
516616728	WP_017991571.1	Rhizobium leguminosarum
516621122	WP_017995909.1	Rhizobium leguminosarum
489640602	WP_003545042.1	Rhizobium leguminosarum
516615292	WP_017990135.1	Rhizobium leguminosarum
516621196	WP_017995983.1	Rhizobium leguminosarum
517055759	WP_018244577.1	Rhizobium leguminosarum
516583740	WP_017958779.1	Rhizobium leguminosarum
517306812	WP_018495630.1	Rhizobium leguminosarum
517292512	WP_018481330.1	Rhizobium leguminosarum
516591771	WP_017966810.1	Rhizobium leguminosarum
518892392	WP_020048267.1	Rhizobium leguminosarum
516727622	WP_018068756.1	Rhizobium leguminosarum
517305430	WP_018494248.1	Rhizobium leguminosarum
489664448	WP_003568744.1	Rhizobium leguminosarum
489688054	WP_003592261.1	Rhizobium leguminosarum
489671364	WP_003575620.1	Rhizobium leguminosarum
489684644	WP_003588866.1	Rhizobium leguminosarum
489679699	WP_003583924.1	Rhizobium leguminosarum
489666410	WP_003570696.1	Rhizobium leguminosarum
516582115	WP_017957154.1	Rhizobium leguminosarum
489676699	WP_003580937.1	Rhizobium leguminosarum
489689093	WP_003593293.1	Rhizobium leguminosarum
489660817	WP_003565142.1	Rhizobium leguminosarum
574594220	AHG49378.1	Rhizobium leguminosarum bv. trifolii CB782
574588713	AHG43872.1	Rhizobium leguminosarum bv. trifolii CB782
241202872	YP_002973968.1	Rhizobium leguminosarum bv. trifolii WSM1325
241113196	YP_002973031.1	Rhizobium leguminosarum bv. trifolii WSM1325
573465350	AHF82232.1	Rhizobium leguminosarum bv. trifolii WSM1689
209547289	YP_002279207.1	Rhizobium leguminosarum bv. trifolii WSM2304
209546906	YP_002278824.1	Rhizobium leguminosarum bv. trifolii WSM2304
209547718	YP_002279635.1	Rhizobium leguminosarum bv. trifolii WSM2304
209550221	YP_002282138.1	Rhizobium leguminosarum bv. trifolii WSM2304
116250250	YP_766088.1	Rhizobium leguminosarum bv. viciae 3841
116249199	YP_765040.1	Rhizobium leguminosarum bv. viciae 3841
490574680	WP_004439700.1	Rhizobium lupini
494803496	WP_007538904.1	Rhizobium mesoamericanum
494794269	WP_007529677.1	Rhizobium mesoamericanum
494793428	WP_007528836.1	Rhizobium mesoamericanum
550964796	WP_022713107.1	Rhizobium mongolense
550965228	WP_022713538.1	Rhizobium mongolense
515105800	WP_016734918.1	Rhizobium phaseoli
430002682	CCF18463.1	Rhizobium sp.
430005201	CCF21002.1	Rhizobium sp.
517728049	WP_018898257.1	Rhizobium sp. 2MFCol3.1
517727654	WP_018897862.1	Rhizobium sp. 2MFCol3.1
517687486	WP_018857694.1	Rhizobium sp. 42MFCr.1
517689533	WP_018859741.1	Rhizobium sp. 42MFCr.1
494974325	WP_007700350.1	Rhizobium sp. AP16
494969481	WP_007695507.1	Rhizobium sp. AP16
517048650	WP_018237468.1	Rhizobium sp. BR816
517047472	WP_018236290.1	Rhizobium sp. BR816
517046152	WP_018234970.1	Rhizobium sp. BR816
517050650	WP_018239468.1	Rhizobium sp. BR816
517048647	WP_018237465.1	Rhizobium sp. BR816
517047469	WP_018236287.1	Rhizobium sp. BR816
517047859	WP_018236677.1	Rhizobium sp. BR816
494907625	WP_007633666.1	Rhizobium sp. CCGE 510
494909683	WP_007635724.1	Rhizobium sp. CCGE 510

494909686	WP_007635727.1	Rhizobium sp. CCGE 510
495041305	WP_007766157.1	Rhizobium sp. CF080
495031625	WP_007757171.1	Rhizobium sp. CF080
495076538	WP_007801363.1	Rhizobium sp. CF122
495077159	WP_007801984.1	Rhizobium sp. CF122
495074260	WP_007799085.1	Rhizobium sp. CF122
495094707	WP_007819530.1	Rhizobium sp. CF142
495096595	WP_007821418.1	Rhizobium sp. CF142
549704805	YP_008631347.1	Rhizobium sp. IRBG74
584451312	CDM56216.1	Rhizobium sp. LPU83
584452558	CDM57462.1	Rhizobium sp. LPU83
584450862	CDM55766.1	Rhizobium sp. LPU83
494871321	WP_007597417.1	Rhizobium sp. PDO1-076
494880862	WP_007606912.1	Rhizobium sp. PDO1-076
494881694	WP_007607744.1	Rhizobium sp. PDO1-076
495807607	WP_008532186.1	Rhizobium sp. Pop5
495807509	WP_008532088.1	Rhizobium sp. Pop5
495799823	WP_008524402.1	Rhizobium sp. Pop5
440225406	YP_007332497.1	Rhizobium tropici CIAT 899
552930421	ESA14159.1	Rhizophagus irregularis DAOM 181602
495491279	WP_008215961.1	Rhodanobacter sp. 115
495083463	WP_007808287.1	Rhodanobacter spathiphylli
488811975	WP_002724381.1	Rhodobacter
563378295	WP_023663921.1	Rhodobacter sp. CACIA14H1
563384607	WP_023666870.1	Rhodobacter sp. CACIA14H1
77465520	YP_355023.1	Rhodobacter sphaeroides 2.4.1
126463923	YP_001045036.1	Rhodobacter sphaeroides ATCC 17029
549985409	WP_022574205.1	Rhodobacteraceae bacterium HIMB11
495459172	WP_008183864.1	Rhodobacteraceae bacterium HTCC2150
496033893	WP_008758400.1	Rhodobacteraceae bacterium KLH11
496034388	WP_008758895.1	Rhodobacteraceae bacterium KLH11
495309255	WP_008034003.1	Rhodobacterales bacterium HTCC2255
495830385	WP_008554964.1	Rhodobacterales bacterium Y4I
494511356	WP_007300814.1	Rhodococcus imtechensis
111022255	YP_705227.1	Rhodococcus jostii RHA1
111021538	YP_704510.1	Rhodococcus jostii RHA1
491385683	WP_005243564.1	Rhodococcus opacus
491386864	WP_005244742.1	Rhodococcus opacus
491396575	WP_005254426.1	Rhodococcus opacus
589064187	AHK29046.1	Rhodococcus opacus PD630
589063393	AHK28252.1	Rhodococcus opacus PD630
515394752	WP_016884067.1	Rhodococcus sp. DK17
497119656	WP_009477627.1	Rhodococcus sp. JVH1
491740765	WP_005573898.1	Rhodococcus wratislaviensis
589256081	GAF49764.1	Rhodococcus wratislaviensis NBRC 100605
518426522	WP_019596729.1	Rhodonellum psychrophilum
494561256	WP_007336000.1	Rhodopirellula baltica
494554619	WP_007333096.1	Rhodopirellula baltica
494535280	WP_007324728.1	Rhodopirellula baltica
32471735	NP_864728.1	Rhodopirellula baltica SH 1
495943052	WP_008667631.1	Rhodopirellula europaea
495932001	WP_008656580.1	Rhodopirellula europaea
495966206	WP_008690785.1	Rhodopirellula maiorica
495979803	WP_008704382.1	Rhodopirellula maiorica
496384760	WP_009093750.1	Rhodopirellula sp. SWK7
83593400	YP_427152.1	Rhodospirillum rubrum ATCC 11170
268316069	YP_003289788.1	Rhodothermus marinus DSM 4252
260063309	YP_003196389.1	Robiginitalea biformata HTCC2501
594548830	AHM03849.1	Roseibacterium elongatum DSM 19469
497445520	WP_009759718.1	Roseibium sp. TrichSKD4
497098346	WP_009468953.1	Roseibium sp. TrichSKD4
156740256	YP_001430385.1	Roseiflexus castenholzii DSM 13941
148654807	YP_001275012.1	Roseiflexus sp. RS-1
495089301	WP_007814124.1	Roseobacter sp. AzwK-3b
497495015	WP_009809213.1	Roseobacter sp. MED193
497495242	WP_009809440.1	Roseobacter sp. MED193
495481394	WP_008206081.1	Roseobacter sp. SK209-2-6
495482789	WP_008207476.1	Roseobacter sp. SK209-2-6
518293134	WP_019463342.1	Roseomonas sp. B5
497499475	WP_009813673.1	Roseovarius nubinhibens
497503929	WP_009818127.1	Roseovarius sp. 217
497503931	WP_009818129.1	Roseovarius sp. 217
495557419	WP_008281998.1	Roseovarius sp. TM1035
495557424	WP_008282003.1	Roseovarius sp. TM1035
598664302	EYD76914.1	Rubellimicrobium mesophilum DSM 19309
598664516	EYD77094.1	Rubellimicrobium mesophilum DSM 19309
544663070	WP_021096441.1	Rubellimicrobium thermophilum
108805064	YP_645001.1	Rubrobacter xylanophilus DSM 9941
108805058	YP_644995.1	Rubrobacter xylanophilus DSM 9941
517803453	WP_018973661.1	Rudaea cellulosilytica
518830807	WP_019986752.1	Rudanella lutea
498127361	WP_010441517.1	Ruegeria conchae
498127482	WP_010441638.1	Ruegeria conchae
492822357	WP_005977104.1	Ruegeria lacuscaerulensis
492822349	WP_005977100.1	Ruegeria lacuscaerulensis
491814983	WP_005614887.1	Ruegeria mobilis
491814989	WP_005614891.1	Ruegeria mobilis
56695519	YP_165867.1	Ruegeria pomeroyi DSS-3
56709050	YP_165095.1	Ruegeria pomeroyi DSS-3
495838534	WP_008563113.1	Ruegeria sp. R11
495836129	WP_008560708.1	Ruegeria sp. R11
99078179	YP_611437.1	Ruegeria sp. TM1040
99078177	YP_611435.1	Ruegeria sp. TM1040
338212056	YP_004656111.1	Runella slithyformis DSM 19594
338213242	YP_004657297.1	Runella slithyformis DSM

		19594
491597206	WP_005454768.1	Saccharomonospora cyanea
493279385	WP_006237162.1	Saccharomonospora xinjiangensis
134099791	YP_001105452.1	Saccharopolyspora erythraea NRRL 2338
498382961	WP_010697117.1	Saccharopolyspora spinosa
498000918	WP_010315074.1	Saccharopolyspora spinosa
291228440	XP_002734190.1	Saccoglossus kowalevskii
585657116	XP_002734182.2	Saccoglossus kowalevskii
585657177	XP_002734189.2	Saccoglossus kowalevskii
585657120	XP_006815791.1	Saccoglossus kowalevskii
291238478	XP_002739157.1	Saccoglossus kowalevskii
403264330	XP_003924439.1	Saimiri boliviensis boliviensis
518850425	WP_020006315.1	Salinicoccus albus
516119230	WP_017549810.1	Salinicoccus carnicancri
493968979	WP_006912223.1	Salinisphaera shabanensis
517752843	WP_018923051.1	Salsuginibacillus kocurii
517752849	WP_018923057.1	Salsuginibacillus kocurii
395510205	XP_003759371.1	Sarcophilus harrisii
518373066	WP_019543273.1	Selenomonas bovis
546164529	WP_021806387.1	Serratia fonticola
544751053	WP_021177968.1	Serratia fonticola
573927192	AHG18489.1	Serratia fonticola RB-25
525691776	YP_008229666.1	Serratia liquefaciens ATCC 27592
518286249	WP_019456457.1	Serratia marcescens
515494995	WP_016928249.1	Serratia marcescens
491066341	WP_004927972.1	Serratia marcescens
575538069	ETX38514.1	Serratia marcescens BIDMC 44
575548630	ETX49034.1	Serratia marcescens BIDMC 50
612279760	EZQ73149.1	Serratia marcescens BIDMC 80
440230439	YP_007344232.1	Serratia marcescens FGI94
573010003	BAO33355.1	Serratia marcescens SM39
560172935	CDG11664.1	Serratia marcescens subsp. marcescens Db11
448241558	YP_007405611.1	Serratia marcescens WW4
493365285	WP_006321706.1	Serratia plymuthica
518648315	YP_008137855.1	Serratia plymuthica 4Rx13
157370082	YP_001478071.1	Serratia proteamaculans 568
333926646	YP_004500225.1	Serratia sp. AS12
558558053	WP_023489100.1	Serratia sp. DD3
516503932	WP_017892370.1	Serratia sp. S4
482812460	EOA89179.1	Setosphaeria turcica Et28A
119774667	YP_927407.1	Shewanella amazonensis SB2B
119774669	YP_927409.1	Shewanella amazonensis SB2B
491646029	WP_005503555.1	Shewanella benthica
167624477	YP_001674771.1	Shewanella halifaxensis HAW-EB4
167624475	YP_001674769.1	Shewanella halifaxensis HAW-EB4
127513291	YP_001094488.1	Shewanella loihica PV-4
127513289	YP_001094486.1	Shewanella loihica PV-4
157961528	YP_001501562.1	Shewanella pealeana ATCC 700345

157961530	YP_001501564.1	Shewanella pealeana ATCC 700345
212634802	YP_002311327.1	Shewanella piezotolerans WP3
212634804	YP_002311329.1	Shewanella piezotolerans WP3
157374900	YP_001473500.1	Shewanella sediminis HAW-EB3
157374902	YP_001473502.1	Shewanella sediminis HAW-EB3
294140480	YP_003556458.1	Shewanella violacea DSS12
294140482	YP_003556460.1	Shewanella violacea DSS12
170727167	YP_001761193.1	Shewanella woodyi ATCC 51908
170727165	YP_001761191.1	Shewanella woodyi ATCC 51908
558646103	WP_023516362.1	Shinella zoogloeoides
558640804	WP_023512445.1	Shinella zoogloeoides
558641826	WP_023513074.1	Shinella zoogloeoides
496466775	WP_009175620.1	Silicibacter sp. TrichCH4B
496466892	WP_009175737.1	Silicibacter sp. TrichCH4B
410663331	YP_006915702.1	Simiduia agarivorans SA1 = DSM 21679
410663325	YP_006915696.1	Simiduia agarivorans SA1 = DSM 21679
378824695	YP_005187427.1	Sinorhizobium fredii HH103
378763080	YP_005191696.1	Sinorhizobium fredii HH103
378827610	YP_005190342.1	Sinorhizobium fredii HH103
227820687	YP_002824657.1	Sinorhizobium fredii NGR234
227823541	YP_002827514.1	Sinorhizobium fredii NGR234
227823538	YP_002827511.1	Sinorhizobium fredii NGR234
398352454	YP_006397918.1	Sinorhizobium fredii USDA 257
398349992	YP_006395456.1	Sinorhizobium fredii USDA 257
398352457	YP_006397921.1	Sinorhizobium fredii USDA 257
516638836	WP_018011067.1	Sinorhizobium medicae
150376012	YP_001312608.1	Sinorhizobium medicae WSM419
150376010	YP_001312606.1	Sinorhizobium medicae WSM419
489624145	WP_003528585.1	Sinorhizobium meliloti
516780375	WP_018097992.1	Sinorhizobium meliloti
515842959	WP_017273712.1	Sinorhizobium meliloti
515835789	WP_017266542.1	Sinorhizobium meliloti
516780371	WP_018097990.1	Sinorhizobium meliloti
489624136	WP_003528576.1	Sinorhizobium meliloti
515840220	WP_017270973.1	Sinorhizobium meliloti
515838837	WP_017269590.1	Sinorhizobium meliloti
515846088	WP_017276841.1	Sinorhizobium meliloti
16264006	NP_436798.1	Sinorhizobium meliloti 1021
16264008	NP_436800.1	Sinorhizobium meliloti 1021
334319856	YP_004556485.1	Sinorhizobium meliloti AK83
334319854	YP_004556483.1	Sinorhizobium meliloti AK83
384533839	YP_005716503.1	Sinorhizobium meliloti BL225C
433611570	YP_007195031.1	Sinorhizobium meliloti GR4
433611568	YP_007195029.1	Sinorhizobium meliloti GR4
407690360	YP_006813944.1	Sinorhizobium meliloti Rm41
407724017	YP_006843678.1	Sinorhizobium meliloti Rm41
384540222	YP_005724305.1	Sinorhizobium meliloti SM11
521466385	YP_008153690.1	Sorangium cellulosum So0157-

		2
505774137	XP_004601842.1	Sorex araneus
269929404	YP_003321725.1	Sphaerobacter thermophilus DSM 20745
326798411	YP_004316230.1	Sphingobacterium sp. 21
494984829	WP_007710852.1	Sphingobium sp. AP49
347528197	YP_004834944.1	Sphingobium sp. SYK-6
347528215	YP_004834962.1	Sphingobium sp. SYK-6
490323009	WP_004212494.1	Sphingobium yanoikuyae
498024975	WP_010339131.1	Sphingobium yanoikuyae
498088119	WP_010402275.1	Sphingomonas echinoides
498230343	WP_010544499.1	Sphingomonas elodea
550938503	WP_022686908.1	Sphingomonas phyllosphaerae
569543856	AHE55473.1	Sphingomonas sanxanigenens DSM 19645 = NX02
518197947	WP_019368155.1	Sphingomonas sp. ATCC 31555
518671918	WP_019833611.1	Sphingomonas sp. PR090111-T3T-6A
612091387	EZP49906.1	Sphingomonas sp. RIT328
494648114	WP_007406058.1	Sphingomonas sp. S17
507525032	YP_008046645.1	Spiribacter salinus M19-40
556585926	YP_008753497.1	Spiribacter sp. UAH-SP71
556585923	YP_008753494.1	Spiribacter sp. UAH-SP71
556586897	YP_008754493.1	Spiribacter sp. UAH-SP71
563693667	YP_008868650.1	Spiroplasma apis B31
284036397	YP_003386327.1	Spirosoma linguale DSM 74
517447570	WP_018618395.1	Spirosoma luteum
522085653	WP_020596862.1	Spirosoma panaciterrae
522091451	WP_020602660.1	Spirosoma spitsbergense
497711481	WP_010025665.1	Sporolactobacillus inulinus
291301577	YP_003512855.1	Stackebrandtia nassauensis DSM 44728
515567843	WP_017000674.1	Staphylococcus lentus
545089756	WP_021459396.1	Staphylococcus sp. EGD-HP3
298290173	YP_003692112.1	Starkeya novella DSM 506
298293153	YP_003695092.1	Starkeya novella DSM 506
298290171	YP_003692110.1	Starkeya novella DSM 506
517673060	WP_018843268.1	Streptomyces
493086431	WP_006128209.1	Streptomyces
517348261	WP_018523753.1	Streptomyces
516785475	WP_018100590.1	Streptomyces
498043940	WP_010358096.1	Streptomyces acidiscabies
519334462	WP_020273487.1	Streptomyces afghaniensis
514437397	WP_016576245.1	Streptomyces albulus
514923904	WP_016644995.1	Streptomyces aurantiacus
514921553	WP_016643239.1	Streptomyces aurantiacus
493655227	WP_006606673.1	Streptomyces auratus
493656664	WP_006608087.1	Streptomyces auratus
29828272	NP_822906.1	Streptomyces avermitilis MA-4680 = NBRC 14893
374985906	YP_004961401.1	Streptomyces bingchenggensis BCW-1
491627009	WP_005484548.1	Streptomyces bottropensis
518963077	WP_020118952.1	Streptomyces canus
357408815	YP_004920738.1	Streptomyces cattleya NRRL
		8057 = DSM 46488
497728802	WP_010042986.1	Streptomyces chartreusis
494709670	WP_007445570.1	Streptomyces coelicoflavus
21224612	NP_630391.1	Streptomyces coelicolor A3(2)
529229036	YP_008389857.1	Streptomyces collinus Tu 365
471321237	YP_007520267.1	Streptomyces davawensis JCM 4913
493086432	WP_006128210.1	Streptomyces filamentosus
497758253	WP_010072437.1	Streptomyces filamentosus
488613282	YP_007934618.1	Streptomyces fulvissimus DSM 40593
497745372	WP_010059556.1	Streptomyces globisporus
491060520	WP_004922154.1	Streptomyces griseoflavus
490063443	WP_003965665.1	Streptomyces griseus
490063442	WP_003965664.1	Streptomyces griseus
182435418	YP_001823137.1	Streptomyces griseus subsp. griseus NBRC 13350
182435417	YP_001823136.1	Streptomyces griseus subsp. griseus NBRC 13350
497406122	WP_009720320.1	Streptomyces himastatinicus
497398125	WP_009712338.1	Streptomyces himastatinicus
386843821	YP_006248879.1	Streptomyces hygroscopicus subsp. jinggangensis 5008
496681190	WP_009323388.1	Streptomyces ipomoeae
490070457	WP_003972632.1	Streptomyces lividans
491077338	WP_004938960.1	Streptomyces mobaraensis
558882160	WP_023537187.1	Streptomyces niveus
491451488	WP_005309272.1	Streptomyces pristinaespiralis
517888759	WP_019058967.1	Streptomyces prunicolor
518731293	WP_019891275.1	Streptomyces purpureus
557687802	YP_008791329.1	Streptomyces rapamycinicus NRRL 5491
490085238	WP_003987223.1	Streptomyces rimosus
558890515	WP_023545204.1	Streptomyces roseochromogenes
290955809	YP_003486991.1	Streptomyces scabiei 87.22
290963204	YP_003494386.1	Streptomyces scabiei 87.22
522039975	WP_020551184.1	Streptomyces scabrisporus
522040284	WP_020551493.1	Streptomyces scabrisporus
518969802	WP_020125677.1	Streptomyces sp. 303MFCol5.2
518977087	WP_020132962.1	Streptomyces sp. 303MFCol5.2
518977727	WP_020133602.1	Streptomyces sp. 351MFTsu5.1
518188288	WP_019358496.1	Streptomyces sp. AA1529
517380511	WP_018554707.1	Streptomyces sp. ATexAB-D23
517382965	WP_018556988.1	Streptomyces sp. BoleA5
494477675	WP_007267152.1	Streptomyces sp. C
494477676	WP_007267153.1	Streptomyces sp. C
517302968	WP_018491786.1	Streptomyces sp. CcalMP-8W
517786827	WP_018957035.1	Streptomyces sp. CNB091
517786826	WP_018957034.1	Streptomyces sp. CNB091
517669348	WP_018839556.1	Streptomyces sp. CNQ766
517667266	WP_018837474.1	Streptomyces sp. CNQ766
517674172	WP_018844380.1	Streptomyces sp. CNS335
517679906	WP_018850114.1	Streptomyces sp. CNT372
517683004	WP_018853212.1	Streptomyces sp. CNY243

516768732	WP_018091455.1	Streptomyces sp. FxanaC1
518354821	WP_019525028.1	Streptomyces sp. FxanaD5
512490516	WP_016431508.1	Streptomyces sp. HGB0020
512662528	WP_016468371.1	Streptomyces sp. HPH0547
517367992	WP_018543025.1	Streptomyces sp. MspMP-M5
606234199	EYU70218.1	Streptomyces sp. PCS3-D2
606234200	EYU70219.1	Streptomyces sp. PCS3-D2
594131223	EXU66480.1	Streptomyces sp. PRh5
517392285	WP_018565796.1	Streptomyces sp. PsTaAH-124
517392279	WP_018565791.1	Streptomyces sp. PsTaAH-124
517899630	WP_019069838.1	Streptomyces sp. R1-NS-10
517338886	WP_018514378.1	Streptomyces sp. ScaeMP-e10
515807133	WP_017237886.1	Streptomyces sp. SS
515807125	WP_017237878.1	Streptomyces sp. SS
602255307	EYT81321.1	Streptomyces sp. Tu 6176
494718702	WP_007454568.1	Streptomyces sp. W007
494718704	WP_007454570.1	Streptomyces sp. W007
518593056	WP_019763263.1	Streptomyces sp. Wigar10
494627648	WP_007385592.1	Streptomyces sviceus
559793776	WP_023590492.1	Streptomyces thermolilacinus
493425299	WP_006381023.1	Streptomyces turgidiscabies
408682862	YP_006882689.1	Streptomyces venezuelae ATCC 10712
408682851	YP_006882678.1	Streptomyces venezuelae ATCC 10712
345008090	YP_004810444.1	Streptomyces violaceusniger Tu 4113
490094445	WP_003996365.1	Streptomyces viridochromogenes
490092364	WP_003994285.1	Streptomyces viridochromogenes
490101518	WP_004003397.1	Streptomyces viridochromogenes
490101517	WP_004003396.1	Streptomyces viridochromogenes
494760226	WP_007495634.1	Streptomyces zinciresistens
271964986	YP_003339182.1	Streptosporangium roseum DSM 43021
271967285	YP_003341481.1	Streptosporangium roseum DSM 43021
271967278	YP_003341474.1	Streptosporangium roseum DSM 43021
72084055	XP_790644.1	Strongylocentrotus purpuratus
115749038	XP_787917.2	Strongylocentrotus purpuratus
512682141	WP_016474624.1	Sutterella wadsworthensis
495984971	WP_008709550.1	Synergistes sp. 3_1_syn1
495988575	WP_008713154.1	Synergistes sp. 3_1_syn1
495984871	WP_008709450.1	Synergistes sp. 3_1_syn1
495988506	WP_008713085.1	Synergistes sp. 3_1_syn1
495984881	WP_008709460.1	Synergistes sp. 3_1_syn1
551314574	WP_022934434.1	Synergistetes bacterium JGI 0000079-D21
49087102	AAT51417.1	synthetic construct
224051879	XP_002200408.1	Taeniopygia guttata
410926475	XP_003976704.1	Takifugu rubripes
212545827	XP_002153067.1	Talaromyces marneffei ATCC 18224
212537321	XP_002148816.1	Talaromyces marneffei ATCC 18224
242820552	XP_002487532.1	Talaromyces stipitatus ATCC 10500
47224622	CAG03606.1	Tetraodon nigroviridis
496449839	WP_009158684.1	Thalassobium sp. R2A62
550984117	WP_022732214.1	Thalassospira lucentensis
550984120	WP_022732217.1	Thalassospira lucentensis
496165586	WP_008890093.1	Thalassospira profundimaris
496165584	WP_008890091.1	Thalassospira profundimaris
494151768	WP_007091512.1	Thalassospira xiamenensis
494151766	WP_007091510.1	Thalassospira xiamenensis
410668354	YP_006920725.1	Thermacetogenium phaeum DSM 12270
269792074	YP_003316978.1	Thermanaerovibrio acidaminovorans DSM 6589
493632082	WP_006583961.1	Thermanaerovibrio velox
517493005	WP_018663582.1	Thermobrachium celere
530547731	YP_008428263.1	Thermococcus litoralis DSM 5473
530547727	YP_008428259.1	Thermococcus litoralis DSM 5473
242398620	YP_002994044.1	Thermococcus sibiricus MM 739
242398623	YP_002994047.1	Thermococcus sibiricus MM 739
573024906	AHF80440.1	Thermococcus sp. ES1
221635568	YP_002523444.1	Thermomicrobium roseum DSM 5159
302390215	YP_003826036.1	Thermosediminibacter oceani DSM 16646
357420410	YP_004933402.1	Thermovirga lienii DSM 17291
389879517	YP_006381747.1	Tistrella mobilis KA081020-065
392569653	EIW62826.1	Trametes versicolor FP-101664 SS1
471360799	XP_004370968.1	Trichechus manatus latirostris
358393051	EHK42452.1	Trichoderma atroviride IMI 206040
401886828	EJT50845.1	Trichosporon asahii var. asahii CBS 2479
406698779	EKD02006.1	Trichosporon asahii var. asahii CBS 8904
297623084	YP_003704518.1	Truepera radiovictrix DSM 17093
407832562	EKF98492.1	Trypanosoma cruzi
557861670	ESS65020.1	Trypanosoma cruzi Dm28c
407396786	EKF27546.1	Trypanosoma cruzi marinkellei
71419827	XP_811287.1	Trypanosoma cruzi strain CL Brener
71400794	XP_803162.1	Trypanosoma cruzi strain CL Brener
142933105	ABO92935.1	Trypanosoma vivax
562830761	XP_006144887.1	Tupaia chinensis
444728574	ELW69024.1	Tupaia chinensis
470631638	XP_004322013.1	Tursiops truncatus
519105615	WP_020261490.1	unclassified Aminicenantes
488681165	WP_002608780.1	unclassified Erysipelotrichaceae
406922004	EKD59665.1	uncultured bacterium
297181675	ADI17858.1	uncultured Rhodospirillales bacterium HF0200_01O14

297181678	ADI17861.1	uncultured Rhodospirillales bacterium HF0200_01O14
518483572	WP_019653779.1	Variovorax paradoxus
517733268	WP_018903476.1	Variovorax paradoxus
538397581	YP_008519154.1	Variovorax paradoxus B4
319796572	YP_004158212.1	Variovorax paradoxus EPS
239818178	YP_002947088.1	Variovorax paradoxus S110
495103808	WP_007828631.1	Variovorax sp. CF313
497788560	WP_010102744.1	Verminephrobacter aporrectodeae
497786348	WP_010100532.1	Verminephrobacter aporrectodeae
121608031	YP_995838.1	Verminephrobacter eiseniae EF01-2
518992821	WP_020148696.1	Verrucomicrobia bacterium SCGC AAA164-E04
518994481	WP_020150356.1	Verrucomicrobia bacterium SCGC AAA164-M04
497650275	WP_009964459.1	Verrucomicrobium spinosum
491532141	WP_005389764.1	Vibrio
515634320	WP_017066920.1	Vibrio
491535889	WP_005393509.1	Vibrio alginolyticus
516431836	WP_017820891.1	Vibrio alginolyticus
491520228	WP_005377859.1	Vibrio alginolyticus
543942278	YP_008534734.1	Vibrio alginolyticus NBRC 15630 = ATCC 17749
493934733	WP_006879133.1	Vibrio brasiliensis
494017926	WP_006960253.1	Vibrio coralliilyticus
545088859	WP_021458590.1	Vibrio coralliilyticus
518104195	WP_019274403.1	Vibrio coralliilyticus
515140283	WP_016769030.1	Vibrio cyclitrophicus
491582333	WP_005439903.1	Vibrio harveyi
516428661	WP_017818023.1	Vibrio harveyi
491587938	WP_005445505.1	Vibrio harveyi
515757698	WP_017190298.1	Vibrio harveyi
520914485	WP_020333877.1	Vibrio natriegens
490547290	WP_004412414.1	Vibrio orientalis
545083753	WP_021453658.1	Vibrio parahaemolyticus
491629120	WP_005486659.1	Vibrio parahaemolyticus
559776505	WP_023584276.1	Vibrio parahaemolyticus
545079194	WP_021449179.1	Vibrio parahaemolyticus
491637933	WP_005495462.1	Vibrio parahaemolyticus
546181669	WP_021822372.1	Vibrio parahaemolyticus
491597497	WP_005455059.1	Vibrio parahaemolyticus
545081610	WP_021451525.1	Vibrio parahaemolyticus
545077647	WP_021447644.1	Vibrio parahaemolyticus
573516901	ETT16681.1	Vibrio parahaemolyticus 50
567327501	ETJ88327.1	Vibrio parahaemolyticus 970107
433657537	YP_007274916.1	Vibrio parahaemolyticus BB22OP
525958546	YP_008316377.1	Vibrio parahaemolyticus O1:K33 str. CDC_K4557
525951248	YP_008313589.1	Vibrio parahaemolyticus O1:Kuk str. FDA_R31
28898104	NP_797709.1	Vibrio parahaemolyticus RIMD 2210633
584470631	AHI99851.1	Vibrio parahaemolyticus UCM-V493
582092769	EVU08416.1	Vibrio parahaemolyticus VP2007-007
589871373	EXJ47096.1	Vibrio parahaemolyticus VPTS-2010_2
545467482	WP_021703983.1	Vibrio proteolyticus
495354569	WP_008079290.1	Vibrio sinaloensis
491612308	WP_005469865.1	Vibrio sp. 16
516229990	WP_017633953.1	Vibrio sp. 712i1
262394444	YP_003286298.1	Vibrio sp. Ex25
497383184	WP_009697397.1	Vibrio sp. HENC-01
408889958	EKM28222.1	Vibrio sp. HENC-02
497391725	WP_009705938.1	Vibrio sp. HENC-03
490884170	WP_004746139.1	Vibrio tubiashii
560954576	XP_006199784.1	Vicugna pacos
518207255	WP_019377463.1	Virgibacillus halodenitrificans
573589845	ETT88049.1	Viridibacillus arenosi FSL R5-213
573583844	ETT82128.1	Viridibacillus arenosi FSL R5-213
573589844	ETT88048.1	Viridibacillus arenosi FSL R5-213
518801679	WP_019957633.1	Vitreoscilla stercoraria
518804175	WP_019960129.1	Woodsholea maritima
517200399	WP_018389217.1	Xanthobacteraceae
517202031	WP_018390849.1	Xanthobacteraceae
517200077	WP_018388895.1	Xanthobacteraceae
517201424	WP_018390242.1	Xanthobacteraceae
517201428	WP_018390246.1	Xanthobacteraceae
517200397	WP_018389215.1	Xanthobacteraceae
517202028	WP_018390846.1	Xanthobacteraceae
285018733	YP_003376444.1	Xanthomonas albilineans GPE PC73
515413019	WP_016902003.1	Xanthomonas arboricola
573458424	GAE54544.1	Xanthomonas arboricola pv. pruni MAFF 301420
515724482	WP_017157082.1	Xanthomonas axonopodis
21242966	NP_642548.1	Xanthomonas axonopodis pv. citri str. 306
21243283	NP_642865.1	Xanthomonas axonopodis pv. citri str. 306
346725420	YP_004852089.1	Xanthomonas axonopodis pv. citrumelo F1
498064049	WP_010378205.1	Xanthomonas campestris
21231852	NP_637769.1	Xanthomonas campestris pv. campestris str. ATCC 33913
384428348	YP_005637707.1	Xanthomonas campestris pv. raphani 756C
78048304	YP_364479.1	Xanthomonas campestris pv. vesicatoria str. 85-10
489580469	WP_003484915.1	Xanthomonas citri
488899872	WP_002810965.1	Xanthomonas fragariae
495241192	WP_007965955.1	Xanthomonas fuscans
495249926	WP_007974684.1	Xanthomonas fuscans
549715415	YP_008639278.1	Xanthomonas fuscans subsp. fuscans
493497261	WP_006451837.1	Xanthomonas gardneri
565807160	WP_023904362.1	Xanthomonas hortorum
518133094	WP_019303302.1	Xanthomonas oryzae

518135087	WP_019305295.1	Xanthomonas oryzae
58582764	YP_201780.1	Xanthomonas oryzae pv. oryzae KACC 10331
84624640	YP_452012.1	Xanthomonas oryzae pv. oryzae MAFF 311018
384418867	YP_005628227.1	Xanthomonas oryzae pv. oryzicola BLS256
498029257	WP_010343413.1	Xanthomonas sacchari
551355086	WP_022974447.1	Xanthomonas sp. M97
516522679	WP_017911045.1	Xanthomonas sp. SHU199
516529816	WP_017917622.1	Xanthomonas sp. SHU308
489570959	WP_003475412.1	Xanthomonas translucens
497277059	WP_009591276.1	Xanthomonas translucens
489561440	WP_003465971.1	Xanthomonas translucens
515683850	WP_017116450.1	Xanthomonas vasicola
498049300	WP_010363456.1	Xanthomonas vasicola
492836570	WP_005990524.1	Xanthomonas vesicatoria
56118398	NP_001008128.1	Xenopus (Silurana) tropicalis
551497550	XP_005800329.1	Xiphophorus maculatus
521958606	WP_020470211.1	Zavarzinella formosa
568804852	ETN96316.1	Zhouia amylolytica AD3
340619974	YP_004738427.1	Zobellia galactanivorans
295133239	YP_003583915.1	Zunongwangia profunda SM-A87
578048861	CDH15775.1	Zygosaccharomyces bailii ISA1307
408407815	P0DKB4.1	Saccoglossus kowalevskii
85544193	2AZP	Pseudomonas aeruginosa
215275650	A0AZQ0.2	Burkholderia cenocepacia HI2424
485602247	4K7X	Burkholderia multivorans ATCC 17616
485602254	4K8L	Ochrobactrum anthropi ATCC 49188
