# Supplementary material for: Phylogenetic and syntenic data support a single horizontal transference to a Trypanosoma ancestor of a prokaryotic proline racemase implicated in parasite evasion from host defences
Source: Parasit Vectors. 2015 Apr 12;8:222. doi: 10.1186/s13071-015-0829-y (PMC4417235; doi:10.1186/s13071-015-0829-y)
Supplement: Additional file 4: — Alignment of predicted amino acid sequences of PRAC pseudogenes from T. rangeli isolates of all phylogenetic lineages (TrA-TrE). Essential motifs (MCGH and MIII) are in green, active site in red (SPCGT), R1, R2 and R3 in blue and Cys91/267 in yellow. Red asterisks indicate stop codons. [file 13071_2015_829_MOESM4_ESM.pdf]

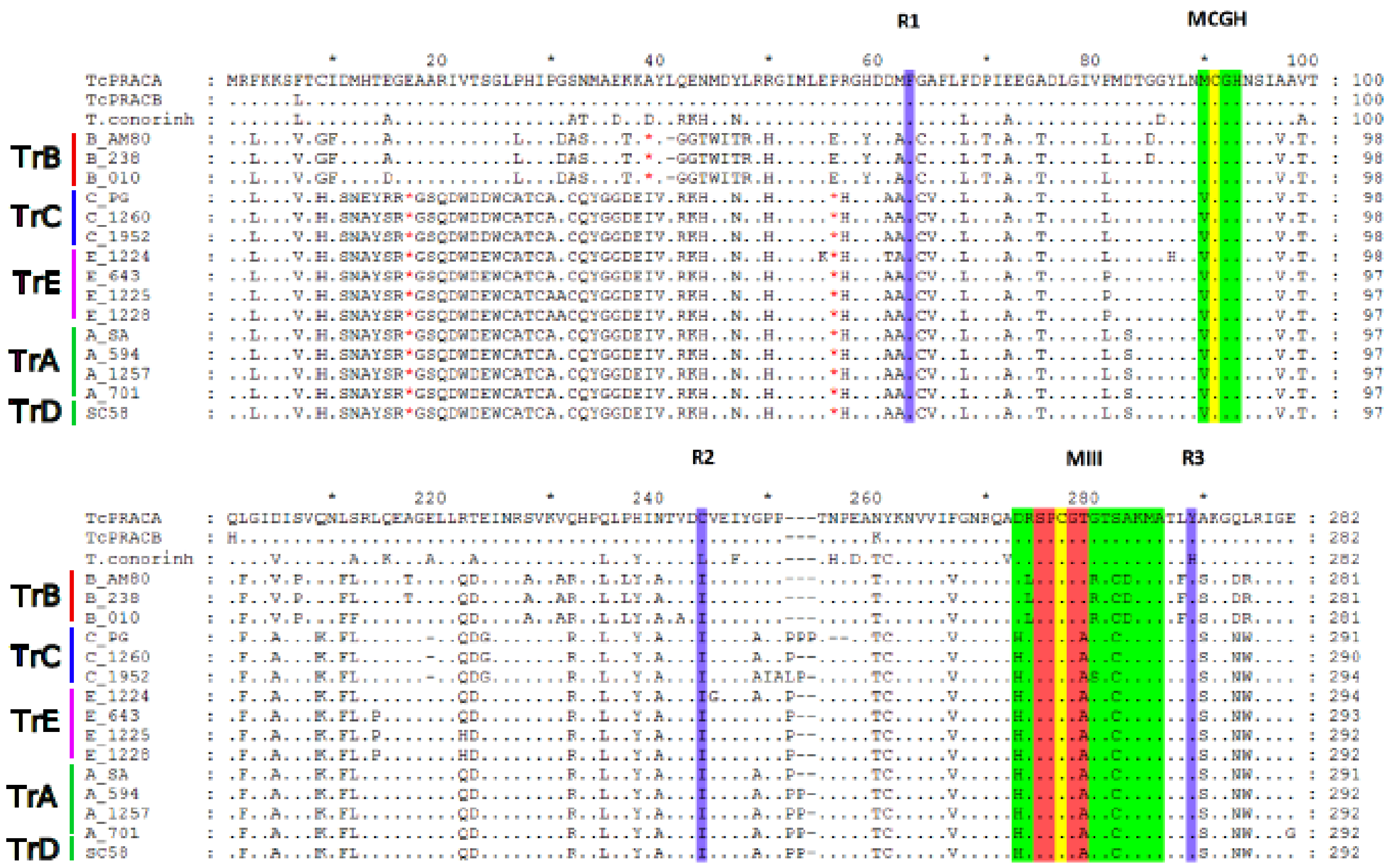
R1
MCGH
TcPRACA
TcPRACB
T.conorinh
TrB
B_AM80
B_238
B_010
TrC
C_PG
C_1260
C_1952
TrE
E_1224
E_643
E_1225
E_1228
TrA
A_SA
A_594
A_1257
A_701
TrD
SC58
R2
MIII
R3
